# Emerging Advanced Electronic Packaging Materials for Thermal Management in Power Electronics

**DOI:** 10.1002/advs.202524348

**Published:** 2026-02-16

**Authors:** Yongjun Huo, Jiaqi Song, Wenqian Li, Jian Zhang, Yujin Zhang, Yang Fu, Wangchao Yuan, Xin Chen, Sichen Liu, Miao Jiang, Yuan Cheng, Gang Zhang

**Affiliations:** ^1^ School of Materials Science and Engineering Beijing Institute of Technology Beijing China; ^2^ Yangtze Delta Region Academy in Jiaxing Beijing Institute of Technology Jiaxing China; ^3^ Monash Suzhou Research Institute Monash University Suzhou China; ^4^ Department of Materials Science and Engineering Monash University Clayton Victoria Australia

**Keywords:** ceramic substrate, multiscale simulation, power electronics, thermal interface materials, thermal management materials

## Abstract

Current research on integrated circuits and power electronics is rapidly advancing toward miniaturization, high power density, and multi‐chip integration, which presents unprecedented challenges to the thermal management performance of packaging materials. Along the device‐to‐sink heat‐flow path in power modules, thermal management relies primarily on two functional material systems: substrate materials that provide mechanical support and electrical insulation, and thermal interface materials (TIMs) that bridge heat transfer across heterogeneous interfaces. This paper summarizes recent advances in thermal management materials for power electronics, with a focus on ceramic‐based substrate systems, particularly Si_3_N_4_ ceramics, and TIM systems including conductive adhesives, diamond‐reinforced composites, and 2D filler–reinforced polymer composites. Emphasis is placed on improvements in thermal conductivity, reduction of thermal resistance, and enhancement of mechanical reliability through process optimization, interfacial engineering, and hybrid filler design. In addition, representative multiscale simulation approaches and emerging applications of artificial intelligence and machine learning are reviewed as tools for understanding interfacial heat transport and accelerating materials screening and optimization. Finally, key challenges and future directions toward scalable, reliable, and intelligent thermal management solutions are discussed, providing guidance for both academic research and industrial deployment in next‐generation power‐electronics packaging.

## Graphical abstract

This review surveys emerging materials for thermal management in advanced electronic packaging, with emphasis on ceramic substrates and thermal interface materials. Multiscale simulations and mechanistic analyses are highlighted, alongside the emerging role of artificial intelligence in predicting thermal properties and guiding design, offering critical insights for thermal management performance in power electronics.

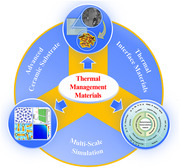

## 1 Introduction

As Moore's law approaches its physical limit, further gains in integrated‐circuit performance increasingly hinge on breakthroughs in packaging technologies for power electronics [1]. Concurrently, power electronics system are being pushed toward higher power densities and greater integration levels [2], subjecting packaging materials to unprecedented performance and reliability demands under harsh operating conditions and environment [3]. Therefore, efficient thermal management has become imperative, advanced packaging materials must therefore not only provide mechanical support and electrical interconnection, but also serve as critical pathways for rapid heat conduction and dissipation to ensure system stability and longevity [4].

The rapid development of wide bandgap semiconductors and power electronics systems has made thermal management technology a core bottleneck restricting the performance and reliability of next‐generation power equipment. Wide bandgap semiconductors possess outstanding properties, including high breakdown voltage, high electron mobility and high thermal tolerance, enabling stable operation at elevated temperatures, frequencies and voltages [5]. They are now widely applied in electric vehicles [6], renewable energy converters [7], advanced radar systems and aerospace applications [8]. However, the heat generation of such devices far exceeds that of conventional silicon‐based devices, and their performance extremely sensitive to temperature fluctuations. As a key aspect of the heat dissipation system, the thermal management capability of the thermal management material directly determines the stability and reliability of the device. With the rapid adoption of wide bandgap semiconductors and power electronics, more stringent requirements have been imposed on the thermal conductivity, thermal stability and interfacial thermal resistance (ITR) control, driving continuous innovation in both materials and structural design.

**FIGURE 1:**
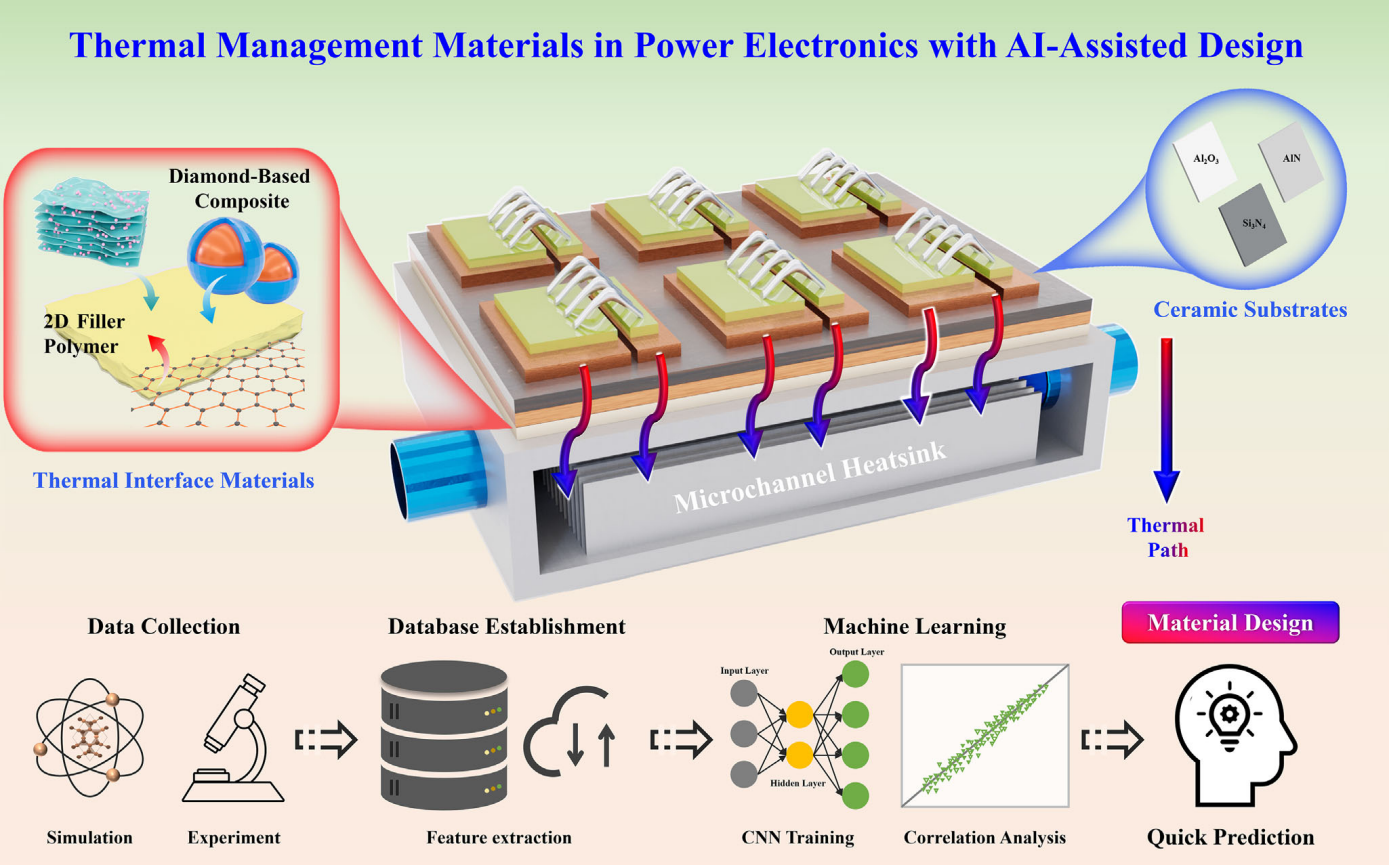
Thermal‐management pathway in a power module and an AI‐assisted materials‐design workflow.

From a device‐level perspective, heat dissipation in power modules proceeds along a continuous device‐to‐sink pathway, in which thermal management materials can be broadly classified into two functional systems: substrate materials that support chips and provide electrical insulation, and thermal interface materials that bridge thermal transport across heterogeneous layers. This functional classification provides a practical framework for organizing material development strategies in power‐electronics packaging, as schematically illustrated in Figure 1. Among substrate material systems, ceramic have become indispensable due to their combination of high thermal conductivity, mechanical robustness, and long‐term stability under extreme environments [9]. Alumina (Al_2_O_3_), aluminum nitride (AlN), and silicon nitride (Si_3_N_4_) are the most prevalent [10]. Among these candidates, Si_3_N_4_ ceramics have distinguished themselves as a new generation of highly reliable substrate materials by virtue of their exceptional fracture toughness, modulable thermal conductivity, and outstanding thermal‐shock resistance [11]. Recent studies have significantly enhanced the thermal conductivity and overall performance of Si_3_N_4_ ceramics through strategies such as powder engineering, optimized sintering technologies, and deliberate microstructural control [12, 13]. As schematically illustrated in Figure 1, such ceramic substrates form a critical segment of the heat‐flow path from the power device toward the external heatsink.

Thermal interface materials (TIMs) constitute the second key functional system along the heat‐flow path by mitigating contact resistance between dissimilar materials. Diamond‐based composites have gained prominence owing to its outstanding thermal conductivity and low thermal expansion coefficient (CTE) [14]. Diamond‐reinforced TIMs can effectively alleviate thermal‐stress accumulation, and recent studies on diamond‐metal and diamond‐non‐metal systems have demonstrated that interfacial engineering [15], particle‐size control [16], and anisotropic design [17] can substantially enhance effective thermal conductivity and thermal stability. In addition, 2D filler‐reinforced polymer composite have emerged as promising TIM candidates. Through polymer matrix modification and external field assisted orientation, including magnetic field and electric field, both thermal conductivity and processability can be further improved [18].

In parallel with materials development, artificial intelligence (AI) and machine learning (ML) are increasingly being integrated into thermal management research as design and optimization tools. Traditionally, the development of thermal management materials has been known with a long‐term research cycle and high cost, typically with a trial‐and‐error experimentation. By constructing structure‐property databases and applying ML or deep‐learning algorithms, correlations between microstructural descriptors and thermal performance can be rapidly identified, enabling accelerated materials screening and interface optimization [19]. Recent studies have demonstrated promising predictive and optimization capabilities of AI‐assisted approaches in ceramics, adhesives and composite materials [20, 21]. The lower panel of Figure 1 sketches such an AI‐assisted workflow, linking experimental and simulation data with rapid property prediction for power‐electronics packaging materials.

Despite notable progress, significant challenges remain. Thermal conductivity remains strongly dependent on microstructural characteristics [22], while interfacial bonding strength in TIM layers must be further improved to meet the reliability demands of long‐term operation [23]. In addition, achieving an optimal balance among thermal performance, mechanical robustness, environmental stability, and manufacturability remains difficult, particularly under severe thermal cycling and high‐power operating conditions. Another significant issue is the difficulty in achieving an optimal balance between thermal stability and reliability under extreme environmental conditions.

Within the framework outlined in Figure 1, this review synthesizes recent advances in ceramic substrate systems and TIMs, together with representative multiscale simulation and AI‐assisted design approaches that support their optimization. Particular emphasis is placed on structural design principles, interfacial engineering, and performance‐optimization strategies that link material selection with device‐level heat‐flow paths. Accordingly, the following sections move from substrate‐level ceramic materials, through thermal interface architectures and composite formulations, to multiscale simulation and AI‐assisted design methods, and finally to an outlook on remaining challenges and future research directions. The overarching aim is to provide both a mechanistic foundation and practical guidance for the development of next‐generation thermal management materials for power electronics packaging.

## 2 Advanced Ceramic Materials for Substrates

In power‐electronics packaging, substrate materials form the structural and functional foundation of power modules. Positioned between the semiconductors die and the external heat sink, substrates must simultaneously provide mechanical support, electrical insulation, and an efficient heat‐conduction path, thereby exerting a direct influence on thermal stability and long‐term reliability under harsh service conditions. A variety of substrate material systems have been explored in electronic packaging. Organic laminates such as Flame‐Retardant 4 (FR‐4) glass fiber‐reinforced epoxy, Bismaleimide Triazine (BT) resin and Ajinomoto Build‐up Film (ABF) offer low cost and high design flexibility [24, 25, 26]. However, their inherently thermal conductivity, typically below 1 W·m^−1^·K^−1^, is insufficient to meet the heat dissipation requirements of high‐power‐density electronic devices. Conventional metal substrates, predominantly copper (Cu) and aluminum (Al), exhibit relatively high thermal conductivity but suffer from poor electrical insulation and mismatched CTEs with wide‐bandgap devices and associated metallization layers.

Against this background, advanced ceramic substrates occupy a distinct and increasingly indispensable design space. They combine electrical insulation with structural robustness and effective heat conduction, while maintaining stability under elevated temperature, thermal cycling, and complex operating environments. This combination is particularly relevant for emerging applications in electric vehicles, renewable‐energy converters, advanced radar systems, and aerospace electronics, where substrates are required to deliver high thermal conductivity, exceptional mechanical strength, and outstanding electrical insulation [27].

To facilitate a module‐level comparison of mainstream substrate systems, representative thermal, mechanical, and electrical properties are summarized in Table 1. Importantly, Table 1 also indicates that substrate selection is not determined by any single metric. Instead, it is governed by coupled engineering requirements, including thermal conductivity, electrical insulation, mechanical robustness, coefficient of thermal expansion matching, and reliability under power cycling. Consequently, practical material choice is often dictated by trade‐offs among these attributes rather than by peak performance in one dimension. Among commercially deployed ceramic substrates, Al_2_O_3_, AlN, and Si_3_N_4_ are the most prevalent. Al_2_O_3_ benefits from mature processing and low cost, yet its limited thermal conductivity becomes increasingly restrictive as heat flux rises [28]. AlN exhibits higher intrinsic thermal conductivity and is widely adopted in power packages, but concerns remain regarding brittleness, thermal‐shock resistance, particularly under demanding power‐cycling conditions [29].

**TABLE 1:** Comparison of performance of various substrate materials.

Type	Materials	Density [g/cm^3^]	Thermal conductivity [W·m^−1^·K^−1^]	Bending strength [MPa]	Thermal expansion coefficient [10^−6^·K^−1^]	Dielectric constant @1MHz	Dielectric strength [kV·mm^−1^]
Polymer	FR‐4	1.7 [30]	<1 [30]	415 [30]	<60 [30]	5.5 [30]	>40 [30]
	BT	2.0 [30]	<1 [30]	180–280 [30]	10–15 [30]	2.5–3.5 [30]	>40 [30]
	ABF	1.8 [30]	<1 [30]	150–250 [30]	12–18 [30]	3.5 [30]	>40 [30]
Metal	Cu	8.93 [31]	380–400 [32]	∼600 [31]	17.7 [31]	N/A	N/A
	Al	2.7 [33]	237 [34]	∼200 [33]	23 [33]	N/A	N/A
Ceramic	BeO	2.9 [35]	150–310 [36, 37]	189.7 [38]	5.4 [37]	5 [35]	10 [35]
	SiC	3.2 [35]	270 [37]	487 [39]	3.13 [37]	40 [35]	27 [35]
	Al_2_O_3_	3.9 [35]	14–37 [40, 41]	200–470 [42]	6.5–7.2 [43]	9.3 [44]	12.3 [45]
	AlN	3.3 [35]	159–219 [46, 47]	267–484 [48, 49]	2.7–4.6 [43]	8.8 [50]	43.6 [51]
	Si_3_N_4_	3.2 [35]	60–177 [52, 53)	519–912 [54, 55]	2.3–3.2 [43]	9.0 [56]	57.9 [57]

Abbreviation: N/A, Not Applicable.

In contrast, Si_3_N_4_ ceramics have garnered increasing attention as a next‐generation substrate material. Its high fracture toughness and excellent thermal‐shock resistance provide strong reliability advantages, while its thermal conductivity, commonly around 80 W·m^−1^·K^−1^ in practical modules, is sufficient for many high‐reliability power‐electronics scenarios, including silicon carbide (SiC)/gallium nitride (GaN) device platforms [58].

However, achieving high thermal conductivity in Si_3_N_4_ ceramics is nontrivial. Heat transport in Si_3_N_4_ ceramics is highly sensitive to several microstructural factors, including grain boundary phases, porosity, grain morphology, crystallographic orientation, and residual oxygen content [59, 60, 61]. These, in turn, are governed by powder characteristics, such as particle size distribution, α/β phase content, and impurity levels, as well as by sintering strategies and the nature of the sintering additives.

Accordingly, this section first establishes the substrate‐level property trade‐offs that motivate the growing interest in advanced ceramic systems, and then provides a focused and critical discussion of Si_3_N_4_ as a representative high‐reliability substrate. The following subsections review key strategies for thermal‐conductivity enhancement, including powder engineering, sintering‐route optimization, additive design, and texture control, and briefly discuss emerging data‐driven approaches for performance prediction and process guidance.

### 2.1 Conventional Ceramic Materials for Substrate

Al_2_O_3_ currently represents the most extensively utilized substrate materials within the ceramic category. Exhibiting cost‐effectiveness, high mechanical strength, exceptional wear resistance, and superior electrical insulation performance, they are predominantly employed in mid‐to‐low‐end application scenarios including light‐emitting diode (LED) thermal management substrates and thick‐/thin‐film circuit substrates [62, 63]. Of the principal polymorphs, namely, α‐, β‐, and γ‐Al_2_O_3_, the α phase possesses the highest density and outstanding thermal stability. Heat conduction in Al_2_O_3_ substrates proceeds chiefly through phonon transport, well approximated by a ball‐and‐spring model. Low atomic mass, sparse atomic packing, and strong covalent bonding jointly enhance phonon propagation and thus thermal conductivity [40]. The reported thermal conductivity ranges from 14 to 37 W·m^−1^·K^−1^, while mechanical properties remain suboptimal [64]. As downstream applications evolve toward high‐frequency, high‐power, and miniaturized requirements, Al_2_O_3_ ceramics fail to meet these high‐end demands and are increasingly being replaced.

The theoretical thermal conductivity of AlN reaches 320 W·m^−1^·K^−1^, approximately tenfold that relative to aluminum oxide [65]. It exhibits high mechanical strength and electrical insulation properties, making it suitable for high‐power electronic devices. Their elevated melting point, however, hampers full densification, yielding residual porosity. in addition, AlN readily oxidizes to Al_2_O_3_ and, as a polycrystal, contains numerous grain boundaries that curtail phonon mean free paths, thwarting attainment of the material's theoretical thermal conductivity [40, 66]. Several primary challenges for AlN must be addressed. First, mechanical properties require improvement through enhanced relative density, reduced grain size, and uniform distribution of the grain boundary phase. Second, employing high‐purity raw materials and selecting efficient sintering aids is essential to minimize lattice oxygen content, thereby augmenting the material's thermal conductivity. Third, focus on solving the technical difficulties in the production process, and resolve the manufacturing challenges and yield issues to lay a foundation for large‐scale commercial applications.

In additional, beryllium oxide (BeO) ceramic substrates exhibit outstanding thermal conductivity, reaching up to 310 W·m^−1^·K^−1^ under ambient conditions [36]. Meanwhile, BeO offers exceptional dielectric properties, mechanical strength, and electrical insulation, making it widely regarded as the most versatile ceramic substrate material. However, its production poses significant environmental concerns due to the release of toxic substances, failing to meet basic green manufacturing standards. Consequently, it is being progressively replaced by more eco‐friendly alternatives. SiC ceramics exhibit an exceptionally high thermal conductivity, with values reaching up to 270 W·m^−1^·K^−1^ [67]. However, their relatively high dielectric constant induces signal propagation delay, thereby compromising product reliability and restricting their applicability in high‐frequency electronic applications.

### 2.2 Silicon Nitride Ceramic Material for Substrate

Si_3_N_4_ has recently emerged as a strategic substrate for high‐reliability systems that demand both thermal efficiency and mechanical resilience. With meticulous processing and microstructural control, its thermal conductivity can attain 80–130 W m^−1^ K^−1^ while preserving exceptional fracture toughness and thermal‐shock resistance, surpassing Al_2_O_3_ and AlN. These qualities make Si_3_N_4_ the material of choice for automotive IGBT modules, aerospace electronics, wind‐power inverters, and high‐temperature SiC/GaN packages, where endurance under thermomechanical stress is critical [68].

Research on Si_3_N_4_ as a high thermal conductivity material began with Haggerty et al. [69] Their study established that the theoretical thermal conductivity of β‐Si_3_N_4_ single crystals could reach approximately 320 W·m^−1^·K^−1^. In recent years, Si_3_N_4_ has become a strategic substrate material for high‐reliability electronic systems. Currently, the highest reported thermal conductivity of lab‐prepared Si_3_N_4_ ceramics is 177 W·m^−1^·K^−1^ [70], whereas commercial ceramic substrates typically exhibit thermal conductivity values exceeding 80 W·m^−1^·K^−1^. Additionally, the unique interlocking microstructure and bimodal grain size distribution of Si_3_N_4_ ceramics impart superior mechanical properties compared to Al_2_O_3_ and AlN. This enables thinner substrate thicknesses and thicker Cu‐clad layers, thus significantly lowering the system's thermal resistance.

**FIGURE 2:**
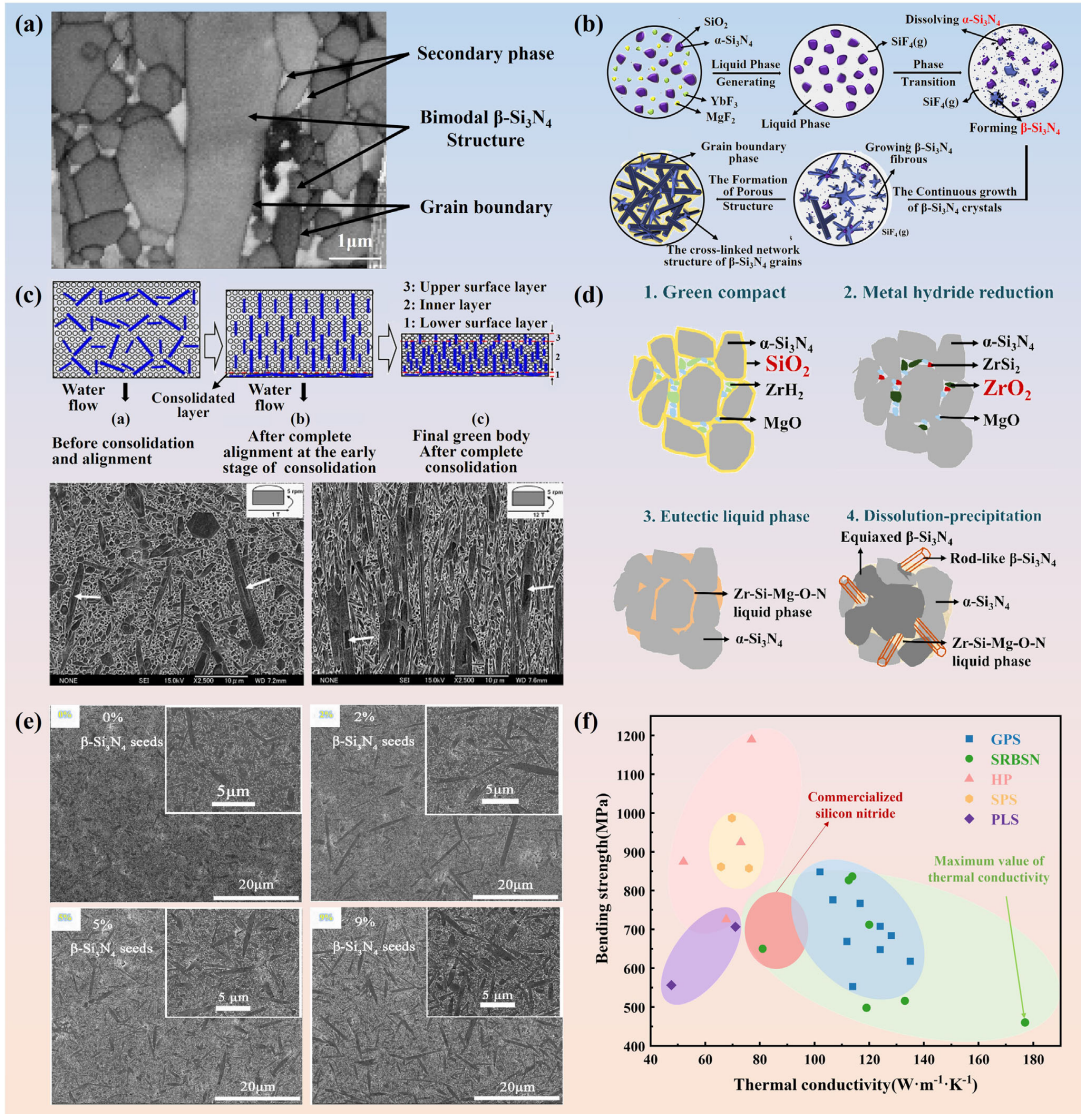
Microstructural control strategies for high‐performance Si_3_N_4_ ceramics: (a) Typical post‐sintering microstructure with elongated β grains. (b) Schematic of grain evolution via α→β transformation and anisotropic growth. Reproduced with permission. [76]. Copyright 2019, Elsevier Ltd and Techna Group S.r.l. (c) Magnetic field‐induced crystallographic alignment. Reproduced with permission. [78]. Copyright 2009, Elsevier Ltd. (d) ZrH_2_‐assisted in situ reduction and densification mechanism. Reproduced with permission. [79]. Copyright 2020, Elsevier B.V. (e) Bimodal microstructure promoted by β‐Si_3_N_4_ seed whiskers.Reproduced with permission. [12] Copyright 2019, Elsevier Ltd. (f) The correlation between thermal conductivity and flexural strength of Si_3_N_4_ ceramics fabricated via distinct sintering techniques. GPS, gas pressure sintered [80, 81, 82, 83, 84, 85, 86, 87, 88]; SRBSN, sintered reaction‐bonded silicon nitride [13, 70, 89, 90, 91, 92, 93]; HP, hot pressing [94, 95, 96, 97]; SPS, spark plasma sintering [98, 99, 100]; PLS, pressureless sintering [101, 102].

Si_3_N_4_’s outstanding thermal management capacity renders it indispensable for high‐power electronic packaging. Whereas electron transport governs heat conduction in metals, phonon propagation dominates its efficiency in ceramics. Hence, its thermal conductivity mainly depends acutely on microstructural and chemical attributes. In Si_3_N_4_ ceramic sintered bodies, β‐Si_3_N_4_ predominates as the primary crystalline phase. The thermal conductivity of these ceramics is primarily governed by three factors: lattice oxygen content, β‐Si_3_N_4_ grain size, and the grain boundary phase. Among these, lattice oxygen content constitutes the predominant factor crucially determining thermal conductivity [71]. Using a hot gas extraction technique, Kitayama et al. measured oxygen content in the lattice and demonstrated that point defects from oxygen dissolution in β‐Si_3_N_4_ govern thermal conductivity [61]. Figure 2a displays the typical sintered microstructure [72]. Oxygen segregates to grain boundaries and intergranular glassy films, generating amorphous silicates that scatter phonons [73, 74]. Mitigation therefore requires ultra‐pure powders, rigorously controlled atmospheres, and additives that consume or crystallize these phases. Equally decisive is boundary crystallinity, where fully or partially crystallized interfaces reduce thermal boundary resistance [75].

Phase evolution in the Si_3_N_4_ sintering process, as presented in Figure 2b, typically involves α‐ to β‐ transformation via liquid‐phase‐assisted dissolution reprecipitation, grain boundary crystallization, and densification, yielding aligned β‐ Si_3_N_4_ grains with thin crystalline interphases for enhanced thermal transport [76]. The formation of elongated, aligned β‐Si_3_N_4_ grains with crystallized intergranular films lays a microstructural foundation for exploiting its inherent anisotropy. Thermal conductivity in β‐Si_3_N_4_ is inherently anisotropic. Rod‐shaped β grains conduct heat most efficiently along their longitudinal axes. Aligning them with the heat‐flow direction markedly elevates bulk conductivity. Finally, porosity, particularly closed pores, must be rigorously suppressed, as even sub‐micrometric voids severely disrupt thermal pathways [77].

### 2.3 Silicon Nitride Powder Design and Sintering Routes

Beyond comparing substrate candidates, attention is directed to strategies that further enhance Si_3_N_4_ performance. The thermal and mechanical performance of Si_3_N_4_ ceramics hinges on precursor purity and sintering sophistication. Current protocols favor high‐purity α‐Si_3_N_4_ powders with negligible surface oxidation, tight particle‐size distributions, and tailored morphologies to accelerate densification and suppress interfacial defects [103]. Doping β‐Si_3_N_4_ seed into the initial α‐Si_3_N_4_ powder can further promote the in situ α→β transformation and guide the growth of anisotropic grains, as shown in Figure 2e. Currently, there are limited reports on the preparation of seed. For instance, Peelamedu D. Ramesh et al. [104] mixed α‐Si_3_N_4_ powder with 0.5 wt.% Y_2_O_3_, forming a Y‐Si‐O‐N liquid phase at 1800°C under 35 kPa nitrogen pressure, which precipitated numerous seed with an average aspect ratio of 4 upon cooling. In another approach, Horng‐Hwa Lu et al. [105] combined α‐Si_3_N_4_, Y_2_O_3_, and Al_2_O_3_, sintering the mixture into dense blocks at 1850°C under 1 MPa nitrogen pressure in a graphite furnace, followed by crushing to obtain β‐Si_3_N_4_ seed.

Gas‐pressure sintering (GPS) remains the industrial mainstay, furnishing thermal conductivities near 120 W·m^−1^·K^−1^, while hot‐isostatic pressing (HIP) and hot pressing (HP) eliminates residual porosity and fortifies intergranular cohesion, further elevating performance [106]. Spark‐plasma sintering (SPS), with its ultrafast heating and field‐assisted densification, is increasingly adopted to curb grain coarsening and enable non‐oxide sintering chemistries [107]. Among emergent routes, sintered reaction‐bonded silicon nitride (SRBSN) is especially compelling. This two‐stage process first nitrides elemental silicon into a porous Si_3_N_4_ scaffold, then consolidates it via secondary sintering, producing complex net‐shaped bodies and highly interconnected β‐Si_3_N_4_ networks with thermal conductivities surpassing 140 W·m^−1^·K^−1^ in optimized systems [108]. Variants such as oscillatory‐pressure sintering, hot‐pressing flowing sintering (HPFS) and post‐sintering annealing offer additional levers for microstructural refinement. Figure 2f illustrates the correlation between thermal conductivity and flexural strength of Si_3_N_4_ ceramics fabricated via distinct sintering techniques [109].

GPS and SRBSN offer significant advantages in fabricating Si_3_N_4_ ceramics with high thermal conductivity. However, governed by grain growth mechanisms, high thermal conductivity is often achieved at the expense of mechanical strength. Currently, commercial silicon nitride substrates typically exhibit thermal conductivities in the range of 80–100 W·m^−1^·K^−1^ and flexural strengths of 600–800 MPa. Regarding mass production, HP is a well‐established technology. In contrast, SPS is ill‐suited for industrial substrate production due to high equipment and processing costs, while pressureless sintering (PLS) fails to meet requirements due to limited densification and performance. Consequently, HP, GPS, and SRBSN represent the mainstream technical routes for the future manufacturing of high‐performance ceramic substrates.

Magnetic field‐assisted processing offers an innovative strategy for fabricating textured Si_3_N_4_ ceramics, leveraging the diamagnetic anisotropy inherent in β‐Si_3_N_4_ whiskers [78, 110, 111]. In this technique, a rotating high‐strength magnetic field (≥10 T) exerts torque on the whiskers, aligning their c‐axes parallel to the magnetic field direction. Subsequent sintering consolidates this alignment, resulting in ceramics with distinct c‐axis texture. The orientation dynamics of whiskers within the rotating magnetic field are governed by multiple parameters, including magnetic flux density, suspension viscosity, magnetic field rotation speed, whisker aspect ratio, and initial orientation state. Zhu et al. [78] conducted research showing that, for β‐Si_3_N_4_ whiskers with a diameter of 0.4 µm and an aspect ratio of 10 under a 12 T magnetic field, the calculated anisotropic energy is 2.88 × 10^−19^ J, whereas the thermal energy at ambient condition is 4.11 × 10^−21^ J (Figure 2c). As the anisotropic energy of the whiskers under the strong magnetic field is significantly greater than the thermal energy, the whiskers undergo rotation. During the grain growth process, the β‐Si_3_N_4_ whiskers oriented by the magnetic torque effect absorb surrounding α‐Si_3_N_4_ grains along the c‐axis direction via a dissolution‐precipitation mechanism, thereby achieving epitaxial growth. Simultaneously, α‐Si_3_N_4_ grains in other regions are in situ transformed into randomly oriented β‐Si_3_N_4_ grains. This pre‐aligned architecture serves as a microstructural scaffold that directs anisotropic grain growth during sintering. The resulting crystallographic texture minimizes phonon‐boundary scattering along aligned grains, enhancing in‐plane thermal conductivity. Simultaneously, elongated grains act as toughening bridges, improving fracture resistance. In addition, c‐axis textured Si_3_N_4_ ceramics can also be prepared via processes such as tape casting [112], extrusion [113], hot pressing [68], and spark plasma sintering [114]. Among these techniques, the first two methods achieve orientation by aligning β‐Si_3_N_4_ powders along specific template directions during the forming stage, whereas the latter two promote directional grain alignment through the application of external loads, thereby completing the texturing transformation.

### 2.4 Grain‐Boundary and Phase‐Interface Control

In Si_3_N_4_‐based substrates, thermal transport and mechanical reliability are both strongly governed by the chemistry and structure of grain‐boundary phases and internal phase interfaces, which control phonon scattering as well as crack initiation and propagation. The densification sintering of Si_3_N_4_ requires the introduction of sintering aids to react with the SiO_2_ on the surface of Si_3_N_4_ powder, thereby generating a liquid phase. Early systems leaned on oxide additives such as Y_2_O_3_, Al_2_O_3_, and MgO to generate transient liquid phases, yet these agents also leave silicate glass at grain boundaries, undermining thermal conductivity. Contemporary research therefore favors non‐oxide additives, such as MgSiN_2_, rare‐earth silicon oxynitrides, reductive metal hydrides (e.g., ZrH_2_), and boride‐ or carbide‐containing blends. Table 2 lists the properties of Si_3_N_4_ ceramics with non‐oxide sintering additives added in recent years. These next‐generation additives exhibit minimal reactivity with residual SiO_2_, superior thermal‐expansion compatibility, and the ability to form wholly crystalline boundary phases. High‐content MgSiN_2_ additions (e.g., 5 mol%) enable full densification of Si_3_N_4_ ceramics while inhibiting excessive grain growth. As shown in Figure 2d, the incorporation of ZrH_2_ as a reactive sintering aid facilitates the in situ reduction of native SiO_2_ impurities, thereby markedly lowering the oxygen activity within the system [79]. Subsequent formation of an oxygen‐lean Zr‐Mg‐Si‐O‐N liquid phase promotes the dissolution‐reprecipitation‐driven growth of elongated β‐Si_3_N_4_ grains and the purification of grain interiors.

**TABLE 2:** Properties of Si_3_N_4_ ceramics with non‐oxide sintering additives added.

Additive	Sintering method	Thermal conductivity [W·m^−1^·K^−1^]	Bending strength [MPa]	Fracture toughness [MPa·m^1/2^]	Year
Y_2_O_3_+MgO+MgSiN_2_	GPS	105.2	726.1	5.9	2025 [115]
Gd_3_Si_2_C_2_+MgO	GPS	101.9	848.3 ± 4.7	6.2 ± 0.2	2025 [82]
Y_2_Si_4_N_6_C+MgSiN_2_	GPS	116.7	767 ± 29.6	10.7 ± 0.4	2024 [83]
MgSi_2_	PAS	110	705 ± 30	9.6 ± 0.1	2023 [116]
YF_3_+MgF_2_	GPS	83	823 ± 22	8.8 ± 0.1	2022 [117]
Y_2_O_3_+MgSiN_2_	GPS	91.9	926	8.4	2021 [56]
GdH_2_+MgO	GPS	135	618	/	2021 [87]
ZrH_2_+MgO	GPS	116.4	/	/	2021 [79]

Abbreviations: GPS, Gas Pressure Sintering; PAS, Plasma‐Activated Sintering.

**FIGURE 3:**
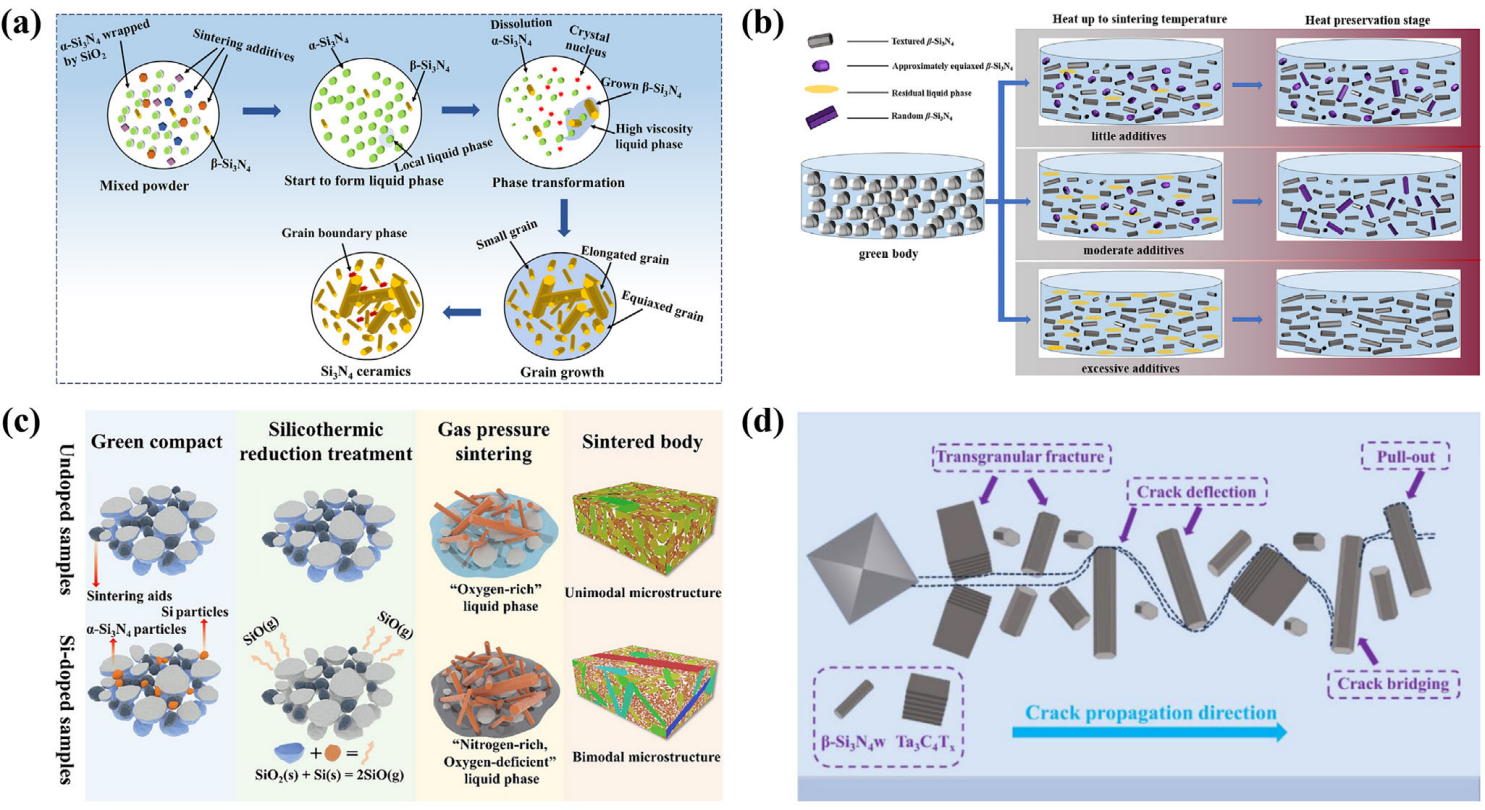
(a) The schematic diagram illustrating the hindering effect of high viscosity liquid phase on grain growth. Reproduced with permission. [115]. Copyright 2025, Elsevier Ltd and Techna Group S.r.l. (b) Texture evolution mechanism of the Si_3_N_4_ ceramics with different additive amounts. Reproduced with permission. [118]. Copyright 2023, Elsevier Ltd and Techna Group S.r.l. c) Schematic description of the effects of silicothermic reduction treatment on microstructure evolution of Si_3_N_4_ ceramics. Reproduced with permission. [120]. Copyright 2025, The American Ceramic Society. d) Schematic diagram of the toughening mechanism of β‐Si_3_N_4_ . Reproduced with permission. [121]. Copyright 2024, Elsevier Ltd and Techna Group S.r.l.

Sintering aids are intricately associated with the properties of ceramics. As illustrated in Figure 3a, excessive viscosity in the liquid phase formed by sintering additives hinders grain growth. Such elevated viscosity causes localized enrichment of the liquid phase, thereby suppressing β‐Si_3_N_4_ precipitation and preventing the development of a bimodal microstructure [115]. Moreover, the amount of sintering additives directly influences both the quantity and viscosity of the liquid phase. These factors collectively determine the preferential precipitation sites and rate of silicon and nitrogen atoms onto β‐Si_3_N_4_ grains, ultimately governing their preferential orientation growth (Figure 3b) [118].

Furthermore, incorporating silicon powder into the raw material mixture absorbs oxygen and facilitates the formation of coarse β‐Si_3_N_4_ grains, enabling the achievement of a bimodal grain structure. For instance, blending 30–50 wt.% coarse silicon powder with fine silicon powder optimizes ceramic properties [119]. The silicon thermal reduction treatment simultaneously establishes a N‐rich/O‐poor liquid‐phase environment (Figure 3c). This environment refines β‐Si_3_N_4_ grains and triggers their abnormal growth, thereby optimizing the microstructure [120].

Recent research has repositioned β‐Si_3_N_4_ seed whiskers from passive nucleants to active microstructural design tools that enable precise control over grain morphology and phase evolution [121, 122]. By serving as selective templates for anisotropic grain growth, these whiskers promote the in situ formation of elongated β‐phase grains, thereby engineering a bimodal microstructure that synergistically combines crack deflection, grain bridging, and pull‐out mechanisms (Figure 3d) [121]. These features underpin the observed enhancement in fracture toughness without compromising matrix strength. Simultaneously, the elongated grains serve as preferential phonon pathways due to their low phonon‐boundary scattering characteristics along the c‐axis, facilitating directional thermal transport. However, the thermal benefits of such grain architectures are contingent upon suppressing interfacial phonon scattering, which is achieved through careful control of oxygen content within the β‐Si_3_N_4_ lattice and the stabilization of ultra‐thin, chemically compatible amorphous boundary films.

Beyond thermal optimization, the emerging frontier aims to confer simultaneous mechanical compliance and strength upon inherently brittle ceramics. In covalently bonded ceramics, tailoring coherent internal phase boundaries has been shown to enable unconventional deformation mechanisms at room temperature [123]. Specifically, engineered coherent α/β interfaces triggers bond‐switching during stress‐induced phase transformation, enabling planar, dislocation‐free slip. Such phase‐boundary‐mediated deformation concepts provide a useful perspective for substrate design in power modules, where resistance to thermomechanical damage is often as critical as thermal conductivity. While their translation to engineering ceramics and scalable processing remains at an early stage, these advances highlight the potential of phase‐interface control to mitigate the intrinsic brittleness of ceramic substrates without fundamentally compromising their thermal functionality.

Despite remarkable advances in tailoring additives, interfaces, and seed‐induced architectures, the optimization of Si_3_N_4_ substrates remains constrained by a vast and highly coupled parameter space. Conventional trial‐and‐error strategies often fail to capture hidden correlations among composition, processing, and microstructural evolution. Consequently, emerging AI‐assisted approaches offer a powerful complement, enabling predictive mapping of structure‐property relationships and accelerating the discovery of next‐generation, high‐performance ceramic substrates.

### 2.5 AI‐Assisted Design for Silicon Nitride Ceramics

To overcome the bottlenecks of high resource consumption, long cycle time, and low efficiency in optimizing the properties of Si_3_N_4_ ceramics using the traditional trial‐and‐error method, AI has provided new ideas and begun to reshape ceramic research. ML, a branch of AI, predicts outcomes by identifying and learning patterns in data without being explicitly programmed. Recent studies have accurately predicted thermal conductivity [124, 125], fracture toughness [126], and densification behavior [125] of Si_3_N_4_ based on additive chemistry, particle morphology, and sintering profiles, thereby accelerating optimization and guiding experiments toward previously unexplored yet promising domains. With its powerful data processing and pattern recognition capabilities, AI technology is moving from the accurate prediction of Si_3_N_4_ ceramic properties to the intelligent design of high thermal conductivity ceramics, driving the innovation of high‐performance thermal management materials.

**FIGURE 4:**
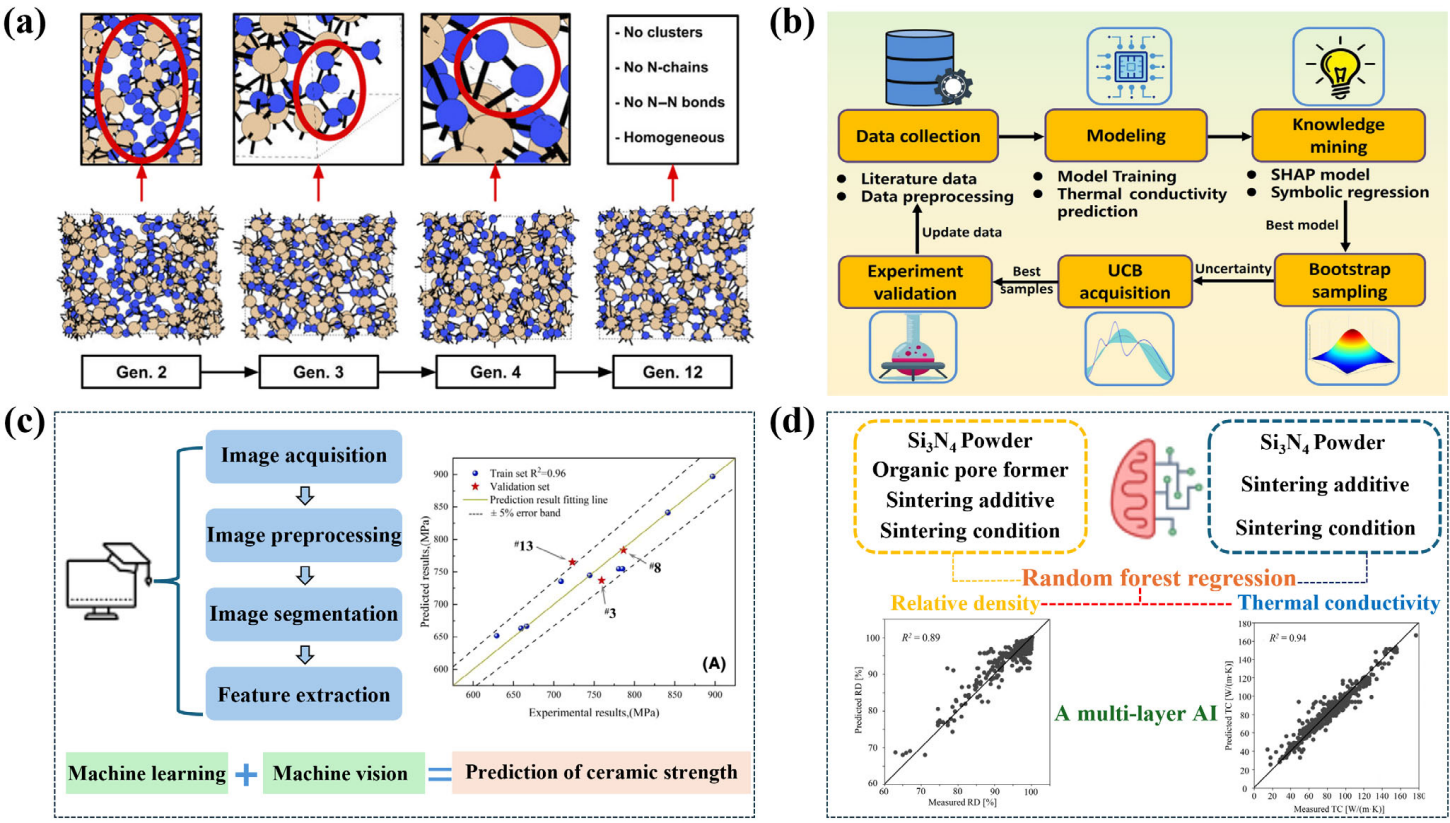
(a) Schematic of the iterative re‐training framework applied to establish the ML interatomic potential for Si_3_N_4_. Reproduced with permission. [127]. Copyright 2023, AIP Publishing. (b) Process of iterative improvement of Si_3_N_4_ ceramics thermal conductivity based on ML. Reproduced with permission. [128]. Copyright 2025, Elsevier Ltd and Techna Group S.r.l. (c) Workflow for collecting pore data on ceramic surface and comparison of predicted values and measured values for the ML strength prediction model. Reproduced with permission. [129]. Copyright 2025, The American Ceramic Society. (d) Conceptual diagram of the multi‐layered AI framework predicting thermal conductivity, with representative training results for relative density and thermal conductivity obtained from random forest regression. Reproduced with permission. [125]. Copyright 2024, Elsevier Ltd and Techna Group S.r.l.

Figure 4 illustrates emerging trends in the development of Si_3_N_4_ substrates. At the atomistic level, Milardovich et al. [127] implement an active‐learning loop for Si_3_N_4_ in which inexpensive molecular‐dynamics sampling, uncertainty screening, density‐functional labeling, and iterative retraining produce a gaussian approximation potential with near first‐principles fidelity. The resulting model achieves a mean absolute error of as low as 8 meV·atom^−1^ on liquid and amorphous sets and accelerates dynamics by three to four orders of magnitude, enabling large‐cell trajectories suitable for lattice‐dynamics and thermal‐transport analyses. Figure 4a schematizes this bootstrap cycle, providing a physics‐grounded surrogate on which multiscale design can build.

Extending from atomic modeling to property targeting, Guo et al. [128] assemble literature‐derived descriptors of composition and sintering schedule to learn mappings to thermal conductivity, then close the loop experimentally (Figure 4b). Among the nine algorithms evaluated, random forest exhibited the strongest generalization, yielding a test‐set coefficient of determination (R^2^) of approximately 0.85. Shapley additive explanations rank sintering time, temperature, and Y_2_O_3_ content as dominant levers, while an upper‐confidence‐bound active‐learning policy prioritizes candidates within feasible SPS parameters. The experimental validation process confirms thermal conductivity up to 67.5 W·m^−1^·K^−1^ in dense β‐Si_3_N_4_ with refined grain‐boundary phases, which facilitate heat flow.

Reliability at the mesoscale is addressed by Wang et al. [129], who couple machine vision with supervised regression to translate pore morphology in scanning electron microscopy (SEM) mosaics into quantitative predictors of strength (Figure 4c). After segmentation, geometric and positional descriptors are filtered via correlation analysis and used to train ensemble learners. an AdaBoost regressor attains high held‐out fidelity, with R^2^> 0.96 and an error near 3%. Interpretable outputs identify the pore‐convexity ratio as a decisive descriptor and reveal a threshold around 1.18 marking the onset of pronounced stress amplification, enabling lot‐level certification without recourse to nano‐CT.

Finally, Furushima et al. [125] propose a hierarchical scheme that respects process‐property coupling (Figure 4d). Relative density (RD) is first predicted from powders, additives, pore formers, and staged heat‐treatments. The predicted RD, together with process descriptors, is then injected into a second model for thermal conductivity. Introducing this mechanistic intermediate improves out‐of‐sample performance: the test‐set R^2^ increases from 0.75 (without RD) to 0.80 (with RD), while variable‐importance analysis highlights second‐stage time/temperature and density as top contributors. The framework supports prefabrication screening across pressureless, GPS, HP, SPS, and microwave routes, shortening optimization cycles for Si_3_N_4_ substrates.

Despite the significant advancements ML has made in predicting material properties, several limitations persist. The complex fabrication processes and high costs associated with performance testing of ceramic materials directly constrain the size of available experimental datasets. Consequently, traditional ML models applied to small datasets are prone to overfitting [130], thereby compromising predictive accuracy and generalization capabilities. Furthermore, the decision‐making processes of ML models are frequently characterized as “black boxes”: while model inputs and outputs are clearly defined, their internal operational mechanisms and decision logics remain elusive [131]. Additionally, ceramic performance is influenced by multiple factors, rendering feature selection highly challenging. Some models over‐rely on non‐empirical features lacking physical significance [132], which hinders the interpretation of relationships between prediction results and the internal physical mechanisms of materials, thereby reducing the credibility and interpretability of model predictions. A further critical issue is that ML models developed for specific performance prediction tasks often cannot be directly applied to other performance prediction scenarios, severely limiting their universality and practical utility. Looking ahead, when predicting key performance indicators such as substrate thermal conductivity and breakdown strength, ML must accurately capture the complex correlations between process parameters such as sintering temperature, pressure, holding time and substrate microstructure. Concurrently, deep integration of machine vision technology with ML will enable efficient screening of defective substrates [129], offering more reliable and effective solutions for the prediction and optimization of ceramic material properties.

In current mass production and practical applications, the typical thermal conductivity of Si_3_N_4_ substrates remains around 70–90 W/m·K. While experimental processes have achieved ≥100 W/m·K, strength and reliability remain the primary challenges. During the process of improving thermal conductivity, factors such as grain boundary glass phase residues, oxygen content, and second‐phase distribution often compromise the material's mechanical properties, preventing the bending strength from meeting the demands of high‐power applications. Consequently, the balance between high thermal conductivity, strength, and cost remains the key constraint in the commercialization of these materials. For high‐heat flux applications, such as in electric vehicles and rail transportation systems, overcoming this trade‐off will be crucial for advancing the industrialization.

While these advances substantially enhance heat transport within the substrate itself, module‐level thermal performance is ultimately governed by a series of heterogeneous contacts and intermediate layers along the device‐to‐sink heat‐flow path. As substrate thermal resistance is progressively reduced, interfacial heat transfer and compliance between dissimilar materials emerge as the dominant bottlenecks, shifting the optimization focus toward thermal interface architectures and composite interlayers, which are discussed in the following section.

## 3 Advanced Thermal Interface Materials

TIMs serve distinct roles within advanced electronic packaging systems by eliminating microscopic air gaps between electronic components and heat‐dissipation structures, thereby reducing contact thermal resistance and enabling efficient heat transfer across heterogeneous interfaces. In practical power modules, TIMs must combine low interfacial thermal resistance, mechanical compliance, and long‐term reliability under thermal cycling. Their performance is primarily evaluated through interfacial and bulk thermal conductivity. Typical examples include thermal greases and phase‐change materials [133]. In contrast, encapsulant materials are high‐performance composites that isolate chips or electronic elements from the external environment. Their core function is to protect the packaged device from environmental degradation while enhancing overall reliability. concurrently, they must possess effective heat‐dissipation capability [134].

As illustrated in Figure 1, recent TIM research in power electronics has mainly evolved along two representative material‐system directions: (i) diamond‐based composite systems, which exploit the extremely high intrinsic thermal conductivity of diamond to construct rigid, high‐flux heat‐spreading media, and (ii) polymer‐based composites reinforced with 2D nano‐fillers, which achieve efficient thermal pathways while maintaining mechanical compliance and surface conformity. Although these systems differ in matrix chemistry and mechanical characteristics, both rely on interfacial regulation and thermal‐network construction to overcome phonon scattering and contact resistance.

In addition to TIMs, encapsulant materials are also widely used in electronic packaging to protect devices from environmental degradation while contributing to heat dissipation. However, their primary function is structural protection and environmental isolation, whereas TIMs are specifically designed to minimize thermal resistance at material interfaces. Accordingly, the following discussion focuses on material design strategies that directly govern interfacial heat transfer in TIM systems, with particular emphasis on diamond‐based composites and 2D filler–reinforced polymer composites.

It should be clarified that the terms “high” and “low” thermal conductivity used throughout this review are defined relative to the material system under discussion. Polymer matrices typically exhibit very low thermal conductivity (0.1–0.5 W·m^−1^·K^−1^), while high‐conductivity fillers such as h‐BN possess intrinsic thermal conductivities ranging from 100 to 2000 W·m^−1^·K^−1^. The effective thermal conductivity of polymer composites generally falls in the range of 1–50 W·m^−1^·K^−1^. For TIMs, however, the key performance metric is the interfacial thermal conductance (ITC) (typically ≥30–100 MW·m^−2^·K^−1^), and therefore discussions in these sections focus on interfacial heat transfer rather than bulk conductivity.

### 3.1 Diamond‐Based and Filler‐Enhanced Thermally Conductive Adhesive Systems

Thermally conductive adhesives (TCAs) serve as critical packaging materials that integrate robust adhesion with efficient heat transfer. Their principal challenge is to construct continuous thermal pathways at low filler loadings [135]. Commercial products typically employ epoxy or silicone matrices filled with micron‐sized Al_2_O_3_ [136], yielding thermal conductivities of 1–5 W·m^−1^·K^−1^, sufficient for LED and consumer‐electronics applications with moderate heat fluxes.

In contrast, most recently studies have achieved substantial performance advances through the judicious selection and manipulation of novel fillers. ND, distinguished by its exceptional intrinsic thermal conductivity, becomes an ideal reinforcement after surface salinization, leading to pronounced conductivity enhancements. The incorporation of 2D transition metal carbides (MXenes) into epoxy adhesives has likewise elevated thermal conductivity, while external‐field‐assisted techniques further amplify performance gains.

This section provides a comprehensive review of TCAs for advanced packaging, with a particular focus on recent research progress in high‐performance TCAs and strategies for enhancing their thermal conductivity. The content covers the application of novel reinforcements such as ND, the introduction and network construction of 2D fillers (e.g., graphene, MXenes, and boron nitride (BN)), the enhancement of interfacial interactions through matrix modification, e.g., chemical functionalization and structural design, and the alignment of fillers assisted by external fields, e.g., magnetic and electric fields. Additionally, key technologies such as ITR regulation and multiscale structural optimization are also included.

**FIGURE 5:**
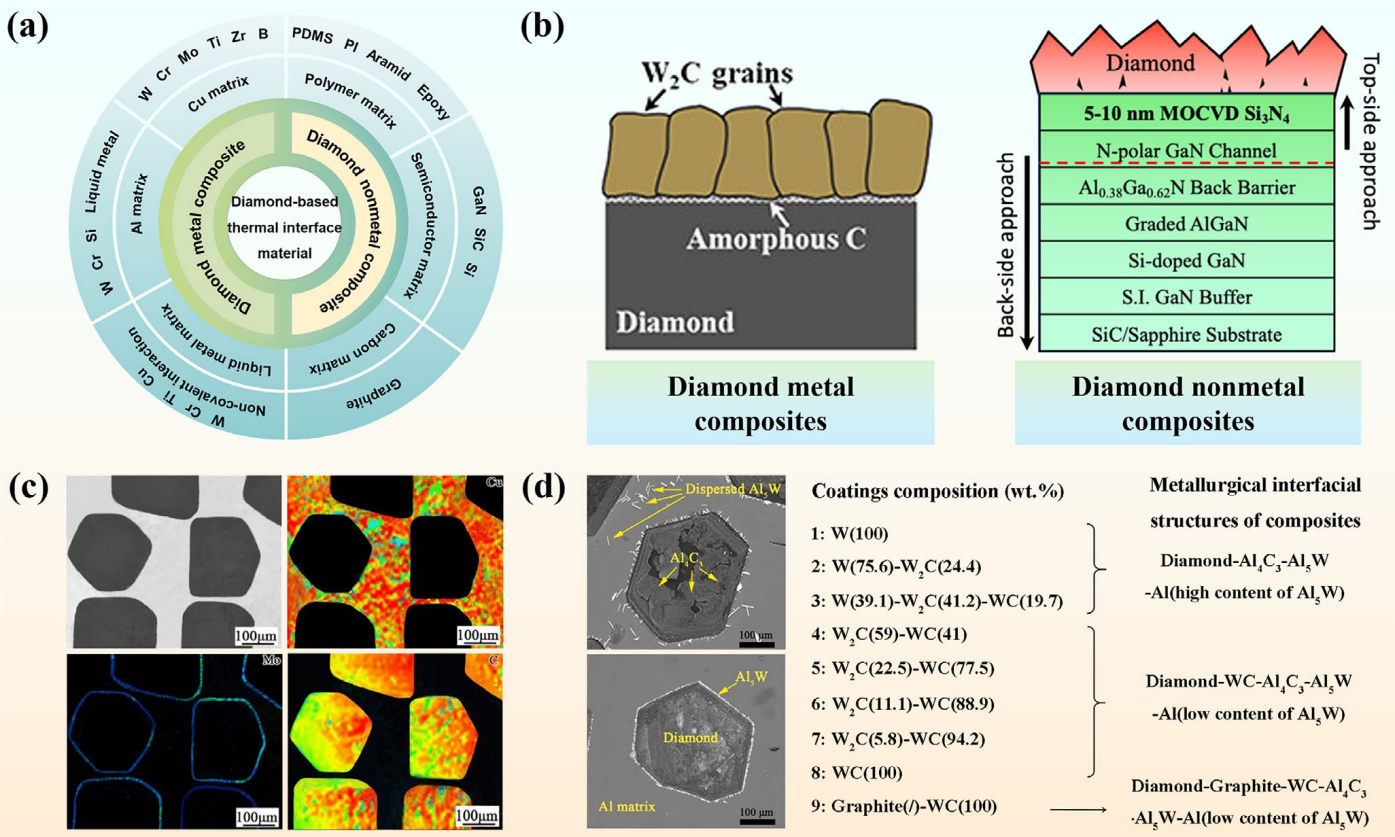
(a) The types, performance integration methods and (b) typical schematic diagram of diamond composite thermal interface material. Reproduced with permission. [139, 154]. Copyright 2022, American Chemical Society and Copyright 2021, American Chemical Society. (c) SEM image and EPMA elemental maps of Mo‐coated diamond/copper composites with Mo‐coated diamond. Reproduced with permission. [152]. Copyright 2022, Springer LLC.(d) SEM of diamond/Al interfacial coatings. Reproduced with permission. [155]. Copyright 2025, Elsevier B.V.

Ceramic fillers conduct heat predominantly through lattice vibrations while lacking free electrons, which endows them with excellent electrical insulation. This combination makes them attractive candidates for high‐thermal‐conductivity, electrically insulating polymer‐matrix composites. Among widely used inorganic non‐metallic fillers, such as BN, AlN, Al_2_O_3_ and SiC, diamond exhibits the highest intrinsic thermal conductivity, with BN‐based systems typically ranking second while retaining good insulation. Table 3 summarizes representative thermal and electrical properties of diamond‐ and BN‐based TIM systems as two benchmark filler families for advanced thermal management. Diamond possesses exceptional thermal properties, including the highest known isotropic thermal conductivity among bulk materials, approximately 2300 W·m^−1^·K^−1^, together with an extremely low CTE of 1 ppm·K^−1^ at room temperature [137]. These characteristics make diamond an ideal candidate for advanced thermal management applications. Diamond particle‐reinforced composites have recently gained significant attention as a new generation of TIMs, offering both high thermal conductivity and tunable CTE [138]. As illustrated in Figure 5a,b [138, 139], a variety of matrix materials and diamond‐based composite configurations have been explored for effective thermal management in electronic devices. In this chapter, we provide an overview of diamond‐metal and diamond‐nonmetal composites, highlighting their design strategies, interfacial engineering, and thermal performance.

**TABLE 3:** Main properties of boron nitride and diamond.

TIM	Thermal conductivity [W·m^−1^·K^−1^]	Bending strength [MPa]	Thermal expansion coefficient [10^−6^·K^−1^]	Dielectric constant	Dielectric strength [kV·mm^−1^]
BN	300–2000 [140]	32–95 [141]	1 [142]	2–5 [143]	150–1200 [144]
Diamond	2300 [145, 146, 147]	125 [148]	1–5 [148]	5.3–5.7 [149]	2000 [150]

Owing to its outstanding stability and isotropy, diamond is extensively employed as a thermally conductive filler whose volume fraction, particle size and spatial distribution can be tuned to tailor composite thermal properties, while surface metallization is concurrently implemented to mitigate the interfacial adhesion deficit arising from the large diamond‐matrix contact angle and thus boost interfacial heat transfer [151].

Diamond/Cu composites are considered promising heat sink materials for high‐power‐density electronics because they exhibit outstanding thermal conductivity, corrosion resistance, and endurance under extreme conditions. However, their practical application is hindered by high ITR between diamond particles and the Cu matrix, primarily caused by poor interfacial bonding, low‐conductivity interlayers, and severe phonon scattering. Li et al. [152] introduced a Mo coating on Chemical Vapor Deposition diamond via vacuum deposition, promoting carbide formation and wettability, yielding a composite whose effective thermal transport reaches 329 W·m^−1^·K^−1^ (Figure 5c). However, limited connectivity between dispersed diamond particles remains a challenge. Wei et al. [153] developed a continuous diamond skeleton (DS) structure coated tungsten (W) with a thickness of 300 nm, which reduced the contact angle to 13.2° and suppressed graphitization. The resulting DS/Cu composite achieved 575 W·m^−1^·K^−1^ at only 18.4 vol.% diamond. To further investigate interfacial thermal transport, Zhang et al. [139] fabricated Cu/interlayer/diamond sandwich films (W, interconnected W‐Tungsten carbide (W2C), and W2C) by sputtering and annealing. Time‐domain thermoreflectance measurements yielded interfacial conductance G = 29.4 MW·m^−1^·K^−1^ (Cu/W/diamond), 25.8 MW·m^−1^·K^−1^ (Cu/W‐W2C/diamond) and 19.9 MW·m^−1^·K^−1^ (Cu/W2C/diamond), with Diffuse Mismatch Model (DMM) and MD confirming that thinner, interconnected carbide layers optimize phonon transmission. Interfacial bonding plays a decisive role. The formation of semi‐coherent carbide interlayers (e.g., titanium carbide (TiC)/diamond‐Cu interface, molybdenum (Mo)/diamond‐Cu interface, W/diamond‐Cu interface, etc.) has been reported to effectively suppress phonon scattering and facilitate heat transport across the interface.

Diamond/Al‐matrix composites, combining elevated thermal conductivity with low density, offer significant potential for thermal management in lightweight vehicles and aerospace engineering. Its thermal performance is greatly influenced by the manufacturing process. Solid‐phase techniques, though structurally stable, often result in poor interfacial bonding and limited diamond content. To address this, Li et al. [156] engineered in situ aluminum carbide (Al_4_C_3_) at the diamond/Al interface to study TC stability under 200 thermal cycles (218–423 K). Discrete Al_4_C_3_ “pinning” phases formed robust interfaces that limited TC degradation to only 2–5%, with a 272 µm‐diamond/Al‐matrix composite retaining >700 W·m^−1^·K^−1^ after cycling.

In contrast, liquid‐phase techniques allow for higher thermal conductivity by enabling better infiltration and diamond dispersion. However, the researchers often promote the formation of unstable Al_4_C_3_ phases, which compromise moisture resistance. To mitigate this issue, strategies such as matrix alloying and surface coating of diamond particles (e.g., with W, Ti, or SiC) have been explored to enhance both interfacial stability and thermal transport. For instance, Yang et al. [155] explored interfacial reactions in W‐coated diamond/Al composites, aiming for high thermal conductivity capability and moisture resistance (Figure 5d). The thermal conductivity of the diamond /WC/Al_4_C_3_/Tungsten aluminum (Al_5_W)/Al interface structure is 658 W·m^−1^·K^−1^, and it only decreases by 8.9% after water treatment at 680 h. The WC/Al_4_C_3_/Al_5_W intermediate layer effectively inhibits the hydrolysis of Al_4_C_3_, ensuring stable thermal performance.

Liquid metal has a notable thermal conductivity (15–39 W·m^−1^·K^−1^) and excellent fluidity, yet its inherently elevated surface tension frequently causes leakage, posing significant challenges for reliable integration into electronic device packaging. Wei et al. [157] enhanced the thermal conductivity of Bi‐In‐Sn (BIS) alloys by incorporating Cr_3_C_2_‐coated diamond particles via sintering. the carbide coatings improved interfacial bonding and reduced voids, respectively. Although the functional interlayers enhanced the phonon energy transfer efficiency at the diamond‐metal matrix interface, thereby improving thermal conductivity, excessive coating thickness and interfacial voids were found to degrade thermal performance. Furthermore, the use of single‐sized diamond particles limited the formation of continuous thermal conduction pathways in the composite. To overcome these limitations and further improve thermal transport in liquid metal systems, Lin et al. [158] introduced chromium (Cr)/Cu‐coated diamond particles with a bimodal size distribution into EGaInSn to fabricate liquid metal composites. The cooling efficiency of this composite material is approximately 1.9 times higher than that of commercial liquid metals, and it demonstrates outstanding heat dissipation performance in high‐power LED devices, providing a promising approach for advanced thermal management applications. Voids and porosity in liquid metals can significantly hinder thermal transport; the addition of diamond reduces void formation by improving particle packing and modifying solidification behavior, thereby decreasing thermal scattering and enhancing overall conductivity. Experimental and numerical studies show that such voids arise from incomplete wetting and incomplete infiltration during solidification and that their presence and volume fraction correlate inversely with bulk thermal conductivity. Coating or metallizing the diamond surface (e.g., Titanium (Ti), TiC, Cr_3_C_2_) improves wetting and reduces interfacial voids, leading to enhanced ITC and significantly higher composite thermal conductivity [157].

While filler selection and composite formulation establish the basic thermal potential of adhesive‐based TIMs, the ultimate heat‐dissipation performance is strongly governed by how efficiently heat carriers traverse filler–matrix and filler–filler interfaces and how continuous thermal pathways are constructed across multiple length scales. Therefore, beyond material composition, interfacial regulation and structural organization become decisive factors in determining effective thermal transport in composite TIMs, which are discussed in the following section.

### 3.2 Interfacial Engineering and Thermal‐Pathway Construction in Composite TIMs

Diamond‐metal composite systems have long been explored for high thermal conductivity applications due to the excellent intrinsic conductivity of diamond and the continuous heat‐transfer pathways provided by metallic matrices such as Cu, Al, and silver (Ag). However, the practical performance of these composites is severely restricted by two major factors: (i) poor interfacial wetting between diamond and metal, which leads to high ITR and weak mechanical bonding, and (ii) the high density of metal matrices, which limits their application in lightweight or flexible thermal management systems. Various surface modification strategies, including carbide‐forming interlayers (e.g., Ti, Cr, W) and alloying, have been developed to enhance interfacial adhesion and reduce phonon scattering, but these approaches often involve complex processing and can compromise the chemical stability of diamond. Consequently, recent research has shifted toward non‐metallic matrices such as polymers and ceramics, offering improved processability, lower density, and the potential to achieve high thermal conductivity through interface engineering and filler alignment.

**FIGURE 6:**
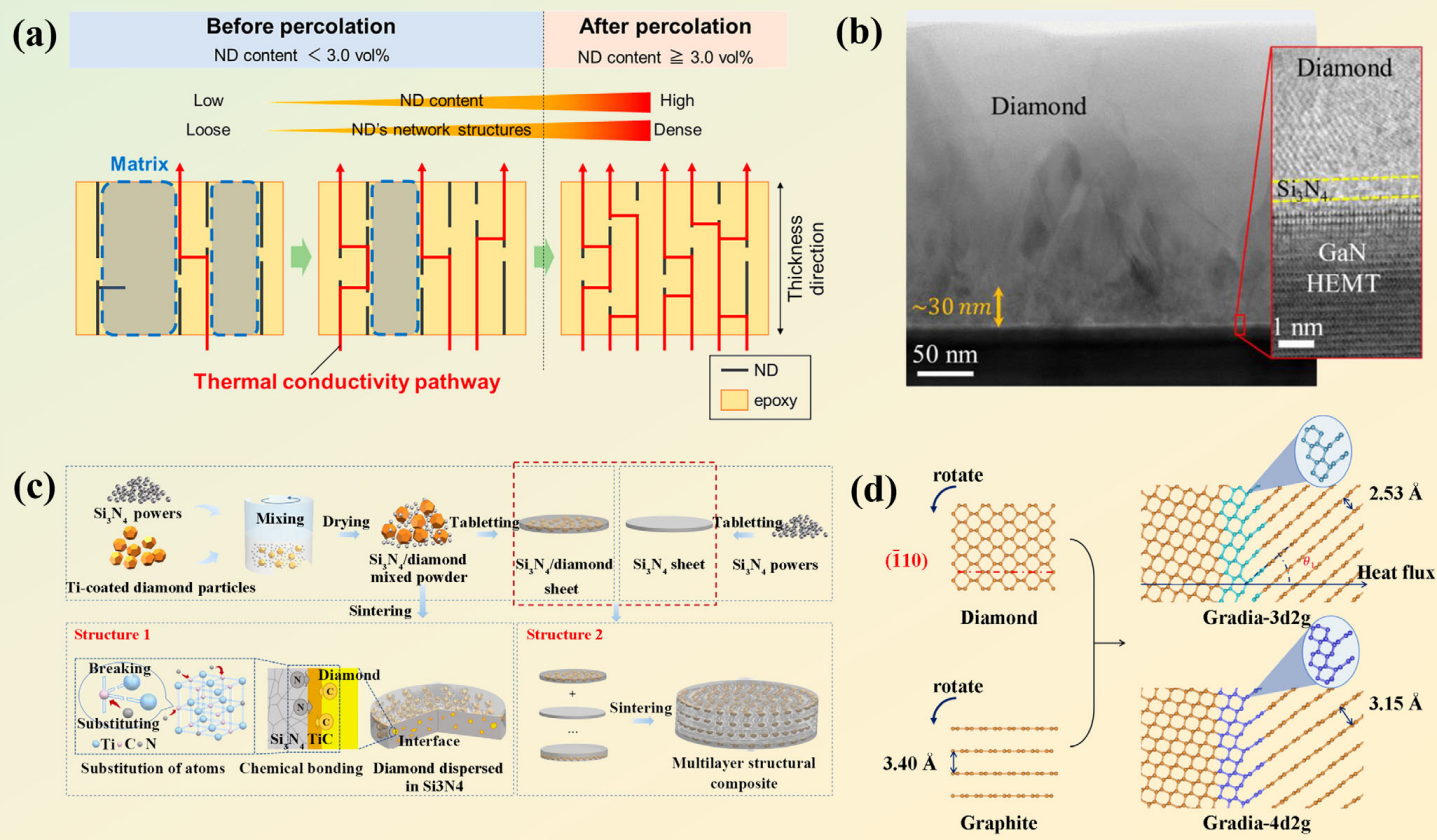
(a) Epoxy/nanodiamond through‐thickness percolation mechanism. Reproduced with permission. [161]. Copyright 2023, American Chemical Society. (b) Microstructure of Si_3_N_4_. Reproduced with permission. [154]. Copyright 2021, American Chemical Society. (c) Fabrication and structural design schematic. Reproduced with permission. [162]. Copyright 2022, Elsevier Ltd. (d) Schematic diagrams of the two Gradia structures. Reproduced with permission. [167]. Copyright 2024, American Chemical Society.

Polymers have been widely used in thermal management materials, and the introduction of diamond fillers can significantly enhance their heat transfer performance. Wang et al. [159] combines numerical simulation and experiments to clarify how diamond filler morphology modulates the through‐plane heat transport of silicone‐ based TIMs. Results showed that lamellar and rod‐like diamonds particles more readily form continuous thermal pathways, achieving a maximum conductivity of 1.357 W·m^−1^·K^−1^ at 80 wt.% loading, outperforming conventional Al_2_O_3_ fillers and highlighting the critical role of filler shape in polymer composite design. Zhang et al. [160] further reported an epoxy composite with 43.2 wt.% surface‐modified diamond fillers, reaching a through‐plane thermal conductivity of 22.7 W·m^−1^·K^−1^. However, such high filler loadings inevitably increase weight, brittleness and processing costs. As demonstrated in Figure 6a, ice‐templating alignment has been used to construct efficient, vertically oriented heat‐conduction networks at relatively low filler contents. Nanodiamond‐epoxy composites achieved a through‐plane conductivities about 16 times that of neat epoxy at only 4.6 vol.% loading, while silicone‐rubber composites with 80 wt.% single‐crystal diamond reached 1.357 W·m^−1^·K^−1^ [161]. These results highlight that filler shape, orientation and percolation behavior together govern the thermal performance of flexible polymer matrices.

Chowdhury et al. embedded polycrystalline diamond within 1 nm of the GaN channel while suppressing residual stress, thereby achieving an interfacial thermal boundary resistance of about 3.1±0.7 m^2^K GW^−1^ at the diamond/Si_3_N_4_/GaN interface (Figure 6b) [154]. The diamond layer exhibited nearly isotropic growth with a thermal conductivity of 638 ± 48 W·m^−1^·K^−1^. In contrast. In contrast, Wang et al. [162] developed a semiconductor‐based composite using Ti‐coated diamond particles embedded in a Si_3_N_4_ matrix (Figure 6c). During processing, a TiC_i_N_1‐i_ interfacial layer formed, which strengthened interfacial bonding, inhibited diamond graphitization and significantly improved heat transfer across the diamond/ceramic interface. A multilayer structural design further introduced anisotropic thermal conductivity. At a diamond content of 50 vol.%, the composite achieved a peak thermal conductance of 201.96 W·m^−1^·K^−1^, corresponding to a 272.87% increase relative to monolithic Si_3_N_4_. This work presents an effective strategy for interfacial engineering and directional heat conduction in semiconductor/diamond composites.

Beyond optimizing bulk transport in the constituent phases, high‐performance power electronic devices also require efficient heat transfer at material interfaces. ITC is well recognized to be influenced by bonding strength and phonon vibrational mismatch [163]. The relatively small lattice mismatch between diamond and graphene elevates the saturation speed of graphene [164], reduces contact resistance, and increases infrared noise at a certain degree. However, most graphene‐diamond composites are joined through van der Waals interactions, which limit phonon transmission and hinder effective interfacial heat transport [165]. Gradia, a graphite‐diamond hybrid material, combines the advantages of both phases and bridges the gap in their mechanical and electronic properties [166]. As shown in Figure 6d, Yang et al. [167] constructed a Gradia structure comprising interpenetrating diamond and graphite nanodomains with exceptionally high interfacial thermal conductivity, capable of efficiently dissipating heat generated within the graphite regions. The engineered graphite‐diamond hybrid structures demonstrated exceptional ITC, with Gradia‐3d2g reaching Gradia‐4d2g 5980.36 and 5418.85 MW·m^−2^·K^−1^, respectively. Time‐domain thermoreflectance (TDTR) and cross sectional TEM measurements revealed that covalent bonding at the graphite‐diamond interface enables highly efficient phonon transmission.

In diamond‐nonmetal systems, particularly diamond‐polymer composites, the mismatch in surface energy, phonon spectra, and chemical polarity between diamond and polymers leads to poor interfacial wetting and high ITR. Interfacial engineering, including diamond surface functionalization (─OH, ─COOH, ─NH_2_, or silane groups) and the introduction of molecular bridges such as polydopamine, effectively enhances interfacial compatibility and phonon coupling. Moreover, processing techniques like hot pressing, solution casting, and electrospinning facilitate filler alignment and the formation of continuous thermal pathways. These strategies collectively mitigate interfacial resistance and enable diamond‐polymer composites to achieve superior thermal conductivity and mechanical flexibility.

With the rising demand for efficient thermal management in electronics and energy systems, incorporating 2D fillers into polymer matrices has become an effective strategy for enhancing composite thermal conductivity. Highly conductive 2D materials such as graphene [168], BN, and MXenes [169, 170] can form continuous heat‐conduction pathways, significantly improving overall heat dissipation performance.

**FIGURE 7:**
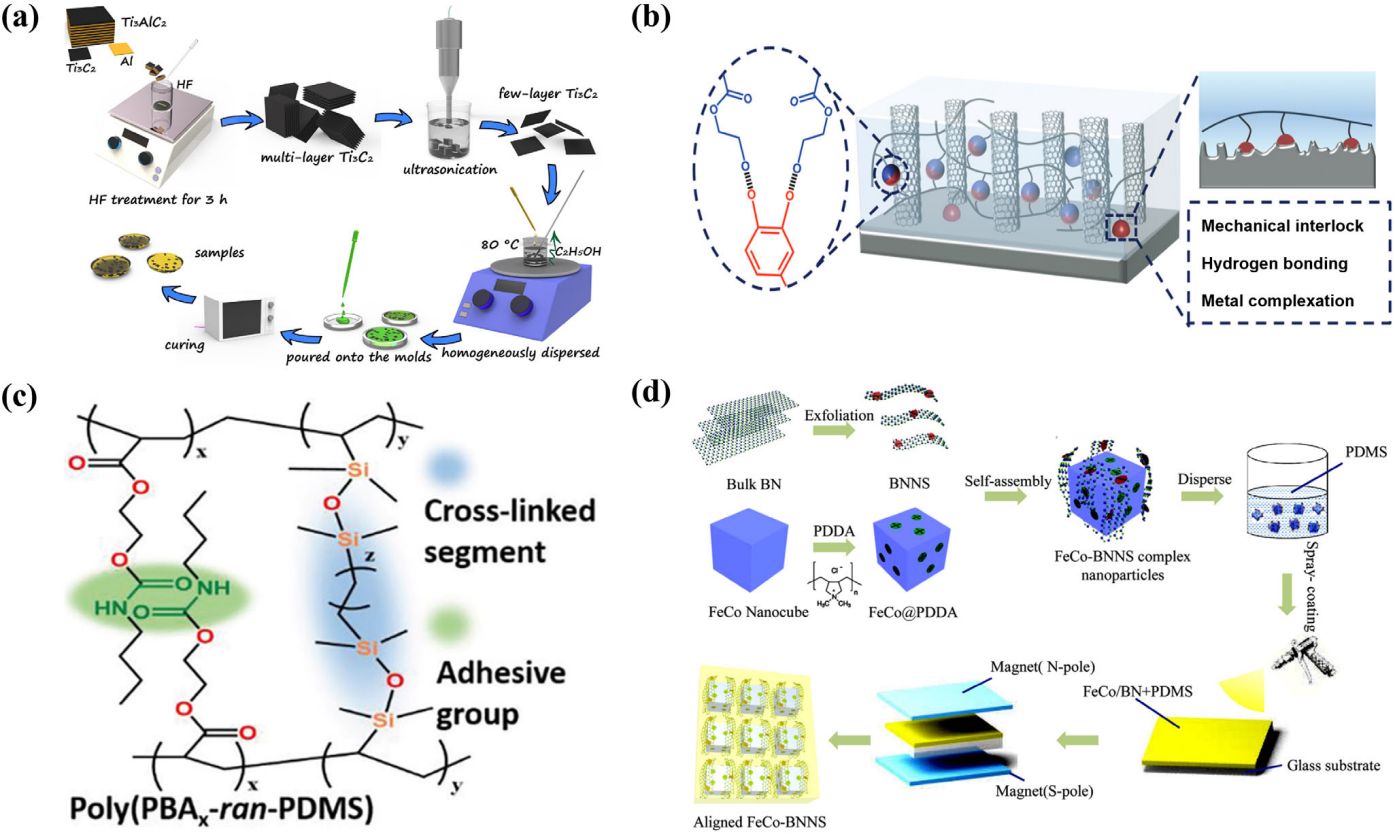
(a) Preparation process diagram of Ti_3_C_2_/ epoxy resin. Reproduced with permission. [170]. Copyright 2019, Springer Nature. (b) P(DMA‐HEMA)/VACNT adhesion mechanism. Reproduced with permission.[171]. Copyright 2022, Wiley‐VCH GmbH. (c) Molecular structures of poly (PBAx‐ran‐PDMS) block copolymers. Reproduced with permission. [171]. Copyright 2022, Wiley‐VCH GmbH. (d) Schematic diagram of fecO‐BNNs formation mechanism and FeCo/hBN arrangement. Reproduced with permission. [173]. Copyright 2019, American Chemical Society.

A representative study by Kang et al. demonstrated the effective use of MXenes as thermally conductive fillers in epoxy composite [170] (Figure 7a). After vacuum filtratio followed by HP, Ti_3_C_2_T_X_ MXenes were aligned within the epoxy matrix, forming continuous and efficient thermal pathways. At a loading of 2.25 vol.%, the composite reached a thermal conductivity of 3.89 W·m^−1^·K^−1^, more than 12 times that of neat epoxy. This performance is enhanced by the high thermal conductivity of MXenes and their ability to form a well‐connected thermal network with minimal ITR. This enhancement stems from the intrinsic high thermal conductivity of MXenes and the formation of a well‐connected lamellar network with low interfacial resistance, while the composite also retained good flexibility and processability.

Beyond optimizing fillers, engineering the polymer matrix—chemically or structurally—offers another route to improve dispersion, interfacial coupling, and heat transport. Zhang et al. [168] designed a mussel‐ and snail‐inspired copolymer, poly(dopamine methacrylate‐co‐hydroxyethyl methacrylate (P(DMA‐HEMA)), and obtain a composite exhibiting both strong adhesion and high anisotropic thermal conductivity (Figure 7b). Through multiple interactions—including hydrogen bonding and metal coordination—with both the substrate and VACNTs, the copolymer improves interfacial contact and reduces thermal resistance, enabling performance superior to many conventional thermal adhesives even without external pressure.

Complementarily, Yu et al. [171] incorporated folded graphene structures into an adhesive poly‐2‐[[(butylamino) carbonyl]oxy]ethyl ester and polydimethylsiloxane, vinyl‐end‐terminated polydimethylsiloxane (poly(PBAx‐ran‐PDMS)) matrix using a template‐assisted stretching‐curing process (Figure 7c). This method induces vertically aligned graphene channels within the elastomer, greatly enhancing through‐plane heat transfer. The composite maintains mechanical flexibility and adhesion while achieving a vertical thermal conductivity of 13.0 W·m^−1^·K^−1^ and an ultra‐low contact thermal resistance of 9.4 K·mm^2^·W^−1^, demonstrating how structural engineering can yield high‐performance TIMs without compromising softness or processability. The synergy of these scale specific strategies show that thermal transport in soft composites can be leap frogged by orchestrating order at disparate length scales and charts a route to adhesive thermal materials that are simultaneously compliant processable and highly efficient.

Applying external fields (magnetic, electric, or mechanical) during composite preparation aligns 2D fillers directionally, creating oriented pathways for heat conduction. This method notably improves anisotropic thermal conductivity and enhances heat dissipation efficiency in specific directions. Among various external fields, magnetic field assistance has shown notable and consistent advancements in aligning fillers for improved thermal management. Initially, Lin et al. [172] demonstrated that hexagonal boron nitride (hBN) platelets modified with iron oxide nanoparticles could be effectively aligned under a magnetic field of 400 mT.

Subsequent research built upon these initial findings by optimizing both filler materials and alignment methodologies to further enhance thermal performance. Yuan et al. [173] advanced the technique by employing FeCo nanocubes exhibiting defined {100} crystal facets, which exhibited strong and uniform interactions with exfoliated hBN nanosheets (BNNs), as demonstrated in Figure 7d. A reduced magnetic field strength of about 35 mT facilitated the vertical alignment of these composite structures, forming highly efficient heat conduction channels. Consequently, polymer films incorporating 50 wt.% BN filler reached a thermal conductivity of 2.25 W·m^−1^·K^−1^, a sevenfold increase compared to randomly dispersed fillers (0.325 W·m^−1^·K^−1^). This approach overcame previous limitations concerning particle size uniformity and alignment consistency, marking significant progress in thermal management efficiency.

Despite these advancements, achieving uniform multidirectional thermal conduction remained a critical challenge due to inherently unidirectional filler alignment. Recent innovations have thus shifted toward the development of 3D filler architectures to enhance thermal conduction comprehensively. Jiang et al. [174] introduced a sophisticated 3D bridging structure combining horizontally aligned, magnetically modified BN nanosheets (M@BNNS) under moderate magnetic fields (30–50 mT), with randomly dispersed, non‐magnetic organo‐grafted BN nanosheets. This hybrid filler strategy enabled the formation of continuous multidirectional thermal conduction pathways. At relatively moderate filler loadings, the composite exhibited exceptional through‐plane thermal conductivity of 2.88 W·m^−1^·K^−1^, representing a 194.2% improvement over single‐orientation composites. Moreover, this structure notably improved flame retardancy, offering a dual‐functional composite suitable for advanced electronic packaging applications, thereby addressing both thermal management and safety requirements simultaneously.

In addition to magnetic‐field‐assisted alignment, electric‐field‐assisted strategies have recently emerged as powerful tools for constructing anisotropic thermal‐conduction pathways in polymer‐based composites. Xu et al. [175] reports an alcohol‐based Plasma Enhanced Chemical Vapor Deposition system under a vertical electric field of about 30 V cm^−1^, in which vertical graphene (VG) arrays with a height of 18.7 µm were grown. The resulting TIMs achieved a through‐plane thermal conductivity of 53.5 W·m^−1^·K^−1^ and contact thermal resistance of 11.8 K·mm^2^·W^−1^.

Another illustrative example concerns high‐frequency electric‐field‐assisted preparation of BN/Epoxy resin composites, where hBN nanosheets were dispersed in epoxy and oriented by an alternating electric field with a frequency of 10 kHz and a field strength up to about 30 V mm^−1^ [176]. This induced orientation effectively suppressed filler agglomeration and significantly enhanced the composite thermal conductivity.

These examples highlight two electric‐field routes: in situ growth of vertically aligned filler frameworks, as in vertical graphene arrays, and post‐dispersion orientation of 2D fillers in a polymer matrix, as in h‐BN/epoxy systems. Compared with magnetic‐field methods that rely on magnetic susceptibility or magnetic‐particle decoration, electric‐field approaches control filler orientation via dielectrophoretic and Coulomb forces and can be integrated into curing or deposition processes, providing a complementary route for engineering 3D thermal‐conduction pathways.

## 4 Multi‐Scale Simulation for Power Electronics Packaging

In this chapter, the content explores the simulation methods and key mechanisms for power electronics packaging materials and thermal management structures within a multi scale framework, covering approaches at the quantum and atomic, mesoscopic and macroscopic scales. The emphasis is on how these techniques are applied to analyze heat transport, microstructure evolution and thermo mechanical response in materials and interfaces that are critical to high power modules, rather than on an exhaustive survey of all packaging processes. At the atomic scale, MD simulations are employed to resolve atomic motion and local structural rearrangements during thermally activated processes such as sintering, interfacial bonding and phase transformation. At the mesoscopic scale, phase field models capture the evolution of microstructures and interfaces between fillers and matrices and link morphological features to effective thermal and mechanical properties. At the macroscopic scale, finite element based multi physics simulations describe the behavior of packaging assemblies under coupled thermal, mechanical and electrical loading conditions representative of service environments. Together, this chapter provides an outline and roadmap for using multi scale simulation to clarify key heat transfer mechanisms and to guide the design and optimization of thermal management materials and structures in power electronics.

### 4.1 Interfacial Thermal Transport Mechanisms

In polymer‐based composites and TIMs, understanding the fundamental physical mechanisms governing interfacial heat transport is essential for optimizing thermal performance. Heat is predominantly carried by phonons in these systems, while electrons can contribute to energy transport at metallic fillers or metal‐insulator interfaces, particularly in high‐conductivity metals or metallized interfaces. ITR and filler‐filler contact resistance are often the primary bottlenecks limiting the effective thermal conductivity of composites, and their magnitude is determined by a combination of intrinsic material properties and interfacial characteristics [177, 178, 179]. Phonon scattering at interfaces is a major source of thermal resistance. Differences in vibrational spectra, acoustic impedance mismatch, surface roughness, defects, and chemical heterogeneity cause phonon reflection or scattering, reducing the mean free path of heat carriers and thereby hindering heat flux [180, 181, 182]. Acoustic and diffuse mismatch mechanisms, as described by the Acoustic Mismatch Model (AMM) and DMM, provide classical frameworks for understanding ITR: AMM assumes perfectly smooth interfaces and elastic collisions where phonon transmission depends on incidence angle and mode matching, while DMM considers fully randomized scattering, with isotropic phonon transmission and reflection probabilities. However, real interfaces often exhibit partially coherent or quasi‐elastic phonon transport, along with complex effects such as vibrational mode coupling, interfacial bond reconstruction, and localized states, which are not captured by classical models, frequently leading to underestimation of actual ITR. Interfacial chemical bonding directly affects thermal transport efficiency [183, 184]. Strong covalent or chemical bonds can enhance phonon mode coupling and reduce scattering, forming continuous heat conduction pathways, whereas weak van der Waals interactions or physical adsorption tend to create thermal bottlenecks. Furthermore, the overlap of phonon density of states (PDOS) between adjacent materials is a critical indicator of ITC: higher PDOS matching allows more efficient energy transfer for both low‐ and high‐frequency phonons [185]. At metal‐insulator interfaces, electron‐phonon coupling introduces an additional channel for heat transfer, which is particularly significant at elevated temperatures or in composites containing metallic fillers [186]. Structural factors such as interface morphology, nanoscale roughness, filler dispersion, and polymer chain entanglement further modulate phonon transport, leading to nonlinear, temperature‐dependent, and anisotropic thermal behavior [187]. Modern computational approaches, including density functional theory (DFT) and MD simulations, have been extensively employed to elucidate the atomic‐ and electronic‐scale origins of ITR [188]. DFT can reveal interfacial phonon modes, localized states, electron density redistribution, and bonding characteristics that govern heat flow, while MD simulations quantitatively evaluate the influence of interface roughness, stress, temperature gradients, and polymer chain rearrangement on phonon scattering. The complementary use of these methods enables a comprehensive understanding of ITR, providing a scientific basis for rational design strategies‐such as filler selection, interface chemical modification, and nanoscale structural optimization‐to enhance the thermal performance of composite materials.

Packaging materials for thermal management are typically composed of an organic polymer matrix embedded with high thermal conductivity inorganic fillers. Given the inherently low thermal conductivity of polymers, the incorporation of thermally conductive fillers aims to establish continuous heat conduction pathways within the composite material [189]. These fillers form interconnected networks inside the matrix, serving as the primary channels for heat transfer from high to low temperature regions. The overall thermal conductivity of PMs is not only determined by the intrinsic properties of the polymer and filler but is also significantly constrained by the ITR between the polymer and the filler, as well as the contact thermal resistance between adjacent filler particles. The heat transport behavior of these filler‐loaded polymer composites can be interpreted through percolation theory. At low filler concentrations, the fillers are isolated and contribute minimally to thermal transport. As the filler content increases, physical contact among fillers becomes more frequent, leading to the formation of localized thermal chains and eventually a continuous thermal network. Recent studies indicate that interfacial phonon spectrum matching and chemical bonding strength play decisive roles in establishing effective heat pathways, with phonon scattering control and mode coupling being key factors for enhancing thermal conductivity. This enables efficient heat transfer along the filler network, thereby largely enhancing the composite's overall thermal conductivity. Commonly used fillers include metallic particles (Ag, Cu, Al) [190], inorganic oxides or nitrides (e.g., Al_2_O_3_, ZnO, BN, SiC) [191], and carbon‐based materials such as graphene, carbon nanotubes, and diamond [192]. Among these, graphene stands out due to its exceptionally high intrinsic thermal conductivity, 2D structure, and large specific surface area, allowing it to significantly improve thermal performance even at low loading fractions. However, relatively high intrinsic thermal conductivity of fillers does not necessarily guarantee a corresponding enhancement in composite thermal conductivity. ITR often emerges as the bottleneck. Differences in the vibrational (phononic) or electronic properties of dissimilar materials lead to partial reflection and scattering of energy carriers‐such as phonons or electrons‐at the interface. Particularly at organic‐inorganic interfaces, phonon frequency matching and interfacial chemical bonding determine heat transfer efficiency. First‐principles calculations, exemplified by DFT, provide a mechanistic link between chemistry, microstructure, and interfacial heat transport central to this review. DFT evaluates the stability of interlayers and surface terminations, maps charge redistribution and bonding strength, and predicts phonon dispersion that underpins trends in thermal boundary conductance. For ceramic substrates and TIMs, these insights guide the choice of metallization on Si_3_N_4_ and the tuning of diamond or hBN surface chemistry, while identifying defect motifs that degrade conductivity or reliability. Recent studies using DFT have revealed the contributions of phonon spectrum alignment and interfacial states coupling to ITR, while MD simulations provide insights into how interface roughness, atomic rearrangement, and polymer chain entanglement affect phonon scattering. This discontinuity disrupts the propagation paths of heat carriers, reduces their mean free paths, and consequently diminishes the efficiency of thermal energy transport. Such interfacial effects are among the primary reasons why the thermal conductivity of composites typically deviates from ideal, linear enhancement models.

**FIGURE 8:**
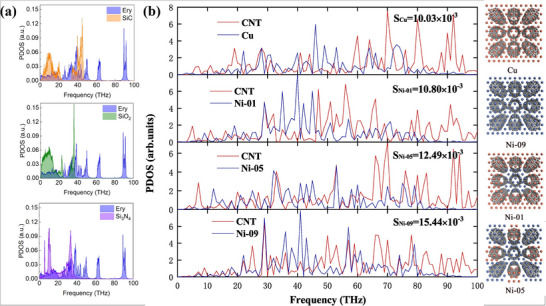
(a) PDOS of Ery@SiC, Ery@SiO_2_, and Ery@Si_3_N_4_. Reproduced with permission. [198]. Copyright 2025, Youke Publishing Co.,Ltd. (b) PDOS of the Cu‐Ni‐VACNT system with different contact areas (Reference Cu‐VACNT system, Cu‐Ni‐01‐VACNT system, Cu‐Ni‐05‐VACNT system, and Cu‐Ni‐09‐VACNT system) for Ni atoms. Reproduced with permission. [199]. Copyright 2024, Elsevier B.V.

Inorganic fillers generally possess ordered lattice structures that facilitate directional phonon propagation, whereas polymers, due to their disordered molecular chains and substantial chain entanglement, tend to induce frequent phonon scattering, thereby impeding heat transport and reducing thermal performance [193]. Accordingly, minimizing phonon scattering and enhancing interfacial adhesion are crucial strategies for improving ITC. From a theoretical standpoint, classical models such as the AMM and the DMM provide fundamental frameworks for estimating ITR. However, AMM and DMM assume ideal smooth interfaces without inelastic scattering, and thus cannot capture vibrational mode hybridization, interfacial bond reconstruction, or phonon coherence effects at real heterogeneous interfaces. At the nanoscale, interfacial heat transfer often exhibits partially coherent or quasi‐elastic behaviors, which have emerged as a frontier research topic. These limitations explain why classical models frequently underestimate the complexity of phonon scattering at practical interfaces [194]. In recent years, MD simulations have become widely adopted to investigate the thermal transport behavior at organic‐inorganic interfaces. These studies have revealed that interfacial adhesion plays a significant role in modulating ITR [195]. Furthermore, Lu et al. [196] introduced the concept of electron‐phonon coupling at metal‐insulator interfaces, highlighting an additional thermal transport channel and thereby broadening the scope of interfacial thermal transport mechanisms. Current research also explores interfacial thermal transport through analyses of PDOS, matching and interatomic interactions at the interface, offering deeper insight into the fundamental mechanisms governing thermal conduction in composite materials. The phonon overlap energy, derived from the projected density of states, serves as a quantitative indicator of the vibrational compatibility between different materials. A higher overlap energy implies better phonon mode alignment, thereby promoting more efficient interfacial thermal transport [197]. In a MD study conducted by Li et al. [198], the interfacial heat transfer behavior between erythritol and various nanoparticles‐including crystalline SiC and Si_3_N_4_, as well as amorphous SiO_2_, was investigated. Analysis of their PDOS revealed that both SiC and Si_3_N_4_ possess cutoff frequencies around 50 THz but with relatively modest peak intensities, whereas SiO_2_ exhibited a lower cutoff frequency near 40 THz accompanied by a more pronounced peak, as shown in Figure 8a. These findings suggest that SiC and Si_3_N_4_ offer a broader vibrational spectrum for coupling with erythritol phonons, which facilitate better vibrational resonance at the interface compared to SiO_2_. Wang et al. [199] introduced an innovative approach to enhance the ITC between vertically aligned carbon nanotube (VACNT) arrays and Cu substrates. By incorporating various metal elements (Ag, Al, Ti, and Ni), they achieved improved ITC at the Cu‐VACNT interface, primarily attributed to the strengthened interfacial adhesion. These findings indicate that by tuning the type of interfacial chemical bonding and the contact area of metal atoms, one can selectively optimize phonon transport across different frequency ranges, enabling interface phonon engineering. While Ag and Al interacted with VACNTs mainly via physical adsorption, Ni and Ti formed chemical bonds with the nanotubes, leading to significantly stronger interfacial coupling. Furthermore, the PDOS alignment at the Ni‐VACNT interface was found to improve as the contact area between Ni atoms and the Cu substrate increased, as shown in Figure 8b. However, in the case of Ti, the interfacial bonding with VACNTs became excessively strong, which adversely affected the coupling of high‐frequency phonon modes. These insights offer important guidelines for the rational design and material selection aimed at optimizing thermal transport across interfaces in composite electronic packaging systems.

It is worth noting that recent studies on interfacial thermal transport have shifted from empirical models to theoretical descriptions based on atomic and electronic structures. DFT reveal interfacial phonon coupling and bonding characteristics by analyzing electronic band structures and phonon dispersion, while MD enables quantitative evaluation of interfacial heat transfer under practical conditions involving temperature, stress, and structural roughness. Therefore, DFT is suitable for understanding the origin of interfacial phonon modes and chemically bonded interfaces, whereas MD is more applicable to examining stress effects, temperature dependence, and nanoscale morphology.

Various strategies have been developed to enhance interfacial thermal transport, among which a typical approach involves the insertion of intermediate thermally conductive interfacial layers, known as the “thermal bonding” strategy. This method aims to strengthen interatomic/molecular interactions at the interface by enhancing the coupling between electrons and phonons, thereby improving heat carrier transmission capabilities and overall thermal conductivity. A comprehensive review of this strategy can be found in Ref. [200]. This article focuses on several other categories of interfacial thermal enhancement techniques, including surface modification, structural synergistic optimization, thermal filler design and doping modulation, as well as interface morphology engineering.

In polymer‐based composites, interfacial compatibility between fillers and the matrix is critical for determining thermal conductivity (TC). Strong phonon scattering at the interface can significantly impede heat transfer. thus, improving interfacial interactions is recognized as an effective way to reduce ITR and enhance overall TC [201]. For instance, Jiang et al. [202] chemically modified BN to enhance interfacial bonding with epoxy resin (EP), achieving a composite thermal conductivity 5.05 times higher than that of pure EP. Similarly, Liu et al. [203] employed binary transition metal oxides to modify BN, resulting in a substantial TC improvement even at very low filler loadings. Constructing continuous thermal networks is another key strategy to enhance thermal transport. Shen et al. [204] guided the alignment of BN nanosheets (BNNS) using self‐entangled cellulose fibers to form oriented thermal pathways. Ji et al. [205] adopted a liquid‐bridging strategy to strengthen the adhesion between thermoplastic polyurethane (TPU) and BN, while introducing 3D porous BN skeletons was also shown to effectively enhance TC [206] Additionally, Yang et al. [207] demonstrated that surface‐modified Al_2_O_3_ facilitated the construction of continuous heat pathways, and Bakalakos et al. [208], via finite element modeling, confirmed the positive influence of carbon nanotube (CNT) networks on composite thermal performance.

Structural synergistic optimization aims to regulate the coupling between nanostructures, thereby reducing phonon scattering and improving interfacial energy transfer efficiency. In multi‐walled carbon nanotube (MWCNT) networks, phonon scattering mainly arises from surface defects and extended transport paths [209]. Resonant coupling has emerged as an effective strategy to mitigate weak interfacial interactions by exciting additional heat carriers and enhancing thermal penetration. Its mechanisms involve hybridized phonon modes, modal interference, and phonon localization effects [210, 211]. This synergistic effect also manifests in the construction of multi‐scale thermal pathways through the hybridization of multiple fillers. Graphene nanoplatelets (GNPs), for example, improve the dispersion of CNT networks, suppress nanotube aggregation, and act as additional thermal bridges, significantly enhancing both TC and mechanical strength [212]. However, excessive filler content can lead to agglomeration, which may negate the benefits of GNPs [213]. Due to their large specific surface area and relatively low ITR, GNPs can facilitate a more uniform heat flux distribution in the matrix, further promoting interfacial thermal transport [214]. Lin et al. [215] coated vertically aligned CNT arrays with BN nanotubes (BNNTs), resulting in around 90% improvement in TC. This enhancement was attributed to the additional thermal channels provided by BNNTs and the suppression of interfacial phonon scattering.

Incorporating thermally conductive fillers into polymer matrices is one of the most direct and effective strategies to improve TC. Key factors influencing thermal transport include the intrinsic thermal conductivities of the matrix and fillers, filler volume fraction, filler size, morphology, distribution, and interfacial bonding strength [216]. Compared with spherical or cylindrical fillers, sheet‐like structures offer larger contact areas and denser filler‐filler interfaces, leading to lower ITR [217]. Additionally, high aspect‐ratio 1D or 2D fillers can establish effective thermal networks at low loading levels, reducing the percolation threshold and significantly enhancing TC [218]. Thermal fillers can be categorized by their structural dimensionality into 0D (spherical nanoparticles), 1D (e.g., CNTs, Ag nanowires), 2D (e.g., graphene, BN sheets), and 3D (e.g., 3D network architectures) [219]. Among them, 2D fillers‐especially layered structures such as graphite and hexagonal BN (hBN)‐demonstrate excellent interfacial compatibility and network stability, effectively reducing ITR between fillers and with the matrix, suppressing agglomeration, and offering anisotropic thermal transport properties [220]. Filler size is also critical, smaller fillers increase interfacial contact area but may introduce interfacial scattering, while larger fillers help construct stable heat pathways and reduce ITR [221]. Hybridizing fillers of different sizes, dimensionalities, and types has become a research hotspot. Through multiscale cooperative design, it is possible to construct continuous thermal pathways while optimizing microstructural distribution and filler orientation during processing, achieving a balance between thermal performance and processability [222].

**FIGURE 9:**
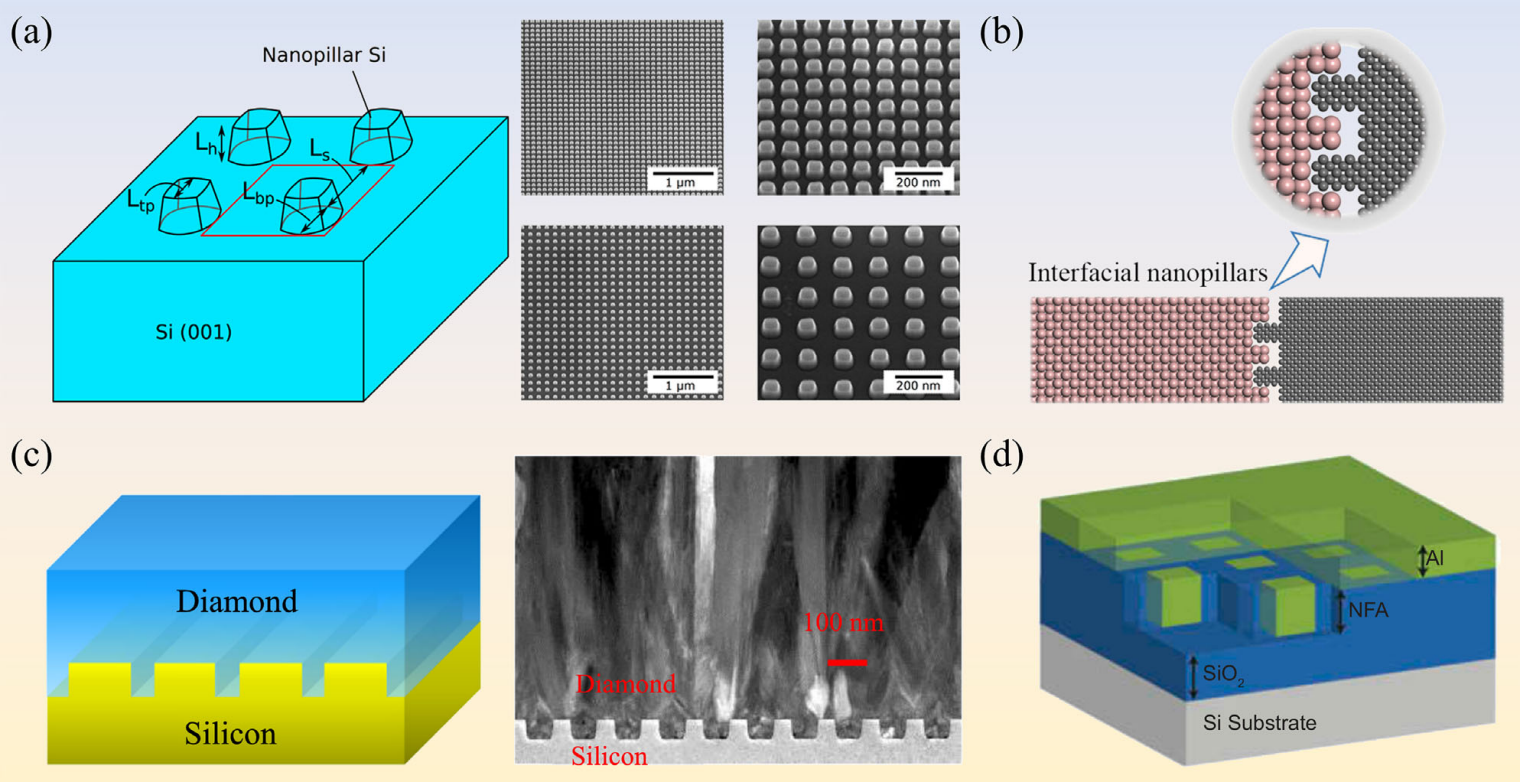
(a) Schematic diagrams and SEM images of 3D nanostructure array interface. Reproduced with permission. [224]. Copyright 2016, American Chemical Society. (b) rectangular nanopillar structure interface. Reproduced with permission. [226]. Copyright 2025, Elsevier B.V. (c) 2D rectangular interface. Reproduced with permission. [227]. Copyright 2019, American Chemical Society. (d) 3D rectangular nanoarray interface. Reproduced with permission. [227]. Copyright, Taylor & Francis.

Introducing nanoscale structures is an effective approach to increase the actual interfacial contact area and improve thermal transport. Qi et al. [223], using MD simulations, showed that rectangular nanopillar arrays reduced the AlN/diamond ITR by 28%, due to modifications in the vibrational density of states of mid‐to‐high‐frequency phonons. Lee et al. [224] demonstrated a marked improvement in thermal transport across solid‐solid interfaces through the introduction of interfacial nanostructures, as illustrated in Figure 9a. Analogous to macroscopic fins employed in heat exchangers to enhance heat dissipation, the incorporation of patterned nanopillar arrays at the interface increases the effective contact area, thereby promoting interfacial heat conduction. Using TDTR, they measured an enhancement in thermal boundary conductance of up to approximately 88%. Theoretical investigations revealed that low‐frequency phonons, owing to their high transmittance, are relatively insensitive to interfacial nano‐structuring. In contrast, the presence of nanostructures has a pronounced effect on high‐frequency phonons, which play a dominant role in interfacial heat transfer at room temperature. As illustrated in Figure 9b, interfacial engineering strategies were employed to optimize heat transfer, achieving up to a 1.44‐fold increase in interfacial thermal conductivity. Analysis of the PDOS on either side of the interface demonstrated that both the vibrational coupling strength and the spectral distribution of phonons significantly influence interfacial heat transport. Luo et al. [225] proposed a multiscale modeling framework to investigate heat transport across nanostructured interfaces. Their study focused on how variations in nanostructure morphology and dimensions influence phonon transmission pathways at different frequencies and their respective contributions to thermal conductivity. The findings revealed that, among the geometries examined, rectangular nanostructures were most effective in enhancing the likelihood of reflected phonons re‐encountering the interface, thereby improving overall phonon transmission. Due to the presence of nanostructures, interfacial heat flux becomes highly nonuniform, with pronounced lateral heat flow developing along the sidewalls‐effectively creating additional channels for thermal transport. The combined effects of multiple phonon reflections and dual transmission mechanisms give rise to an optimal geometry that maximizes ITC. Specifically, at a nanostructure height of 100 nm, the optimal width was also found to be 100 nm, yielding a maximum enhancement in interfacial thermal conductivity by a factor of 1.31.

Cheng et al. [228] constructed Si/diamond interfaces with 2D rectangular nanostructures, as shown in Figure 9c, achieving a 65% higher ITC compared to planar interfaces. Wang et al. [226] explored the thermal transport behavior at aluminum/diamond composite interfaces using non‐equilibrium MD simulations. The effects of system size, temperature, and interfacial structure on interfacial heat transfer were systematically examined. Results revealed that ITC increases with both system size and ambient temperature, showing a 1.76‐fold enhancement as temperature rises from 100 to 400 K. Furthermore, Park et al. [227]. investigated a nanostructured interfacial configuration, as illustrated in Figure 9d, where the pillar‐to‐pillar spacing was adjusted to systematically vary the total sidewall surface area. This variation emphasized the contribution of the additional heat transfer pathway provided by the sidewalls, which offered approximately twice the interfacial area compared to a flat interface. These results indicate that the nano‐structuring strategy can be effectively applied to interfaces bridging diffusive and quasi‐ballistic transport regimes, particularly in highly miniaturized devices.

Hua et al. [229] based on phonon Monte Carlo simulations under the gray medium approximation, suggested that such nanostructures enhance phonon multiple reflections and transmissions, effectively extending the heat transport path‐identified as the key mechanism behind ITC improvement. However, the ITC enhancement observed in experiments often falls short of predictions based solely on increased contact area [224], indicating the involvement of multiple contributing mechanisms. To more accurately evaluate thermal transport across nanostructured interfaces, it is necessary to consider phonon scattering, transmission, and resonance across different frequencies and modes, especially given the challenges of fabricating ideal geometries in practical conditions. According to the DMM, phonon transmission at interfaces is highly sensitive to incident angles [230], suggesting that the tilt angle of nanostructure sidewalls can significantly affect phonon transport and overall ITC. Moreover, phonons of different frequencies exhibit distinct propagation paths and transmission efficiencies when encountering nanostructured interfaces [231]. These contributions are particularly sensitive to the size and geometry of the nanostructures. Therefore, the rational design of nanoscale dimensions and interface morphology is expected to be a key direction for future optimization of interfacial thermal transport. However, from a practical engineering perspective, TIM materials are required not only to provide high interfacial thermal conductance (typically ≥30–100 MW·m^−2^·K^−1^), but also to maintain reversible compressibility (10–60%), long‐term thermo‐hydro stability, low contact thermal resistance, and thermal expansion compatibility with chip and substrate materials to prevent interfacial delamination under cyclic loading. In high heat‐flux devices, the TIM layer thickness is typically confined within 10–100 µm to minimize the heat transfer path.

### 4.2 Molecular Dynamics Simulation

During the process of electronic packaging, as it is impossible to observe the changes in material response in real time, MD simulation, as a powerful tool, provides an effective means to reveal the atomic‐level dynamic response mechanism in the process.

**FIGURE 10:**
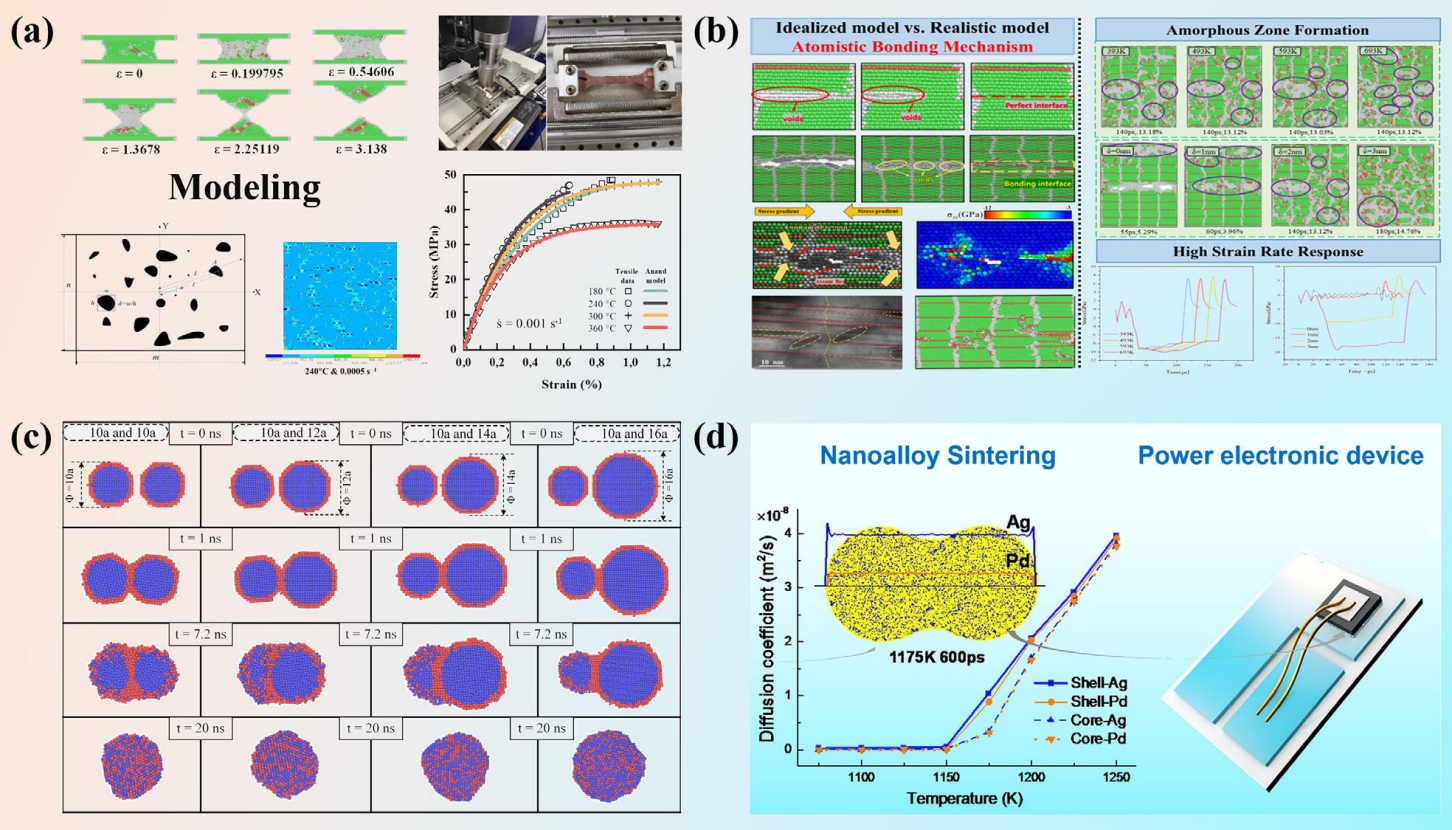
MD simulation: (a) Atomic response during the sintering and stretching process of nano‐Cu. Reproduced with permission. [234]. Copyright 2023, Elsevier B.V. (b) Molecular‐dynamics model of (111)‐oriented nanotwinned Ag. Reproduced with permission. [235]. Copyright 2025, American Chemical Society. (c) Sintering process of nano‐Ag of different diameters. Reproduced with permission. [236]. Copyright 2021, Elsevier Ltd. (d) Sintering diffusion behavior of Ag20Pd nanoalloy. Reproduced with permission. [237]. Copyright 2021, Elsevier B.V.

In the field of nanoparticle sintering, the melting and sintering behavior of Cu nanoparticles in bonding has been revealed through MD simulations. During the sintering process, as the temperature rises, atoms keep moving, causing dislocations at the sintering neck in HCP structure, and after sintering is completed, it transforms into a disordered structure [232, 233]. The research also found that under different sintering temperatures and strain rates, the tensile response of nano‐Cu paste exhibited visco‐plastic deformation behavior, which was similar to the plastic deformation mechanism of macroscopic materials [234], the visco‐plastic deformation behavior of the nano‐Cu paste is depicted in Figure 10a.

Building on these nanoparticle‐level insights, MD simulations have been further extended to sintered Ag in power‐electronic applications, providing atomic‐scale understanding of its microstructural evolution under high‐temperature conditions. In the study on MD of power electronics, Liu et al. conducted an in‐depth study on the bonding process and high‐strain‐rate response of (111)‐oriented nanotwinned Ag using MD simulations. They revealed the atomic‐level bonding process. Provide assistance for the design of high‐strength and high‐toughness Ag‐based packaging materials [235]. The molecular‐dynamics model of (111)‐oriented nanotwinned Ag is presented in Figure 10b. In the sintering study of nano‐Ag solder paste, MD simulation revealed that when the diameter ratio of nano‐ Ag spheres was 1:1.4, the sintered Ag layer exhibited a thermal conductivity of more than 400 W·m^−1^·K^−1^ [236]. The sintering process of two nano‐Ag spheres was simulated by LAMMPS as shown in Figure 10c. In the study of the sintering mechanism of Ag‐PD nanoalloy films, MD simulations revealed that the diffusion ability of Ag atoms in the surface layer of Ag‐PD nanoalloys is superior to that of Pd atoms, and the Ag‐enriched layer formed on the surface plays a key role in promoting the formation of interparticle necks. It was determined that the diffusional activation energy of Ag in the alloy is lower than that of Pd. It provides atomic‐level insights into the sintering behavior of alloys [237]. Figure 10d illustrates the sintering diffusion behavior of the Ag20Pd nanoalloy.

### 4.3 Phase Field Simulation

The phase field method (PFM) indirectly reflects the evolution of microstructure through the change of order parameters, and can effectively simulate complex interfaces and multi‐physics field coupling effects, avoiding complex interface tracking problems [238, 239]. Therefore, the PFM has unique advantages in simulating the evolution of microstructure. This subsection focuses on the latest progress in the application of PFM in the sintering, amplitude modulation decomposition and electrochemical migration processes of nanoparticles, analyzes representative research achievements in various fields, and demonstrates the potential and prospects of PFM in optimizing packaging materials and enhancing device reliability.

**FIGURE 11:**
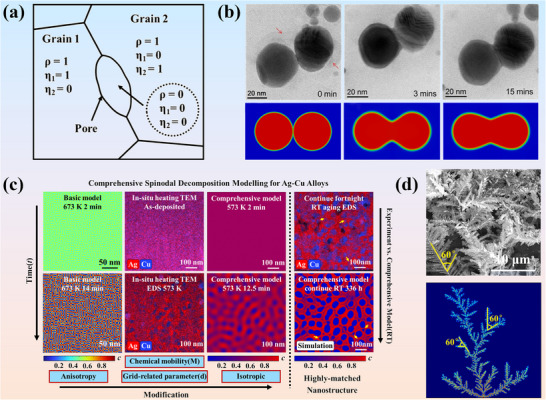
Phase field simulation in packaging materials development: (a) Schematic diagrams of two order parameters in the phase field model. Reproduced with permission. [240]. Copyright 2013, Elsevier B.V. (b) Morphologies of two equally sized Ag particles with a diameter of 40 nm sintered at 400°C for 0, 3 and 15 min respectively. Reproduced with permission. [241]. Copyright 2016, Elsevier B.V. (c) Spinodal Decomposition Modelling for Ag‐Cu Alloys. Reproduced with permission. [242]. Copyright 2025, American Chemical Society. (d) Simulation results of Ag dendrite morphology. Reproduced with permission. [243]. Copyright 2023, Elsevier Ltd.

The order parameters within the phase field framework can be roughly divided into two major categories, namely, conserved order parameters and non‐conserved order parameters. In sintering PFM, the most common types of conserved order parameters are concentration fields or mass density fields. As shown in Figure 11a, the mass density value ρ = 1 represents the sintered solid at this position, while ρ = 0 represents pores or air. This order parameter adheres to the law of conservation of mass throughout the evolution of the microstructure. On the contrary, another type of non‐conserved order parameter specifically conveys information about the structure and crystal orientation at specific spatial positions [240].

The traditional sintering phase field model is mainly based on the Cahn‐Hilliard equation [244] and the Allen‐Cahn [245] equation, which are used to control the evolution behavior of conserved order parameters and non‐conserved order parameters. In early studies, Kumar et al. [246] simulated the sintering process of nanoparticles of different particle sizes using a traditional sintering phase field model and found that smaller particles tended to disappear during the sintering process, while larger particles were growing. Deng et al. [247] introduced direction‐dependent diffusion coefficients in the phase field model to reflect the anisotropic characteristics of particle interface diffusion. Moreover, this anisotropic diffusion model is also applicable to the sintering simulation of Ag nanoparticles. Chockalingam et al. [241] demonstrated through a 2D phase field model that this method can effectively predict the interface evolution during the sintering process of Ag nanoparticles. Its sintered particles are shown in Figure 11b.

Subsequently, Biswas et al. [248] conducted an in‐depth study on the microstructure evolution at each stage of the sintering process using the phase field model and found that surface diffusion played a dominant role in neck growth in the initial stage, while grain boundary migration in the later stage was the main factor leading to material densification [249]. Biswas et al. [250] also pointed out that under the action of the stress field, the diffusion in the neck region significantly accelerates, and the contact points between particles are affected by the high strain energy density, resulting in a rapid densification process. The electric‐thermal‐force coupling model proposed by Liang et al. shows that the Joule heat effect generated by the current density in the particle contact area significantly accelerates diffusion and neck growth, and improves the densification efficiency [251].

Another good example of phase field simulation can be illuminated by the case of spinodal decomposition study. Spinodal decomposition is an important phase separation mechanism. Theoretically, it occurs when the second derivative of Gibbs free energy with respect to the components is negative [252]. The amplitude‐modulated decomposition model based on the Cahn‐Hilliard equation effectively captures the continuous transformations occurring at the phase interface [242, 253]. As shown in Figure 11c, a detailed spinodal decomposition model for the Ag‐Cu alloy is presented, illustrating the characteristic composition fluctuations and interface development. In recent years, significant progress has been made in fields such as elastic heterogeneous thin films, magnetron thin film deposition, and high‐entropy alloys. In the early stage, Hu et al. [252] proposed an effective phase field model to study the coherent microstructure evolution in elastic anisotropic films with significant elastic modulus inhomogeneity. Subsequently, Seol et al. [254, 255] further explored the amplitude modulation decomposition behavior in constrained films and found that within a certain composition range, phase separation follows a surface‐oriented amplitude modulation decomposition mechanism driven by elastic energy effects. The precipitated phases will be arranged along the elastic soft direction to reduce the elastic energy, indicating that the microscopic stress field of the material can effectively regulate the phase separation behavior in films.

In addition, the deposition conditions of thin films also have a regulatory effect on the microstructure of amplitude modulation decomposition. Stewart et al. [256] simulated the phase separation kinetics in thin films through a phase field model and found that there are at least four separated phase forms in thin films: transverse, vertical, random, and nanoparticle. Lu et al. [257] studied the deposition process of immiscible alloy films and found that the higher the temperature, the smaller the difference between surface diffusion and bulk diffusion.

In high‐entropy alloys, it is relatively mature to simulate the amplitude modulation decomposition process using the PFM. Li et al. [258] investigated the amplitude‐modulated decomposition process in Al‐Ni‐Co‐Fe‐Cr high‐entropy alloys, revealing that under different elastic conditions, the microstructure morphology would exhibit spherical, cubic or woven characteristics, indicating the significant role of stress distribution in microstructure evolution. In further research, Kadirvel et al. [259] simulated two different amplitude‐modulated decomposition phase separation paths in high‐entropy alloys through a phase field model, and systematically analyzed the influences of factors such as volume fraction, free energy curve shape, and elastic modulus differences on microstructure evolution. Koneru et al. [260] revealed the potential of heat treatment schemes to regulate the microstructure of amplitude modulation decomposition, providing a new direction for the development of high‐entropy alloys with better comprehensive performance.

From reliability aspect of packaging materials development, electrochemical migration is one of the main potential failure mechanisms in the packaging of power electronic devices. Especially under high humidity and high voltage conditions, the migration and dendrite growth of metal ions driven by an electric field may lead to short‐circuit failure. The PFM can reveal the dynamic mechanism and morphological changes of dendrite growth from a microscopic perspective, providing theoretical support for the optimal design of packaging materials.

The initial research focused on the evolution of dendrite morphology. Chen et al. [261] first used a nonlinear phase field model combined with Butler‐Volmer kinetics to study the growth pattern of lithium dendrites under different electrode surface roughness and potential conditions, revealing the significant influence of electrode morphology and applied voltage on the formation path and branching of dendrites. Jana et al. [262] introduced the stress field into the phase field model. By analyzing the interaction between current density and the stress field, they provided a new perspective for dendrite regulation in complex electrochemical environments. Mu et al. [263] simulated the growth, healing and contraction processes of lithium dendrites under different electrochemical states using a 3D phase field model, revealing the influence of potential gradient and ion concentration on the precipitation of lithium dendrites. Lin et al. [264] further expanded the force‐electrochemical coupling phase field model for the electrochemical migration process of uranium. Illes et al. [265] developed a 2D model based on the Nernst‐Plank equation and systematically studied the electrochemical migration process of Cu electrodes in a high‐humidity environment.

Based on this, Huo et al. combined the Allen‐Cahn equation and the Butler Volmer kinetic equation to construct the electrochemical migration phase field model of Ag ‐based conductive adhesives for the first time, simulating the entire process of Ag ion release from the anode to dendrite formation at the cathode [243], as shown in Figure 11d. Verified by the water drop experiment, this model can accurately predict dendrite morphology, critical voltage and failure time. With the increase of potential, the growth rate of dendrites significantly improves, but when the voltage is lower than a certain critical value, dendrites cannot form. Compared with the traditional failure time model, the phase field model has unique visualization advantages, which can dynamically present the growth process of dendrites and achieve in‐depth understanding of electrochemical migration phenomena from the perspective of multi‐field coupling [266]. This will provide the possibility for the application of phase fields in other Ag‐based packaging materials in the future, and has potential value in engineering applications.

### 4.4 Finite Element Simulation

Electro‐thermal‐mechanical interactions, and in some cases, magnetic effects, govern heat flow, stress evolution, and electrical reliability in advanced packaging. As power density and integration increase, multiphysics finite‐element (FE) workflows become essential for co‐designing materials, interfaces, and structures, enabling accurate junction‐to‐ambient predictions, failure hot‐spot localization, and process‐window definition [267, 268]. Beyond improving design fidelity, such models support failure prognosis, efficiency optimization, and informed material selection, laying the foundation for robust, high‐density systems that endure harsh service conditions [269].

**FIGURE 12:**
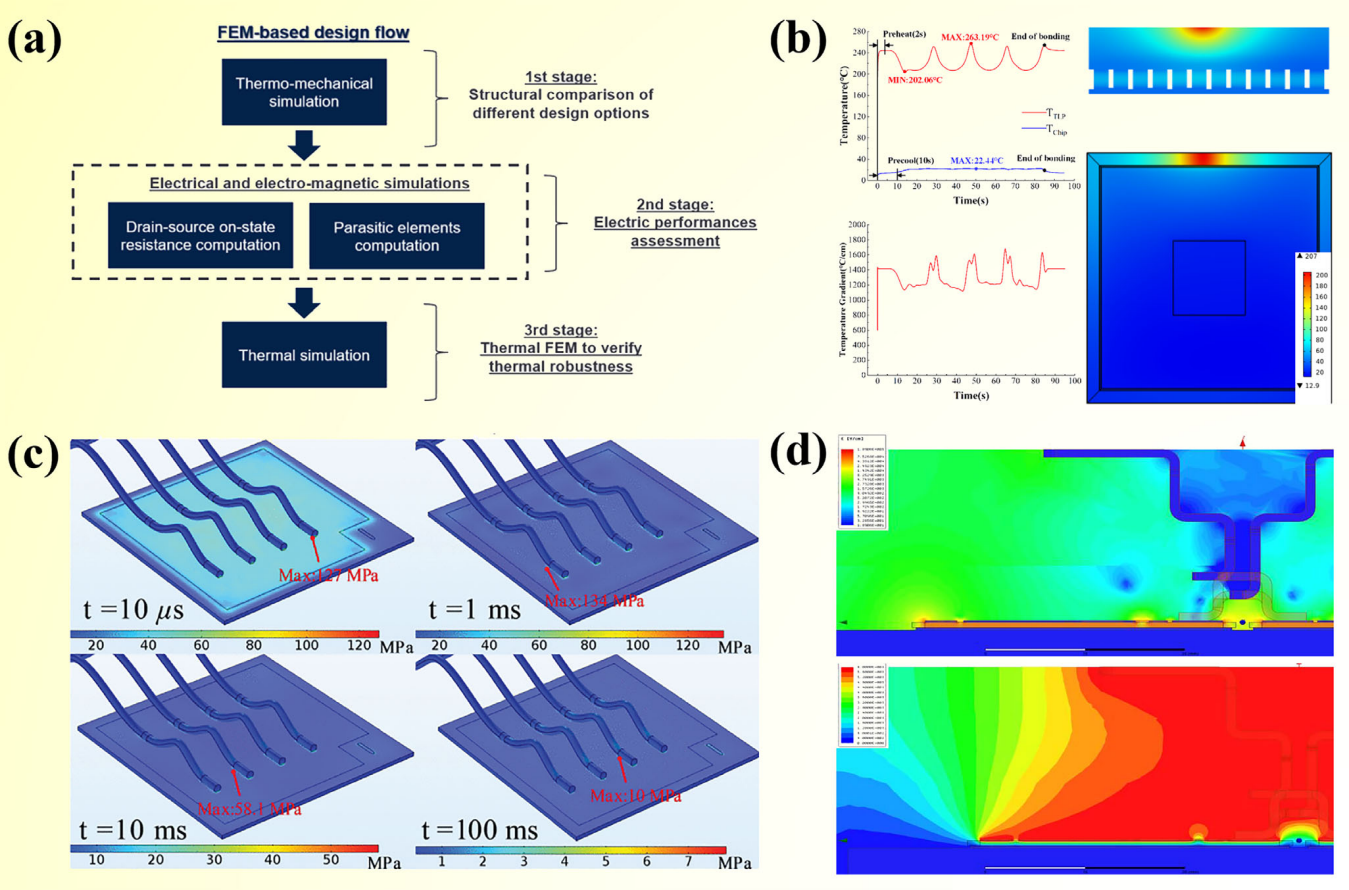
Multiphysics coupled finite element simulation in electronic packaging: (a) FEM flow for robust design of a low‐voltage package. Reproduced with permission. [270]. Copyright 2023, ASME. (b) Laser‐assisted FEM for hermetic packaging of thermally sensitive components. Reproduced with permission. [272]. Copyright 2024, IEEE. (c) Electro‐thermal FEM shows bond‐pad stress concentration. Reproduced with permission. [273]. Copyright 2020, IEEE. (d) Electro‐magnetic simulation in IGBT module design. Reproduced with permission. [276]. Copyright 2016, IEEE.

Recent studies exemplify the adoption of multiphysics modeling for packaging co‐design. Mauromicale [270] have focused on the packaging research of low‐voltage power semiconductor systems in automotive applications. They have adopted the finite element method (FEM) to achieve the simulation of thermal‐force‐electromagnetic coupling (Figure 12a). Among them, the steady‐state temperature distribution under actual working conditions is verified through thermal simulation to ensure compliance with the requirements of efficient heat dissipation management. Complementing prior electro‐thermal‐mechanical FEM exemplars, Huo et al. developed a localized‐laser finite element framework that predicted temperature contours while constraining the global thermal load, thereby mitigating thermo‐mechanical stress during hermetic sealing of temperature‐sensitive devices [271]. Building on this foundation, their subsequent multiphysics model integrated a thermal‐gradient mechanism with a thermoelectric cooler [272], showing that the chip region was stabilized below 296 K whereas the bonding zone exceeded 473 K. Further simulations demonstrated that, under optimized laser parameters, the vertical temperature gradient could be maintained above 1000 K·cm^−1^, which effectively promoted directional solidification and the formation of uniform grain structures (Figure 12b). Collectively, these studies highlight how finite element multiphysics modeling can capture the coupled dynamics of localized heating, active cooling, and stress evolution, offering a transferable approach for designing hermetic packaging processes that protect thermally sensitive components in extreme environments.

Recently, Jia et al. [273] developed a 3D electrothermal multi‐physics field modeling method, focusing on the dynamic simulation of IGBT power modules. The research revealed the thermal stress concentration effect under extreme temperature gradients, thereby clarifying the failure mechanism of IGBT modules under high‐temperature shock conditions. Figure 12c shows the thermo‐mechanical outcome of electro‐thermal coupling, i.e., von Mises stress localizes at the bond‐wire/chip solder interfaces and persists from 10 µs to 100 ms as heat diffuses outward, directly linking temperature gradients and CTE mismatch to stress concentration. This explains the experimentally observed edge‐initiated cracking at pads during power cycling and motivates interface‐level design to mitigate hotspot formation. Zhao et al. [274] studied and analyzed the edge effect of thin‐film ceramic electrode materials. For power modules using ultra‐thin ceramic substrates, thermodynamic coupling simulation showed that stress concentration in the solder layer and ceramic substrate was the main failure point under thermal cycling conditions. Yu et al. [275] conducted research on the reliability of solder joints in advanced electronic packaging, deeply analyzing the operational characteristics and failure mechanisms of solder joints in ball grid array packaging. Under multi‐physical field conditions, through effective thermal management and design optimization, the reliability of solder joints was significantly improved. Li et al. [276] studied the optimization effect of comprehensive simulation of electromagnetic fields, thermal fields and force fields on the design of high‐power IGBT modules, covering electrical performance, thermal management and structural reliability (Figure 12d).

Shrinking dimensions and rising structural complexity demand models that link atomistic physics to device‐scale responses. bridges this gap by combining methods at different resolutions so that microscopic mechanisms can inform macroscopic design [277, 278, 279]. This part of the article systematically reviews the development progress of multi‐scale simulation technology, aiming to provide new ideas for the design optimization of packaging materials and structures.

**FIGURE 13:**
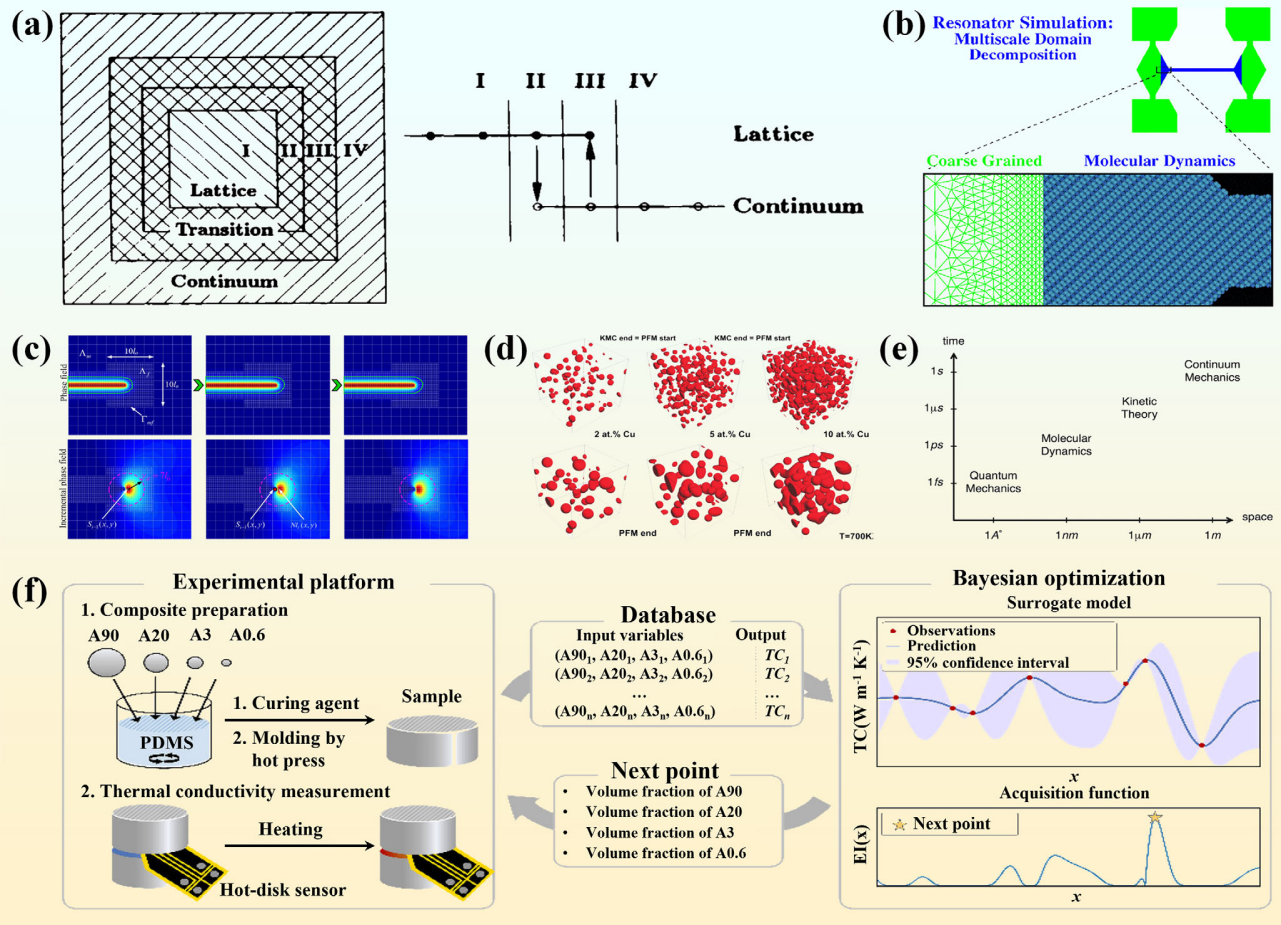
(a) Schematic diagram of the method for combined finite‐element and atomistic analysis of crystal defects. Reproduced with permission. [280]. Copyright 1991, Taylor & Francis. (b) Concurrent CGMD coupling of atomistic and coarse‐grained mechanics. Reproduced with permission. [281]. Copyright 2005, American Physical Society. (c) Phase‐field and finite‐element framework with crack‐tip adaptive refinement and constraint‐coupled coarse‐fine meshes. Reproduced with permission. [282]. Copyright 2018, Elsevier Ltd. (d) The coarsening kinetics analysis of Cu‐rich precipitates in α‐Fe using a KMC simulation and a phase‐field method. Reproduced with permission. [283]. Copyright 2012, Elsevier Ltd. (e) The multi‐scale, multi‐physics modeling paradigm. Reproduced with permission. [284]. Copyright, AIP Publishing. (f) Data‐driven engineering workflow for polymer‐based thermal interface composites. Reproduced with permission. [285]. Copyright 2025, Elsevier Ltd.

Early frameworks such as finite element combined with atomistic modelling achieves seamless integration between atomic‐scale discrete simulation and the mechanical behavior of continuum media by constructing a multi‐region division framework (Figure 13a) [280]. Rudd and Broughton [281] developed a coarse‐grained molecular dynamics (CGMD) method, which achieves cross‐scale simulation by combining MD models with coarser finite element representations (Figure 13b). Patil et al. [282] proposed a multi‐scale phase field method, which combined the anisotropic PFM with the multi‐scale FEM wherein the propagating crack is tracked and enclosed by an adaptively refined neighborhood tied to the phase‐field regularization length. constraint‐coupled coarse‐fine interfaces resolve near‐tip fields without global remeshing, reducing computational cost (Figure 13c).

Molnar et al. [283] combined kinetic Monte Carlo (KMC), MD, and PFM to systematically study the coarsening behavior of Cu‐rich precipitates in Fe‐Cu alloys at 700°C (Figure 13d). This multi‐scale framework took advantage of the strengths of each simulation method within their respective scales and achieved cross‐scale coupling through the gradual transfer of parameters.

Data‐driven modeling has become a key enabler. The introduction of ML marks a major breakthrough in cross‐scale simulation technology. By optimizing force field generation, boundary optimization, and electronic structure calculation, it has achieved precise and scalable models that maintain physical constraints while ensuring data integrity [286]. E et al. proposed a method that utilizes ML techniques [284], effectively addressing the efficiency and accuracy bottlenecks of traditional models caused by scale mismatch and computational complexity. The multi‐scale, multi‐physics modeling paradigm is shown in Figure 13e. On this basis, this study employs ML algorithms such as deep neural networks (DNN) to automatically extract the information transmission and constitutive relations between micro and macro models, thereby reducing the reliance on empirical models. Research shows that applying ML in cross‐scale coupling can achieve more reliable prediction and simulation results. This technology not only supports the optimization of material performance and structural design under complex working conditions such as power packaging, but also opens up a new path for the development and application of cross‐scale simulation technology.

Current multi‐scale simulation methods face significant challenges when coupling different scales. The key challenge lies in the interface management between the atomic scale and the continuum domain. Differences in physical models can lead to discontinuities and other artifacts. This issue is particularly prominent in the coupling method of the finite element approach and MD [287], and it is necessary to ensure physical consistency at the interface through complex boundary conditions and energy expressions. Quasi‐continuum methods and other techniques offer highly promising solutions: by selectively reducing atomic‐level resolution in non‐critical regions while maintaining intact atomic details in high‐stress or defect areas such as crack tips or dislocations. However, maintaining dynamic realism in processes such as crack propagation without increasing excessive computational costs [288, 289] remains a major challenge. As detailed in recent studies, the hybrid approach combining the FEM with the phase field model provides an effective solution for handling complex crack paths and maintaining simulation consistency. For instance, the Muise team employed the FEM to simulate sharp cracks and combined it with the phase field model to handle the crack evolution around the tip, thereby achieving local adaptive optimization and avoiding mesh redistribution [290].

Another major challenge faced by multi‐scale simulations lies in their huge computational demands. Such simulations need to combine fine processes at the micro level with macroscopic phenomena. Multi‐scale modeling techniques based on ML have demonstrated the potential to accelerate simulation and enhance predictive capabilities. For example, Graph neural networks have successfully reduced the computational cost of complex problems such as crack propagation by adopting adaptive mesh refinement and multi‐scale coarsening methods [291]. As shown in the phase field fracture model study, these methods achieve an effective balance between accuracy and efficiency by optimizing the resolution only in the critical regions. However, such methods still face challenges such as difficulties in data acquisition and insufficient quality of training datasets—these datasets need to accurately capture behavioral characteristics at the microscale under different conditions. In this case [292, 293], how to ensure computational efficiency while maintaining model reliability remains an urgent problem to be solved, which also highlights the necessity of continuous innovation in multi‐scale simulation frameworks.

The inherent limitations of the complex interdependent relationships among different scales pose significant challenges to multi‐scale modeling. This cross‐scale integration challenge requires the adoption of advanced technical means to capture multi‐scale interactions in real time while avoiding the accumulation of methodological errors caused by cross‐domain data transmission. In the modeling of heterogeneous materials, accurately capturing the multi‐scale interactions between different phases and microstructures is crucial for predicting the behavior of materials under various working conditions. In addition, Ling Hu's team [293] combined the high‐order multi‐scale deep Ritz method with deep learning technology and successfully achieved the simulation of the heat conduction equation with high oscillation and discontinuous coefficients in composite materials. In conclusion, without a robust multi‐scale parallel analysis method, the application of such models in engineering practice and scientific research fields will still face many limitations.

In recent years, AI and ML have been increasingly adopted in the research of thermal management materials, including ceramic substrates and TIMs, to accelerate materials screening, interface structure optimization and quantitative modeling of interfacial heat transport. For example, by integrating ML‐driven optimization algorithms with advanced imaging technologies, high‐performance polymer thermal interface materials with excellent thermal conductivity, enhanced mechanical strength, and ultra‐low thermal expansion coefficients have been developed (Figure 13f). Leveraging large‐scale datasets generated from high‐throughput first‐principles calculations and MD simulations, supervised and semi‐supervised models have been used to establish correlations between microstructure, filler geometry, interfacial bonding strength, phonon spectrum matching and ITC, thereby enabling rapid prediction of effective thermal performance [294, 295]. At the same time, generative models and reinforcement learning are beginning to guide interface morphology and architecture design, moving toward data‐driven optimization of heat‐conduction pathways across multiple length scales. However, substantial challenges remain. Interfacial heat transport is strongly governed by local atomic structure and multiscale phonon coupling, while data obtained from different simulation and experimental platforms often lack uniformity, limiting model generalization. The absence of standardized, cross‐scale training datasets further hampers the ability of machine‐learning models to capture realistic interfacial transport dynamics. In addition, there is still a trade‐off between predictive accuracy and interpretability, underscoring the need for explainable frameworks that can reveal dominant microscopic mechanisms. The deeper integration of AI and ML with DFT, MD and advanced experimental characterization is therefore expected to play a pivotal role in achieving both efficient design of thermal management materials and a more mechanistic understanding of interfacial thermal transport.

## 5 Summary and Outlook

In this review, we have focused on recent advances in thermal management materials and technologies for power electronic packaging, with particular emphasis on ceramic substrates [10, 11, 12, 13], TIMs [15, 16, 17], and their associated interfacial and structural engineering strategies [224, 225, 226, 227]. Representative multiscale modeling and simulation methods [232, 238] were also surveyed to illustrate how heat transport phenomena across length scales can be understood and optimized. Together, these developments show how innovations at the material, interface, and module levels are narrowing the gap between the heat flux levels encountered in wide bandgap power devices and the dissipation capacity of practical packages. From high‐thermal‐conductivity Si_3_N_4_ substrates to architected polymer, ceramic, and diamond‐based TIMs, these advances underscore that packaging materials are no longer passive carriers, but have become active determinants of thermal reliability and system‐level performance in next‐generation power electronics.

Despite this progress, several critical challenges remain along the path from material‐level breakthroughs to scalable, reliable, and sustainable packaging solutions. These challenges, which have been briefly mentioned throughout this review, can be broadly categorized into three interrelated aspects. First, at the materials level, further improvements in intrinsic thermal performance must be carefully balanced against mechanical reliability, manufacturability, and cost. For ceramic substrates such as Si_3_N_4_, pushing thermal conductivity beyond ∼100 W·m^−1^·K^−1^ while maintaining high fracture strength and long‐term reliability remains difficult [58]. Silicon nitride exists primarily in two phases, α‐Si_3_N_4_ and β‐Si_3_N_4_, which exhibit pronounced anisotropy in thermal transport. Early theoretical work [97] predicted extremely high thermal conductivity along the c‐axis, reaching up to ∼225 W·m^−1^·K^−1^ for α‐Si_3_N_4_ and ∼450 W·m^−1^·K^−1^ for β‐Si_3_N_4_. More recent first‐principles calculations [296] reported more conservative upper limits, with β‐Si_3_N_4_ reaching ∼169 W·m^−1^·K^−1^and α‐Si_3_N_4_ ∼116 W·m^−1^·K^−1^ along the c‐axis. The significant discrepancies between these reported theoretical values reflect differences in computational approaches, assumptions regarding phonon scattering, and the treatment of defect‐free crystals. Importantly, both sets of predictions remain much higher than experimentally measured thermal conductivities in practical ceramics, highlighting that the thermal transport behavior of Si_3_N_4_ has not been fully understood. This underscores the critical role of phase composition, crystallographic texture, point defects, and grain‐boundary chemistry in determining effective thermal performance [59, 60, 61]. In polymer‐based TIMs, the key challenge lies in converting the high intrinsic thermal conductivity of fillers into effective composite performance under realistic assembly pressures and bondline thickness constraints. In practice, this conversion is simultaneously limited by interfacial thermal resistance, incomplete filler‐filler percolation, and the strong dependence of thermal performance on processing, contact pressure, and long‐term thermo‐mechanical stability [159, 179, 297]. Diamond‐based fillers have emerged as particularly promising due to diamond's ultrahigh thermal conductivity and ultra‐low thermal expansion [137, 138]. Through interface‐dominated strategies including surface metallization, carbide interlayers, filler alignment, and percolation‐network design, diamond‐reinforced metal, polymer, and hybrid composites have demonstrated substantial enhancements in thermal conductivity and interfacial thermal conductance [152, 153, 156]. These results underscore that the performance of TIMs is no longer governed by filler conductivity alone, but by the coupled optimization of filler architecture, interfacial bonding chemistry, and mechanical compliance at the package level, highlighting the need for TIMs design that explicitly account for assembly conditions and long‐term reliability.

Second, at the package level, metals, ceramics, polymers and semiconductors form complex, anisotropic heat flow paths in which thermal, mechanical and electrical fields are strongly coupled [2, 3, 78, 110]. Conventional continuum models often struggle to capture the combined influence of thermal cycling, current stressing and environmental exposure on degradation modes such as delamination, cracking and interfacial oxidation. Furthermore, many promising nanoscale concepts encounter bottlenecks when scaled to wafer‐ or module‐level manufacturing, where process windows, yield, reproducibility and compatibility with existing production lines impose strict constraints. Finally, increased reliance on energy‐intensive processing routes and scarce elements highlights the need to align high‐performance thermal solutions with sustainability and resource‐efficiency considerations. This underscores the need for package‐level co‐design strategies that integrate thermal management, mechanical reliability, and electrical performance from the earliest stages of device and module development.

Third, with the increasing complexity of material systems and packaging architectures, data‐driven and artificial‐intelligence‐based approaches are emerging as powerful tools to accelerate innovation in thermal management. By learning from experimental and simulation databases, ML models can accelerate the prediction of key properties such as thermal conductivity, breakdown strength and interfacial conductance from compositional, microstructural and processing descriptors, thereby reducing reliance on trial‐and‐error screening [19, 20, 21]. Beyond property prediction, AI techniques enable the intelligent optimization of interfacial thermal resistance by relating microstructural parameters such as surface roughness, material combinations, interface thickness, and morphological features to thermal performance [124, 125, 126]. These models can also process and analyze complex experimental datasets, uncovering nonlinear correlations between thermal properties and structural descriptors, and thereby guiding both high‐throughput material screening and experimental design [129]. More broadly, the integration of AI into packaging materials research is accelerating the understanding of interfacial heat transfer mechanisms and supporting paradigm shifts in material design, enabling optimized interface architectures and enhanced thermal management at both the material and package levels. However, realizing this potential will depend on overcoming current limitations in data volume, quality, standardization and interpretability and on tighter coupling between AI models, DFT/MD simulations and advanced in situ characterization. The development of physics‐informed and uncertainty‐aware AI frameworks will be essential to ensure robustness, transferability, and practical relevance across different material systems and package platforms [298, 299, 300].

In conclusion, thermal management materials and technologies for power electronics are at a stage where fundamental advances in thermal‐transport mechanisms, interface engineering and multiscale modeling must be reconciled with manufacturability, scalability, reliability, and sustainability. Future research will rely on synergistic strategies that couple interfacial‐architecture design with robust processing windows and embed AI‐assisted multiscale modeling into the early stages of material and package development. With these concerted efforts, packaging materials will evolve from passive thermal conduits into actively engineered, intelligent, and environmentally responsible thermal solutions, thereby empowering the sustainable development of high‐performance power electronic packaging for decades to come.

## Funding

This work was supported by the National Natural Science Foundation of China (No. 52473330) and Beijing Nova Program (No. 20230484280).

## Conflicts of Interest

The authors declare no conflict of interest.

## Data Availability

The authors have nothing to report.

## References

[1] M. S. Lundstrom and M. A. Alam , “Moore's Law: The Journey Ahead,” Science 378 (2022): 722–723, 10.1126/science.ade2191. 36395227

[2] Y. Wen , C. Chen , Y. Ye , et al., “Advances on Thermally Conductive Epoxy‐Based Composites as Electronic Packaging Underfill Materials—A Review,” Advanced Materials 34 (2022): 2201023, 10.1002/adma.202201023. 35581925

[3] Y.‐M. Ju , T.‐W. Kim , S.‐H. Lee , H.‐J. Lee , J. Ahn , and H.‐S. Kim , “Advanced WBG Power Semiconductor Packaging: Nanomaterials and Nanotechnologies for High‐Performance Die Attach Paste,” Nano Convergence 12 (2025): 38, 10.1186/s40580-025-00503-3. 40699507 PMC12287503

[4] A.‐C. Iradukunda , D. R. Huitink , and F. Luo , “A Review of Advanced Thermal Management Solutions and the Implications for Integration in High‐Voltage Packages,” IEEE Journal of Emerging and Selected Topics in Power Electronics 8 (2020): 256–271, 10.1109/JESTPE.2019.2953102.

[5] Z. Wang , J. Nan , Z. Tian , P. Liu , Y. Wu , and J. Zhang , “Review on Main Gate Characteristics of P‐Type GaN Gate High‐Electron‐Mobility Transistors,” Micromachines 15 (2024): 80, 10.3390/mi15010080. PMC10818513 38258199

[6] H. A. Soomro , M. H. B. M. Khir , S. A. B. M. Zulkifli , G. E. M. Abro , and M. M. Abualnaeem , “Applications of Wide Bandgap Semiconductors in Electric Traction Drives: Current Trends and Future Perspectives,” Results in Engineering 26 (2025): 104679, 10.1016/j.rineng.2025.104679.

[7] A. N. Suthar , J. Venkataramanaiah , and Y. Suresh , “Conventional, Conventional, Wide‐Bandgap, and Hybrid Power Converters: A Comprehensive Review,” Renewable and Sustainable Energy Reviews 213 (2025): 115419, 10.1016/j.rser.2025.115419.

[8] T. Zhan , M. Xu , Z. Cao , et al., “Effects of Thermal Boundary Resistance on Thermal Management of Gallium‐Nitride‐Based Semiconductor Devices: A Review,” Micromachines 14 (2023): 2076, 10.3390/mi14112076. 38004933 PMC10673006

[9] M. Belmonte , “Advanced Ceramic Materials for High Temperature Applications,” Advanced Engineering Materials 8 (2006): 693–703, 10.1002/adem.200500269.

[10] A. Almansoori , K. Balázsi , and C. Balázsi , “Advances, Challenges, and Applications of Graphene and Carbon Nanotube‐Reinforced Engineering Ceramics,” Nanomaterials 14 (2024): 1881, 10.3390/nano14231881. 39683269 PMC11643847

[11] Q. Shen , Z. Lin , J. Deng , et al., “Effects of β‐Si_3_N_4_ Seeds on Microstructure and Performance of Si_3_N_4_ Ceramics in Semiconductor Package,” Materials 16 (2023): 4461, 10.3390/ma16124461. 37374644 PMC10302068

[12] H. Xiong , B. Li , X. Xi , and Q. Shan , “Preparation of graded silicon nitride ceramics with high mechanical performance using β‐Si3N4 seeds,” Ceramics International 49 (2023): 36528–36535, 10.1016/j.ceramint.2023.08.336.

[13] Y. Liu , R. Liu , Z. Tong , et al., “Optimization of Microstructure to Improve Si_3_N_4_ Ceramics with Thermal Conductivity and Mechanical Properties: Effect of Si and α‐Si_3_N_4_ Powder Composition,” Journal of Alloys and Compounds 1010 (2025): 177831, 10.1016/j.jallcom.2024.177831.

[14] J. Wu , G. Yuan , Z. Jiao , et al., “Optimizing Interface Microstructure of Diamond Reinforced Al Matrix Composites via Nano‐Scale Si‐Al Coatings towards Enhanced Thermophysical Performance,” Applied Surface Science 693 (2025): 162786, 10.1016/j.apsusc.2025.162786.

[15] P. Zhu , P. Wang , P. Shao , et al., “Research Progress in Interface Modification and Thermal Conduction Behavior of Diamond/Metal Composites,” International Journal of Minerals, Metallurgy and Materials 29 (2022): 200–211, 10.1007/s12613-021-2339-6.

[16] Y. Zhang , L. Wang , J. Hao , N. Li , X. Wang , and H. Zhang , “Effect of Diamond Particle Size on Thermal Conductivity and Thermal Stability of Zr‐Diamond/Cu Composite,” Diamond and Related Materials 146 (2024): 111257, 10.1016/j.diamond.2024.111257.

[17] A. Sukserm , M. Ceppatelli , M. Serrano‐Ruiz , et al., “Stability, Chemical Bonding, and Electron Lone Pair Localization in AsN at High Pressure by Density Functional Theory Calculations,” Inorganic Chemistry 63 (2024): 8142–8154, 10.1021/acs.inorgchem.4c00342. 38640445

[18] L. Wei , L. Ma , Q. Qi , et al., “Ordered Alignment of 2D Heterogeneous Filler for Enhancing Anisotropic Thermal Conduction Capability of Multifunctional Composite,” Carbon 243 (2025): 120625, 10.1016/j.carbon.2025.120625.

[19] C. Du , G. Zou , J. Huo , B. Feng , Z. A , and L. Liu , “Generative AI‐Enabled Microstructure Design of Porous Thermal Interface Materials with Desired Effective Thermal Conductivity,” Journal of Materials Science 58 (2023): 16160–16171, 10.1007/s10853-023-09018-w.

[20] D. Wu , Z. Xu , and D. Guo , “Machine Learning Accelerates Programmable Mechanics in Isotropic Diamond Plate Lattices,” International Journal of Mechanical Sciences 302 (2025): 110595, 10.1016/j.ijmecsci.2025.110595.

[21] N. Dimitriou , L. Leontaris , T. Vafeiadis , et al., “Fault Diagnosis in Microelectronics Attachment via Deep Learning Analysis of 3‐D Laser Scans,” IEEE Transactions on Industrial Electronics 67 (2020): 5748–5757, 10.1109/TIE.2019.2931220.

[22] A. L. Moore and L. Shi , “Emerging Challenges and Materials for Thermal Management of Electronics,” Materials Today 17 (2014): 163–174, 10.1016/j.mattod.2014.04.003.

[23] C. Zhang , L. Sun , B. Huang , X. Yang , Y. Chu , and B. Zhan , “Electrical and Mechanical Properties of CNT/CB Dual Filler Conductive Adhesives (DFCAs) for Automotive Multi‐Material Joints,” Composite Structures 225 (2019): 111183, 10.1016/j.compstruct.2019.111183.

[24] N. Vasilakis , D. Moschou , D. Carta , H. Morgan , and T. Prodromakis , “Long‐Long‐Lasting FR‐4 Surface Hydrophilisation towards Commercial PCB Passive Microfluidics,” Applied Surface Science 368 (2016): 69–75, 10.1016/j.apsusc.2015.12.123.

[25] X. Liu , W. Chen , X. Feng , et al., “Synergistic Enhancement of Thermal Stability and Dielectric Performance of BT Resin Composites via Difunctional Phthalonitrile Monomers for High‐Frequency PCB Substrates,” Composites Part B: Engineering 309 (2026): 113042, 10.1016/j.compositesb.2025.113042.

[26] P.‐C. Hsu , S.‐C. Chang , W.‐X. Lu , H.‐C. Liu , and C.‐E. Ho , “Enhanced Adhesion Strength Between Electroplated Cu and ABF Substrate with Isothermal Annealing Treatment,” Surface and Coatings Technology 479 (2024): 130576, 10.1016/j.surfcoat.2024.130576.

[27] F. Hu , Z.‐P. Xie , J. Zhang , Z.‐L. Hu , and D. An , “Promising High‐Thermal‐Conductivity Substrate Material for High‐Power Electronic Device: Silicon Nitride Ceramics,” Rare Metals 39 (2020): 463–478, 10.1007/s12598-020-01376-7.

[28] J. Hostaša , W. Pabst , and J. Matějíček , “Thermal Conductivity of Al_2_O_3_–ZrO_2_ Composite Ceramics,” Journal of the American Ceramic Society 94 (2011): 4404–4409, 10.1111/j.1551-2916.2011.04875.x.

[29] R. Khazaka , L. Mendizabal , and D. Henry , “Review on Joint Shear Strength of Nano‐Silver Pasteand Its Long‐Term High Temperature Reliability,” Journal of Electronic Materials 43 (2014): 2459–2466, 10.1007/s11664-014-3202-6.

[30] M. Kutz , Handbook of Materials Selection (John Wiley & Sons, 2002), 10.1002/9780470172551.

[31] J. R. Davis , Aluminum and Aluminum Alloys (ASM International, 1993).

[32] N. Burger , A. Laachachi , M. Ferriol , M. Lutz , V. Toniazzo , and D. Ruch , “Review of Thermal Conductivity in Composites: Mechanisms, Parameters and Theory,” Progress in Polymer Science 61 (2016): 1–28, 10.1016/j.progpolymsci.2016.05.001.

[33] J. Freudenberger and H. Warlimont , “Copper and Copper Alloys,” in Springer Handbook of Materials Data, ed. H. Warlimont and W. Martienssen (Springer International Publishing: Cham, 2018), 10.1007/978-3-319-69743-7_12.

[34] A. Zhang and Y. Li , “Thermal Conductivity of Aluminum Alloys—A Review,” Materials 16 (2023): 2972, 10.3390/ma16082972. 37109807 PMC10144406

[35] W. M. Haynes ed., CRC Handbook of Chemistry and Physics, 97th ed. (CRC Press, 2016), 10.1201/9781315380476.

[36] M. McQUARRIE , “Thermal Conductivity: VII, Analysis of Variation of Conductivity with Temperature for Al_2_O_3_, BeO, and MgO,” Journal of the American Ceramic Society 37 (1954): 91–95, 10.1111/j.1551-2916.1954.tb20106.x.

[37] W. Werdecker and F. Aldinger , “Aluminum Nitride‐An Alternative Ceramic Substrate for High Power Applications in Microcircuits,” IEEE Transactions on Components, Hybrids, and Manufacturing Technology 7 (1984): 399–404, 10.1109/TCHMT.1984.1136380.

[38] X. Zhou , J. Zhang , M. Hou , et al., “Study on the Mechanical Properties at High Temperatures and Relevant Mechanism of Beryllium Oxide Ceramics,” Journal of Nuclear Materials 611 (2025): 155813, 10.1016/j.jnucmat.2025.155813.

[39] W. T. Shoulders , M. Guziewski , and J. J. Swab , “Microstructural and Thermal Stress Effects on Mechanical Properties of Boron Carbide Particle‐Reinforced Silicon Carbide,” Journal of the American Ceramic Society 107 (2024): 1249–1261, 10.1111/jace.19535.

[40] W. Liu , Y. Shen , D. Li , X. Ouyang , Q. Liu , and S. Wang , “Preparation of 99.6% Alumina Ceramic Substrates with High Thermal Conductivity by Tape Casting and Warm Pressing Process,” Ceramics International 51 (2025): 5000–5010, 10.1016/j.ceramint.2024.11.471.

[41] S. Fu , Z. Jia , W. Ding , Y. Bao , and D. Wan , “Synthesis and Characterization of a High‐Strength Alumina Ceramic Reinforced by AlN‐Al_2_O_3_ Coating,” Journal of Materials Science 59 (2024): 14235–14244, 10.1007/s10853-024-10048-1.

[42] D. Liu , J. Hu , G. Zhang , et al., “Effect of Synthesizing Temperature of Alumina Powder with Rose‐Like Structure on the Microstructure and Mechanical Property of Alumina Ceramic,” Journal of Materials Research and Technology 28 (2024): 1907–1914, 10.1016/j.jmrt.2023.12.104.

[43] R. K. Ulrich and W. D. Brown , Advanced Electronic Packaging (John Wiley & Sons, 2006), 10.1109/9780471754503.

[44] A. M. Abyzov , “Aluminum Oxide and Alumina Ceramics (Review). Part 1. Properties of Al_2_O_3_ and Commercial Production of Dispersed Al_2_O_3_ ,” Refractories and Industrial Ceramics 60 (2019): 24–32, 10.1007/s11148-019-00304-2.

[45] J. Du , B. Tang , W. Liu , et al., “Effects of Annealing and Firing in Wet Hydrogen on the Dielectric Breakdown Strengths of Alumina Ceramics,” Journal of Advanced Ceramics 9 (2020): 173–182, 10.1007/s40145-019-0357-x.

[46] K. Lin , X. Zong , P. Sheng , et al., “Effects of SmF_3_ Addition on Aluminum Nitride Ceramics via Pressureless Sintering,” Journal of the European Ceramic Society 43 (2023): 6804–6814, 10.1016/j.jeurceramsoc.2023.07.051.

[47] Z. Zhang , H. Wu , S. Zhang , et al., “The Quantitative Investigation of the Lattice Oxygen and Grain Edge Oxygen on the Thermal Conductivity of Aluminum Nitride Ceramics,” Journal of the European Ceramic Society 43 (2023): 313–320, 10.1016/j.jeurceramsoc.2022.10.023.

[48] G. Yin , T. Zhao , X. Chen , et al., “Enhancing Thermal Conductivity of Aluminum Nitride Ceramics Through Control of Oxygen Impurities and Heavy Rare Earth Doping: A First‐Principles and Experimental Study,” Ceramics International 51 (2025): 26225–26233, 10.1016/j.ceramint.2025.03.305.

[49] H. Jiang , X.‐H. Wang , G.‐F. Fan , et al., “Effect of Oxidation on Flexural Strength and Thermal Properties of AlN Ceramics with Residual Stress and Impedance Spectroscopy Analysis of Defects and Impurities,” Ceramics International 45 (2019): 13019–13023, 10.1016/j.ceramint.2019.03.232.

[50] K. Lin , G. Nie , P. Sheng , S. Zhao , and S. Wu , “Effects of Doping Al‐Metal Powder on Thermal, Mechanical and Dielectric Properties of AlN Ceramics,” Ceramics International 48 (2022): 36210–36217, 10.1016/j.ceramint.2022.08.178.

[51] S.‐F. Wang , K.‐K. Chao , Y.‐L. Liao , H.‐H. Hsu , and E. Y. Chang , “Relations Among Composition, Microstructure, and Mechanical and Dielectric Properties From 1 MHz to 100 GHz of Aluminum Nitride Substrates,” Journal of Alloys and Compounds 1005 (2024): 176147, 10.1016/j.jallcom.2024.176147.

[52] Y. Duan , J. Zhang , X. Li , Y. Shi , J. Xie , and D. Jiang , “Low Temperature Pressureless Sintering of Silicon Nitride Ceramics for Circuit Substrates in Powder Electronic Devices,” Ceramics International 44 (2018): 4375–4380, 10.1016/j.ceramint.2017.12.033.

[53] Y. Nakashima , Y. Zhou , K. Hirao , T. Ohji , and M. Fukushima , “Effects of Sintering Temperature and Holding Time on Sintering Behavior, Mechanical Properties and Thermal Conductivity of Silicon Nitride Ceramics,” Ceramics International 51 (2025): 26757–26763, 10.1016/j.ceramint.2025.03.356.

[54] Y. Zhou , H. Hyuga , Y. Nakashima , K. Hirao , T. Ohji , and M. Fukushima , “Effects of Rare‐Earth Oxides on Microstructure, Thermal Conductivity, and Mechanical Properties of Silicon Nitride,” Journal of the American Ceramic Society 108 (2025): 70028, 10.1111/jace.70028.

[55] Y. Zhuang , F. Sun , L. Zhou , et al., “The Influence of Magnesium Compounds on the Properties of Silicon Nitride Ceramics,” International Journal of Applied Ceramic Technology 21 (2024): 2273–2287, 10.1111/ijac.14665.

[56] F. Hu , T. Zhu , Z. Xie , and J. Liu , “Effect of Composite Sintering Additives Containing Non‐Oxide on Mechanical, Thermal and Dielectric Properties of Silicon Nitride Ceramics Substrate,” Ceramics International 47 (2021): 13635–13643, 10.1016/j.ceramint.2021.01.224.

[57] Y. Nakashima , Y. Zhou , K. Tanabe , et al., “Effect of Microstructures on Dielectric Breakdown Strength of Sintered Reaction‐Bonded Silicon Nitride Ceramics,” Journal of the American Ceramic Society 106 (2023): 1139–1148, 10.1111/jace.18826.

[58] V. P. Adiga , R. De Alba , I. R. Storch , P. A. Yu , B. Ilic , et al., “Simultaneous Electrical and Optical Readout of Graphene‐Coated High Q Silicon Nitride Resonators,” Applied Physics Letters 103 (2013): 143103, 10.1063/1.4823457.

[59] H. Imamura , T. Kawata , S. Honda , and Y. Iwamoto , “Correction to: A Facile Method to Produce Rod‐Like β‐Si_3_N_4_ Seed Crystallites for Bimodal Structure Controlling,” Journal of the American Ceramic Society 106 (2023): 5102, 10.1111/jace.19114.

[60] B. Palanki , “Some Factors Affecting Densification and Grain Growth in the Sintering of Uranium Dioxide—A Brief Review,” Journal of Nuclear Materials 550 (2021): 152918, 10.1016/j.jnucmat.2021.152918.

[61] M. Kitayama , K. Hirao , A. Tsuge , K. Watari , M. Toriyama , and S. Kanzaki , “Thermal Conductivity of β‐Si_3_N_4_: II, Effect of Lattice Oxygen,” Journal of the American Ceramic Society 83 (2000): 1985–1992, 10.1111/j.1151-2916.2000.tb01501.x.

[62] Z. Lei , Y. Ding , X. Ju , Q. Wang , Y. Peng , and M. Chen , “Integrated Cold Sintering of Ceramic Circuit Substrate for Power Device Packaging,” Ceramics International 51 (2025): 17870–17878, 10.1016/j.ceramint.2025.01.557.

[63] M. Choe , S. H. Ryu , J. Jeon , et al., “Stabilization of Top‐Gate p‐SnO Transistors via Ultrathin Al_2_O_3_ Interlayers for Hysteresis‐Free Operation,” Journal of Materials Chemistry C 13 (2025): 12308–12316, 10.1039/D5TC00399G.

[64] S. Li , S. Chen , G. Tan , et al., “High‐Strength High‐Thermal‐Conductivity Al_2_O_3_ Ceramics via Colloidal Processing and Low‐Temperature Pressureless Sintering,” Journal of the American Ceramic Society 108 (2025): 20552, 10.1111/jace.20552.

[65] Y. Xiong , Z. Fu , Y. Wang , and F. Quan , “Fabrication of Transparent AlN Ceramics,” Journal of Materials Science 41 (2006): 2537–2539, 10.1007/s10853-006-5314-8.

[66] K. Watari , K. Ishizaki , and T. Fujikawa , “Thermal Conduction Mechanism of Aluminium Nitride Ceramics,” Journal of Materials Science 27 (1992): 2627–2630, 10.1007/BF00540680.

[67] F. Yang , Y. Chen , W. Hai , et al., “Research Progress on High‐Thermal‐Conductivity Silicon Carbide Ceramics,” Ceramics International 51 (2025): 4095–4109, 10.1016/j.ceramint.2024.11.408.

[68] H. Tao , X. Qin , J. Sui , et al., “Textured Silicon Nitride Ceramics with Enhanced Properties Fabricated by High Pressure Sintering,” Ceramics International 51 (2025): 33324–33331, 10.1016/j.ceramint.2025.05.065.

[69] J. S. Haggerty and A. Lightfoot , “Opportunities for Enhancing the Thermal Conductivities of SiC and Si_3_N_4_ Ceramics through Improved Processing,” in Proceedings of the 19th Annual Conference on Composites, Advanced Ceramics, Materials, and Structures—A: Ceramic Engineering and Science Proceedings, Ltd, (John Wiley & Sons, 1995), 10.1002/9780470314715.ch52.

[70] Y. Zhou , H. Hyuga , D. Kusano , Y. Yoshizawa , and K. Hirao , “A Tough Silicon Nitride Ceramic with High Thermal Conductivity,” Advanced Materials 23 (2011): 4563–4567, 10.1002/adma.201102462. 21901768

[71] Y. Fukuda , K. Harada , M. Yonetsu , et al., “Relation Between Crystal Structure and Lattice Oxygen Content of Sintered Reaction‐Bonded Silicon Nitride,” Journal of the American Ceramic Society 104 (2021): 6563–6571, 10.1111/jace.18023.

[72] K. Hirao , Y. Zhou , H. Hyuga , T. Ohji , and D. Kusano , “High Thermal Conductivity Silicon Nitride Ceramics,” Journal of the Korean Ceramic Society 49 (2012): 380–384, 10.4191/kcers.2012.49.4.380.

[73] H. Yokota , H. Abe , and M. Ibukiyama , “Effect of Lattice Defects on the Thermal Conductivity of β‐Si_3_N_4_ ,” Journal of the European Ceramic Society 23 (2003): 1751–1759, 10.1016/S0955-2219(02)00374-6.

[74] A. Kuwabara , K. Matsunaga , and I. Tanaka , “Lattice Dynamics and Thermodynamical Properties of Silicon Nitride Polymorphs,” Physical Review B 78 (2008): 064104, 10.1103/PhysRevB.78.064104.

[75] J.‐S. Lee , J.‐H. Mun , B.‐D. Han , and H.‐D. Kim , “Effect of β‐Si_3_N_4_ Seed Particles on the Property of Sintered Reaction‐Bonded Silicon Nitride,” Ceramics International 29 (2003): 897–905, 10.1016/S0272-8842(03)00034-8.

[76] H. Ding , Y. Hu , X. Li , Z. Zhao , and H. Ji , “Microstructure, Mechanical Properties and Sintering Mechanism of Pressureless‐Sintered Porous Si_3_N_4_ Ceramics with YbF_3_‐MgF_2_ Composite Sintering Aids,” Ceramics International 46 (2020): 2558–2564, 10.1016/j.ceramint.2019.09.114.

[77] Y. Nakashima , R. Furushima , Y. Zhou , K. Hirao , T. Ohji , and M. Fukushima , “Deciphering the Effect of Grain Boundary Characteristics on Fracture Toughness of Silicon Nitride Ceramics Through a CNN Regression Model,” Ceramics International 50 (2024): 6680–6686, 10.1016/j.ceramint.2023.12.006.

[78] X. W. Zhu , Y. Sakka , T. S. Suzuki , T. Uchikoshi , and S. Kikkawa , “The *c*‐Axis Texturing of Seeded Si_3_N_4_ with β‐Si_3_N_4_ Whiskers by Slip Casting in a Rotating Magnetic Field,” Acta Materialia 58 (2010): 146–161, 10.1016/j.actamat.2009.08.064.

[79] W. Wang , D. Yao , H. Liang , et al., “Enhanced Thermal Conductivity in Si_3_N_4_ Ceramics Prepared by Using ZrH_2_ as an Oxygen Getter,” Journal of Alloys and Compounds 855 (2021): 157451, 10.1016/j.jallcom.2020.157451.

[80] H. Xie , P. Liu , H. Liang , et al., “A Cost‐Effective Strategy for Fabricating High Thermal Conductivity Si_3_N_4_ Ceramics with Well‐Balanced Properties,” Journal of the European Ceramic Society 46 (2026): 117937, 10.1016/j.jeurceramsoc.2025.117937.

[81] B. Fan , T. Wang , D. Zhao , et al., “Tailored Grain Growth Strategy for Si_3_N_4_ Ceramics With Enhanced Thermal and Mechanical Performance,” Journal of Alloys and Compounds 1048 (2025): 185175, 10.1016/j.jallcom.2025.185175.

[82] X. Chen , Y. Li , Y. Qi , et al., “Preparation of Si_3_N_4_ Ceramics with High Thermal Conductivity and Mechanical Properties Using Novel Gd_3_Si_2_C_2_ as a Sintering Aid,” Ceramics International 51 (2025): 9931–9938, 10.1016/j.ceramint.2024.12.425.

[83] Y. Liu , R. Liu , Y. Zheng , et al., “Densification, Microstructure, Thermal and Mechanical Properties of Si_3_N_4_ Ceramics: Effect of Y_2_Si_4_N_6_C and MgSiN_2_ Content,” Ceramics International 50 (2024): 38507–38513, 10.1016/j.ceramint.2024.07.215.

[84] Y. Liu , R. Liu , Y. Zheng , et al., “Effect of the Ratio of Y_2_O_3_ and MgSiN_2_ Sintering Additives on the Microstructure, Thermal and Mechanical Properties of Si_3_N_4_ Ceramics,” Ceramics International 49 (2023): 36490–36496, 10.1016/j.ceramint.2023.08.332.

[85] S. Fu , Z. Yang , H. Li , L. Wang , Y. Li , and J. Li , “Effects of Gd_2_O_3_ and MgSiN_2_ Sintering Additives on the Thermal Conductivity and Mechanical Properties of Si_3_N_4_ Ceramics,” International Journal of Applied Ceramic Technology 20 (2023): 1855–1864, 10.1111/ijac.14279.

[86] M. Huang , Y. Huang , J. Ou , Y. Wu , J. Wang , and S. Wu , “Effect of a New Nonoxide Additive, Y_3_Si_2_C_2_, on the Thermal Conductivity and Mechanical Properties of Si_3_N_4_ Ceramics,” International Journal of Applied Ceramic Technology 19 (2022): 3403–3409, 10.1111/ijac.14132.

[87] W. Wang , D. Yao , H. Liang , et al., “Improved Thermal Conductivity of β‐Si_3_N_4_ Ceramics Through the Modification of the Liquid Phase by Using GdH_2_ as a Sintering Additive,” Ceramics International 47 (2021): 5631–5638, 10.1016/j.ceramint.2020.10.148.

[88] W. Wang , D. Yao , H. Chen , et al., “ZrSi_2_–MgO as Novel Additives for High Thermal Conductivity of β‐Si_3_N_4_ Ceramics,” Journal of the American Ceramic Society 103 (2020): 2090–2100, 10.1111/jace.16902.

[89] K. Shimada and J. Tatami , “Effect of Coarse‐Si‐Powder Addition on the Thermal and Mechanical Properties of Sintered Reaction‐Bonded Silicon Nitride,” Ceramics International 51 (2025): 44942–44951, 10.1016/j.ceramint.2025.07.215.

[90] Y. Nakashima , Y. Zhou , K. Tanabe , et al., “Effects of Nitridation Temperature on Properties of Sintered Reaction‐Bonded Silicon Nitride,” International Journal of Applied Ceramic Technology 20 (2023): 1071–1080, 10.1111/ijac.14163.

[91] H.‐M. Oh and H.‐K. Lee , “Controlling the Width of Particle Size Distribution of Si Powder and Properties of Sintered Reaction‐Bonded Silicon Nitride (SRBSN) Ceramics with High Thermal Conductivity,” Ceramics International 46 (2020): 12517–12524, 10.1016/j.ceramint.2020.02.014.

[92] Y. Zhou , X. Zhu , K. Hirao , and Z. Lences , “Sintered Reaction‐Bonded Silicon Nitride with High Thermal Conductivity and High Strength,” International Journal of Applied Ceramic Technology 5 (2008): 119–126, 10.1111/j.1744-7402.2008.02187.x.

[93] X. Zhu , Y. Sakka , Y. Zhou , and K. Hirao , “Processing and Properties of Sintered Reaction‐Bonded Silicon Nitride with Y_2_O_3_–MgSiN_2_: Effects of Si Powder and Li_2_O Addition,” Acta Materialia 55 (2007): 5581–5591, 10.1016/j.actamat.2007.06.014.

[94] F. Hu , Z. Wang , and Z. Xie , “Enhancing Si_3_N_4_ Ceramic Performance by Microstructure Control through Sintering Additive Optimization and Grain Orientation Control,” Ceramics International 51 (2025): 53442–53450, 10.1016/j.ceramint.2025.09.092.

[95] H. Liang , W. Wang , K. Zuo , et al., “Effect of LaB_6_ Addition on Mechanical Properties and Thermal Conductivity of Silicon Nitride Ceramics,” Ceramics International 46 (2020): 17776–17783, 10.1016/j.ceramint.2020.04.083.

[96] H. Liang , W. Wang , K. Zuo , et al., “YB_2_C_2_: A New Additive for Fabricating Si_3_N_4_ Ceramics with Superior Mechanical Properties and Medium Thermal Conductivity,” Ceramics International 46 (2020): 5239–5243, 10.1016/j.ceramint.2019.10.272.

[97] H. Liang , Y. Zeng , K. Zuo , Y. Xia , D. Yao , and J. Yin , “Mechanical Properties and Thermal Conductivity of Si_3_N_4_ Ceramics with YF_3_ and MgO as Sintering Additives,” Ceramics International 42 (2016): 15679–15686, 10.1016/j.ceramint.2016.07.024.

[98] C. Yang , Q. Liu , B. Zhang , et al., “Effect of MgF_2_ Addition on Mechanical Properties and Thermal Conductivity of Silicon Nitride Ceramics,” Ceramics International 45 (2019): 12757–12763, 10.1016/j.ceramint.2019.03.183.

[99] X. Lv , J. Huang , X. Dong , Q. Yan , and C. Ge , “Influence of α‐Si_3_N_4_ Coarse Powder on Densification, Microstructure, Mechanical Properties, and Thermal Behavior of Silicon Nitride Ceramics,” Ceramics International 49 (2023): 21815–21824, 10.1016/j.ceramint.2023.04.003.

[100] Z. Guo , Q. Qin , J. Huang , et al., “Effect of Non‐Oxide Additives on the Phase Composition, Microstructure, Mechanical Properties and Thermal Conductivity of Si_3_N_4_ Fabricated by Spark Plasma Sintering and Annealing,” Ceramics International 51 (2025): 57790–57799, 10.1016/j.ceramint.2025.09.479.

[101] Y. Duan , J. Zhang , X. Li , H. Bai , P. Sajgalik , and D. Jiang , “High Thermal Conductivity Silicon Nitride Ceramics Prepared by Pressureless Sintering with Ternary Sintering Additives,” International Journal of Applied Ceramic Technology 16 (2019): 1399–1406, 10.1111/ijac.13220.

[102] C. Luo , Y. Zhang , and T. Deng , “Pressureless Sintering of High Performance Silicon Nitride Ceramics at 1620°C,” Ceramics International 47 (2021): 29371–29378, 10.1016/j.ceramint.2021.07.104.

[103] D. Kusano , S. Adachi , G. Tanabe , H. Hyuga , Y. Zhou , and K. Hirao , “Effects of Impurity Oxygen Content in Raw Si Powder on Thermal and Mechanical Properties of Sintered Reaction‐Bonded Silicon Nitrides,” International Journal of Applied Ceramic Technology 9 (2012): 229–238, 10.1111/j.1744-7402.2011.02618.x.

[104] P. D. Ramesh , R. Oberacker , and M. J. Hoffmann , “Preparation of β‐Silicon Nitride Seeds for Self‐Reinforced Silicon Nitride Ceramics,” Journal of the American Ceramic Society 82 (1999): 1608–1610, 10.1111/j.1151-2916.1999.tb01969.x.

[105] H.‐H. Lu and J.‐L. Huang , “Microstructure in Silicon Nitride Containing β‐Phase Seeding: Part I,” Journal of Materials Research 14 (1999): 2966–2973, 10.1557/JMR.1999.0397.

[106] P. Dehghani , S. S. S. Afghahi , F. Soleimani , P. Dehghani , S. S. S. Afghahi , and F. Soleimani , “Hot Isostatic Pressing (HIP) in Advanced Ceramics Production,” in Advanced Ceramic Materials—Emerging Technologies (IntechOpen, 2025), 10.5772/intechopen.1007176.

[107] L. Cao , Z. Wang , Z. Yin , K. Liu , and J. Yuan , “Investigation on Mechanical Properties and Microstructure of Silicon Nitride Ceramics Fabricated by Spark Plasma Sintering,” Materials Science and Engineering: A 731 (2018): 595–602, 10.1016/j.msea.2018.06.093.

[108] H. Miyazaki , Y. Zhou , S. Iwakiri , et al., “Improved Resistance to Thermal Fatigue of Active Metal Brazing Substrates for Silicon Carbide Power Modules Using Tough Silicon Nitrides with High Thermal Conductivity,” Ceramics International 44 (2018): 8870–8876, 10.1016/j.ceramint.2018.02.072.

[109] J. Li , Q. Jiang , Z. Pan , D. Lv , and S. Wu , “Fabrication of Silicon Nitride with High Thermal Conductivity and Flexural Strength by Hot‐Pressing Flowing Sintering,” International Journal of Applied Ceramic Technology 21 (2024): 2841–2849, 10.1111/ijac.14741.

[110] X. Zhu , S. Y. W , Y. Zhou , K. Hirao , and K. Itatani , “A Strategy for Fabricating Textured Silicon Nitride with Enhanced Thermal Conductivity,” Journal of the European Ceramic Society 34 (2014): 2585–2589, 10.1016/j.jeurceramsoc.2014.01.025.

[111] X. Zhu and Y. Sakka , “Textured Silicon Nitride: Processing and Anisotropic Properties,” Science and Technology of Advanced Materials 9 (2008): 033001, 10.1088/1468-6996/9/3/033001. 27877995 PMC5099652

[112] Y.‐P. Zeng , J.‐F. Yang , N. Kondo , T. Ohji , H. Kita , and S. Kanzaki , “Fracture Energies of Tape‐Cast Silicon Nitride with β‐Si_3_N_4_ Seed Addition,” Journal of the American Ceramic Society 88 (2005): 1622–1624, 10.1111/j.1551-2916.2005.00242.x.

[113] S.‐J. Tang , Z.‐H. Li , W.‐M. Guo , J.‐J. Yu , S.‐K. Sun , and H.‐T. Lin , “Fabrication of One‐Dimensional Textured Si_3_N_4_‐Based Ceramics with High Hardness and Toughness by Low Temperature Hot Extrusion,” Ceramics International 50 (2024): 41975–41981, 10.1016/j.ceramint.2024.08.022.

[114] Z.‐H. Li , S.‐Y. Tong , Y.‐X. Wang , J.‐J. Yu , W.‐M. Guo , and H.‐T. Lin , “Si_3_N_4_ Ceramics with Fine‐Grained Bimodal Microstructure and Excellent Mechanical Properties Prepared by Two‐Step Spark Plasma Sintering,” Journal of the European Ceramic Society 45 (2025): 117331, 10.1016/j.jeurceramsoc.2025.117331.

[115] R. Geng , T. Fan , P. Yang , et al., “High‐Performance Si_3_N_4_ Ceramics Prepared by Gas Pressure Sintering with Y_2_O_3_‐MgO‐MgSiN_2_ Ternary Additives,” Ceramics International 51 (2025): 43978–43985, 10.1016/j.ceramint.2025.07.128.

[116] P. Aiyi , L. Junguo , C. Yang , L. Meijuan , and S. Qiang , “Low‐Temperature Fabrication of Si_3_N_4_ Ceramics with High Thermal Conductivities using a Single Mg_2_Si Sintering Additive,” Ceramics International 49 (2023): 39473–39478, 10.1016/j.ceramint.2023.09.293.

[117] S. Liao , Y. Zhuang , J. Wang , et al., “Synergistic Effect of Binary Fluoride Sintering Additives on the Properties of Silicon Nitride Ceramics,” Ceramics International 48 (2022): 21832–21845, 10.1016/j.ceramint.2022.04.167.

[118] Y. Shi , Q. He , A. Wang , H. Wang , W. Wang , and Z. Fu , “New Perspective on the Texture Evolution Mechanism of Si_3_N_4_ Ceramics: Effect of Additive Content,” Ceramics International 49 (2023): 22602–22607, 10.1016/j.ceramint.2023.01.136.

[119] K. Shimada and J. Tatami , “Grain and Grain Boundary Strength of Silicon Nitride Ceramics with Different Thermal Conductivities and Microstructures,” Ceramics International 51 (2025): 24306–24313, 10.1016/j.ceramint.2025.03.119.

[120] W. Wang , Y. Liu , Y. Pan , et al., “The Effects of Silicon Additive Content on Thermal Conductivity and Mechanical Properties of Si_3_N_4_ Ceramics,” Journal of the American Ceramic Society 108 (2025): 20534, 10.1111/jace.20534.

[121] Q. Zhang , W. Wang , Z. Zhang , et al., “Enhancing Fracture Toughness of Silicon Nitride Ceramics by Addition of β‐Si_3_N_4_ Whisker and MXene,” Ceramics International 50 (2024): 35695–35705, 10.1016/j.ceramint.2024.06.387.

[122] H. Xiong , B. Li , X. Xi , and Q. Shan , “Preparation of Graded Silicon Nitride Ceramics with High Mechanical Performance Using β‐Si_3_N_4_ Seeds,” Ceramics International 49 (2023): 36528–36535, 10.1016/j.ceramint.2023.08.336.

[123] J. Zhang , G. Liu , W. Cui , et al., “Plastic Deformation in Silicon Nitride Ceramics via Bond Switching at Coherent Interfaces,” Science 378 (2022): 371–376, 10.1126/science.abq7490. 36302007

[124] R. Furushima , Y. Nakashima , Y. Zhou , K. Hirao , T. Ohji , and M. Fukushima , “Thermal Conductivity Prediction of Sintered Reaction Bonded Silicon Nitride Ceramics Using a Machine Learning Approach Based on Process Conditions,” Ceramics International 50 (2024): 8520–8526, 10.1016/j.ceramint.2023.12.231.

[125] R. Furushima , Y. Nakashima , Y. Zhou , K. Hirao , T. Ohji , and M. Fukushima , “Multilayer Artificial Intelligence for Thermal‐Conductivity Prediction of Silicon Nitride Ceramics From Powder Processing Conditions and Predicted Densities,” Ceramics International 50 (2024): 24008–24015, 10.1016/j.ceramint.2024.04.132.

[126] R. Furushima , Y. Nakashima , Y. Maruyama , K. Hirao , T. Ohji , and M. Fukushima , “Artificial Intelligence‐Based Determination of Fracture Toughness and Bending Strength of Silicon Nitride Ceramics,” Journal of the American Ceramic Society 106 (2023): 4944–4954, 10.1111/jace.19147.

[127] D. Milardovich , C. Wilhelmer , D. Waldhoer , L. Cvitkovich , G. Sivaraman , and T. Grasser , “Machine Learning Interatomic Potential for Silicon‐Nitride (Si_3_N_4_) by Active Learning,” Journal of Chemical Physics 158 (2023): 194802, 10.1063/5.0146753. 37184017

[128] W. Guo , F. Wang , Z. Zhang , Z. Liu , W. Zhang , and S. Bai , “Accelerated Design of High Thermal Conductivity Si_3_N_4_ Ceramics Based on Machine Learning,” Ceramics International 51 (2025): 33145–33154, 10.1016/j.ceramint.2025.05.047.

[129] A. Wang , W. Xiong , J. Zhou , K. Qu , and H. He , “A New Path to Intelligent Quantitative Prediction of Ceramic Strength: Machine Vision Combined with Machine Learning,” Journal of the American Ceramic Society 108 (2025): 70025, 10.1111/jace.70025.

[130] A. Sharma , T. Mukhopadhyay , S. M. Rangappa , S. Siengchin , and V. Kushvaha , “Advances in Computational Intelligence of Polymer Composite Materials: Machine Learning Assisted Modeling, Analysis and Design,” Archives of Computational Methods in Engineering (2022): 3341–3385, 10.1007/s11831-021-09700-9.

[131] R. Furushima , Y. Nakashima , Y. Maruyama , et al., “Microstructural Basis of AI Predictions for Material Properties: A Case Study of Silicon Nitride Ceramics Using *t*‐SNE,” Journal of the American Ceramic Society 108 (2025): 20173, 10.1111/jace.20173.

[132] C. C. Price , Y. Li , G. Zhou , et al., “Predicting and Accelerating Nanomaterial Synthesis Using Machine Learning Featurization,” Nano Letters 24 (2024): 14862–14867, 10.1021/acs.nanolett.4c04500. 39529538

[133] Y. Tu , B. Liu , G. Yao , et al., “A Review of Advanced Thermal Interface Materials with Oriented Structures for Electronic Devices,” Electronics 13 (2024): 4287, 10.3390/electronics13214287.

[134] C. L. Gan , M.‐H. Chung , L.‐F. Lin , C.‐Y. Huang , and H. Takiar , “Evolution of Epoxy Molding Compounds and Future Carbon Materials for Thermal and Mechanical Stress Management in Memory Device Packaging: A Critical Review,” Journal of Materials Science: Materials in Electronics 34 (2023): 2011, 10.1007/s10854-023-11388-5.

[135] A. K. Singh , B. P. Panda , S. Mohanty , S. K. Nayak , and M. K. Gupta , “Recent Developments on Epoxy‐Based Thermally Conductive Adhesives (TCA): A Review,” Polymer‐Plastics Technology and Engineering 57 (2018): 903–934, 10.1080/03602559.2017.1354253.

[136] M.‐H. Zhou , G.‐Z. Yin , and S. González Prolongo , “Review of Thermal Conductivity in Epoxy Thermosets and Composites: Mechanisms, Parameters, and Filler Influences,” Advanced Industrial and Engineering Polymer Research 7 (2024): 295–308, 10.1016/j.aiepr.2023.08.003.

[137] G. Chang , S. Zhang , K. Chen , et al., “Achieving Excellent Thermal Transport in Diamond/Cu Composites by Breaking Bonding Strength‐Heat Transfer Trade‐off Dilemma at the Interface,” Composites Part B: Engineering 289 (2025): 111925, 10.1016/j.compositesb.2024.111925.

[138] L. Wang , G. Bai , N. Li , et al., “Unveiling Interfacial Structure and Improving Thermal Conductivity of Cu/Diamond Composites Reinforced with Zr‐Coated Diamond Particles,” Vacuum 202 (2022): 111133, 10.1016/j.vacuum.2022.111133.

[139] Y. Zhang , Z. Wang , N. Li , et al., “Interfacial Thermal Conductance Between Cu and Diamond with Interconnected W−W_2_C Interlayer,” ACS Applied Materials & Interfaces 14 (2022): 35215–35228, 10.1021/acsami.2c07190. 35878880

[140] M. J. Meziani , W.‐L. Song , P. Wang , et al., “Boron Nitride Nanomaterials for Thermal Management Applications,” Chemphyschem 16 (2015): 1339–1346, 10.1002/cphc.201402814. 25652360

[141] H. Yang , H. Fang , H. Yu , et al., “Low Temperature Self‐Densification of High Strength Bulk Hexagonal Boron Nitride,” Nature Communications 10 (2019): 854, 10.1038/s41467-019-08580-9. PMC6382832 30787275

[142] T. F. Retajczyk Jr and A. K. Sinha , “Elastic Stiffness and Thermal Expansion Coefficient of BN Films,” Applied Physics Letters 36 (1980): 161–163, 10.1063/1.91415.

[143] J. A. Cuenca , S. Mandal , D. J. Morgan , M. Snowball , A. Porch , and O. A. Williams , “Dielectric Spectroscopy of Hydrogen‐Treated Hexagonal Boron Nitride Ceramics,” ACS Applied Electronic Materials 2 (2020): 1193–1202, 10.1021/acsaelm.9b00767.

[144] Y. Ji , C. Pan , M. Zhang , et al., “Boron Nitride as Two Dimensional Dielectric: Reliability and Dielectric Breakdown,” Applied Physics Letters 108 (2016): 012905, 10.1063/1.4939131.

[145] D. G. Onn , A. Witek , Y. Z. Qiu , T. R. Anthony , and W. F. Banholzer , “Some Aspects of the Thermal Conductivity of Isotopically Enriched Diamond Single Crystals,” Physical Review Letters 68 (1992): 2806–2809, 10.1103/PhysRevLett.68.2806. 10045497

[146] J. R. Olson , R. O. Pohl , J. W. Vandersande , A. Zoltan , T. R. Anthony , and W. F. Banholzer , “Thermal Conductivity of Diamond Between 170 and 1200 K and the Isotope Effect,” Physical Review B 47 (1993): 14850–14856, 10.1103/PhysRevB.47.14850. 10005859

[147] L. Wei , P. K. Kuo , R. L. Thomas , T. R. Anthony , and W. F. Banholzer , “Thermal Conductivity of Isotopically Modified Single Crystal Diamond,” Physical Review Letters 70 (1993): 3764–3767, 10.1103/PhysRevLett.70.3764. 10053956

[148] P. Jacobson and S. Stoupin , “Thermal Expansion Coefficient of Diamond in a Wide Temperature Range,” Diamond and Related Materials 97 (2019): 107469, 10.1016/j.diamond.2019.107469.

[149] S. Bhagavantam and D. A. A. S. Narayana Rao , “Dielectric Constant of Diamond,” Nature 161 (1948): 729, 10.1038/161729a0.

[150] C. J. H. Wort and R. S. Balmer , “Diamond as an Electronic Material,” Materials Today 11 (2008): 22–28, 10.1016/S1369-7021(07)70349-8.

[151] S. Zhang , M. Ding , L. Wang , W. Ge , and W. Yan , “Laser Powder Bed Fusion of Diamond/N6 MMCs Enabled by Ni‐Ti Coated Diamond Particles,” Materials & Design 217 (2022): 110635, 10.1016/j.matdes.2022.110635.

[152] H. Li , C. Wang , W. Ding , et al., “Microstructure Evolution of Diamond with Molybdenum Coating and Thermal Conductivity of Diamond/Copper Composites Fabricated by Spark Plasma Sintering,” Journal of Materials Science: Materials in Electronics 33 (2022): 15369–15384, 10.1007/s10854-022-08441-0.

[153] Z. Jiao , L. Zhang , Z. Deng , et al., “Highly Conductive Diamond Skeleton Reinforced Cu‐Matrix Composites for High‐Efficiency Thermal Management,” Applied Surface Science 645 (2024): 158829, 10.1016/j.apsusc.2023.158829.

[154] M. Malakoutian , D. Field , H. N. E , et al., “Record‐Low Thermal Boundary Resistance Between Diamond and GaN‐on‐SiC for Enabling Radiofrequency Device Cooling,” ACS Applied Materials & Interfaces 13 (2021): 60553–60560, 10.1021/acsami.1c13833. 34875169

[155] J. Sang , W. Yang , H. Chen , et al., “Metallurgical Behaviors of Tungsten Coatings at Diamond/Al Interface and Its Influence on Thermal Conductivity and Stability of Diamond/Al Composites,” Journal of Alloys and Compounds 1021 (2025): 179539, 10.1016/j.jallcom.2025.179539.

[156] N. Li , J. Hao , Y. Zhang , et al., “Thermal Conductivity Stability of Interfacial in Situ Al_4_C_3_ Engineered Diamond/Al Composites Subjected to Thermal Cycling,” Materials 15 (2022): 6640, 10.3390/ma15196640. 36233982 PMC9571598

[157] C. Zeng , J. Shen , M. Gong , and H. Chen , “Enhanced Thermal Conductivity in TiC/Diamond or Cr_3_C_2_/Diamond Particles Modified Bi‐In‐Sn Compounds,” Journal of Materials Science: Materials in Electronics 32 (2021): 13205–13219, 10.1007/s10854-021-05859-w.

[158] N. Si , Q. Yan , and H. Zhang , “Surface‐Metallized Diamond/Liquid Metal Composites Through Diamond Size Engineering as High‐Performance Thermal Interface Materials,” Surfaces and Interfaces 60 (2025), 105989, 10.1016/j.surfin.2025.105989.

[159] H. Wang , F. Huang , W. Qin , et al., “Effect of Diamond Morphology on Construction of Thermal Conduction Path in Flexible Thermal Interface Materials,” Journal of Materials Engineering and Performance 33 (2024): 11104–11112, 10.1007/s11665-023-08724-5.

[160] Y. Li , X. Liao , X. Guo , et al., “Improving Thermal Conductivity of Epoxy‐Based Composites by Diamond‐Graphene Binary Fillers,” Diamond and Related Materials 126 (2022): 109141, 10.1016/j.diamond.2022.109141.

[161] T. Yoshitomi , T. Matsumoto , and T. Nishino , “Highly Thermally Conductive Nanocomposites Prepared by the Ice‐Templating Alignment of Nanodiamonds in the Thickness Direction,” ACS Applied Polymer Materials 5 (2023): 8349–8358, 10.1021/acsapm.3c01503.

[162] D. Wu , C. Wang , X. Hu , and W. Chen , “Fabrication and Characterization of Highly Thermal Conductive Si_3_N_4_/Diamond Composite Materials,” Materials & Design 225 (2023): 111482, 10.1016/j.matdes.2022.111482.

[163] D. Wu , H. Ding , Z.‐Q. Fan , P.‐Z. Jia , H.‐Q. Xie , and X.‐K. Chen , “High Interfacial Thermal Conductance Across Heterogeneous GaN/Graphene Interface,” Applied Surface Science 581 (2022): 152344, 10.1016/j.apsusc.2021.152344.

[164] I. Meric , M. Y. Han , A. F. Young , B. Ozyilmaz , P. Kim , and K. L. Shepard , “Current Saturation in Zero‐Bandgap, Top‐Gated Graphene Field‐Effect Transistors,” Nature Nanotechnology 3 (2008): 654–659, 10.1038/nnano.2008.268. 18989330

[165] S. Wieghold , J. Li , P. Simon , et al., “Photoresponse of Supramolecular Self‐Assembled Networks on Graphene–Diamond Interfaces,” Nature Communications 7 (2016): 10700, 10.1038/ncomms10700. PMC4773422 26911248

[166] A. Nie , Z. Zhao , B. Xu , and Y. Tian , “Microstructure Engineering in Diamond‐Based Materials,” Nature Materials 24 (2025): 1172–1185, 10.1038/s41563-025-02168-z. 40175725

[167] X. Li , D. Jin , S. Ding , and G. Yang , “High Interfacial Thermal Conductance in Graphite‐Diamond Hybrids,” Journal of Physical Chemistry C 128 (2024): 14500–14506, 10.1021/acs.jpcc.4c03868.

[168] H. Zhang , Q. He , H. Yu , M. Qin , Y. Feng , and W. Feng , “A Bioinspired Polymer‐Based Composite Displaying Both Strong Adhesion and Anisotropic Thermal Conductivity,” Advanced Functional Materials 33 (2023): 2211985, 10.1002/adfm.202211985.

[169] M. Li , Y. Sun , D. Feng , K. Ruan , X. Liu , and J. Gu , “Thermally Conductive Polyvinyl Alcohol Composite Films via Introducing Hetero‐Structured MXene@Silver Fillers,” Nano Research 16 (2023): 7820–7828, 10.1007/s12274-023-5594-1.

[170] R. Kang , Z. Zhang , L. Guo , et al., “Enhanced Thermal Conductivity of Epoxy Composites Filled with 2D Transition Metal Carbides (MXenes) with Ultralow Loading,” Scientific Reports 9 (2019): 9135, 10.1038/s41598-019-45664-4. 31235757 PMC6591414

[171] H. Yu , Y. Feng , C. Chen , et al., “Highly Thermally Conductive Adhesion Elastomer Enhanced by Vertically Aligned Folded Graphene,” Advanced Science 9 (2022): 2201331, 10.1002/advs.202201331. 36251921 PMC9685443

[172] Z. Lin , Y. Liu , S. Raghavan , K. Moon , S. K. Sitaraman , and C. Wong , “Magnetic Alignment of Hexagonal Boron Nitride Platelets in Polymer Matrix: Toward High Performance Anisotropic Polymer Composites for Electronic Encapsulation,” ACS Applied Materials & Interfaces 5 (2013): 7633–7640, 10.1021/am401939z. 23815609

[173] J. Yuan , X. Qian , Z. Meng , B. Yang , and Z.‐Q. Liu , “Highly Thermally Conducting Polymer‐Based Films with Magnetic Field‐Assisted Vertically Aligned Hexagonal Boron Nitride for Flexible Electronic Encapsulation,” ACS Applied Materials & Interfaces 11 (2019): 17915–17924, 10.1021/acsami.9b06062. 31026136

[174] X. Ma , H. Zhang , Y. Guo , et al., “Enhancing Thermal Conductivity in Polysiloxane Composites through Synergistic Design of Liquid Crystals and Boron Nitride Nanosheets,” Journal of Materials Science & Technology 231 (2025): 54–61, 10.1016/j.jmst.2025.01.004.

[175] S. Xu , S. Wang , Z. Chen , et al., “Electric‐Field‐Assisted Growth of Vertical Graphene Arrays and the Application in Thermal Interface Materials,” Advanced Functional Materials 30 (2020): 2003302, 10.1002/adfm.202003302.

[176] D. Du , Y. Hao , and Y. He , “High Frequency Electric‐Field‐Assisted Preparation of BN/Epoxy Resin Composites with Excellent Electrical, Thermal, and Mechanical Properties,” Polymers 17 (2025): 1429, 10.3390/polym17111429. 40508672 PMC12157035

[177] G. Czel , A. Sycheva , and D. Janovszky , “Effect of Different Fillers on Thermal Conductivity, Tribological Properties of Polyamide 6,” Scientific Reports 13 (2023): 845, 10.1038/s41598-023-27740-y. 36646774 PMC9842670

[178] S. Jasmee , G. Omar , S. S. C. Othaman , N. A. Masripan , and H. A. Hamid , “Interface Thermal Resistance and Thermal Conductivity of Polymer Composites at Different Types, Shapes, and Sizes of Fillers: A Review,” Polymer Composites 42 (2021): 2629–2652, 10.1002/pc.26029.

[179] X. Wang , X. Niu , X. Wang , X. Qiu , and L. Wang , “Effects of Filler Distribution and Interface Thermal Resistance on the Thermal Conductivity of Composites Filling with Complex Shaped Fillers,” International Journal of Thermal Sciences 160 (2021): 106678, 10.1016/j.ijthermalsci.2020.106678.

[180] F. Liu , R. Mao , Z. Liu , J. Du , and P. Gao , “Probing Phonon Transport Dynamics Across an Interface by Electron Microscopy,” Nature 642 (2025): 941–946, 10.1038/s41586-025-09108-6. 40500446 PMC12738285

[181] S. Hu , C. Zhao , and X. Gu , “Phonon Non‐Equilibrium Effects on Interface Thermal Resistance Between Graphene and Substrates,” International Journal of Thermal Sciences 196 (2024): 108725, 10.1016/j.ijthermalsci.2023.108725.

[182] S. Shan , Z. Zhang , S. Volz , and J. Chen , “Phonon Mode at Interface and Its Impact on Interfacial Thermal Transport,” Journal of Physics: Condensed Matter 36 (2024): 423001, 10.1088/1361-648X/ad5fd7. 38968932

[183] M. M. Islam and L. Liu , “Enhancing Interfacial Thermal Transport by Grafting H‐Bonded Polymer Chains: The Role of Chain Morphology,” Applied Surface Science 697 (2025): 163009, 10.1016/j.apsusc.2025.163009.

[184] M. D. Losego , M. E. Grady , N. R. Sottos , D. G. Cahill , and P. V. Braun , “Effects of Chemical Bonding on Heat Transport Across Interfaces,” Nature Materials 11 (2012): 502–506, 10.1038/nmat3303. 22522593

[185] Y. Liu , L. Qiu , Z. Wang , H. Li , and Y. Feng , “Enhancing Interfacial Thermal Transport Efficiently in Diamond/Graphene Heterostructure by Involving Vacancy Defects,” Composites Part A: Applied Science and Manufacturing 178 (2024): 108008, 10.1016/j.compositesa.2024.108008.

[186] W. Miao and M. Wang , “Importance of Electron‐Phonon Coupling in Thermal Transport in Metal/Semiconductor Multilayer Films,” International Journal of Heat and Mass Transfer 200 (2023): 123538, 10.1016/j.ijheatmasstransfer.2022.123538.

[187] J. Chen , G. Chen , Z. Wang , and D. Tang , “Modulation of Localized Phonon Thermal Transport at GaN/Al* _x_ *Ga_1‐_ * _x_ *N Heterointerface: Polar Surface, Doping, and Compressive Strain,” International Journal of Heat and Mass Transfer 220 (2024): 124945, 10.1016/j.ijheatmasstransfer.2023.124945.

[188] E. A. Chagarov , M. S. Kavrik , Z. Fang , W. Tsai , and A. C. Kummel , “Density‐Functional Theory Molecular Dynamics Simulations of a‐HfO_2_/a‐SiO_2_/SiGe and a‐HfO_2_/a‐SiO_2_/Ge with a‐SiO_2_ and a‐SiO Suboxide Interfacial Layers,” Applied Surface Science 443 (2018): 644–654, 10.1016/j.apsusc.2018.02.041.

[189] Q. Chen , K. Yang , Y. Feng , et al., “Recent Advances in Thermal‐Conductive Insulating Polymer Composites with Various Fillers,” Composites Part A: Applied Science and Manufacturing 178 (2024): 107998, 10.1016/j.compositesa.2023.107998.

[190] Y. P. Mamunya , V. V. Davydenko , P. Pissis , and E. V. Lebedev , “Electrical and Thermal Conductivity of Polymers Filled with Metal Powders,” European Polymer Journal (2002): 1887–1897, 10.1016/S0014-3057(02)00064-2.

[191] H. Chen , V. V. Ginzburg , J. Yang , et al., “Thermal Conductivity of Polymer‐Based Composites: Fundamentals and Applications,” Progress in Polymer Science 59 (2016): 41–85, 10.1016/j.progpolymsci.2016.03.001.

[192] T. Ji , Y. Feng , M. Qin , et al., “Thermal Conductive and Flexible Silastic Composite Based on a Hierarchical Framework of Aligned Carbon Fibers‐Carbon Nanotubes,” Carbon 131 (2018): 149–159, 10.1016/j.carbon.2018.02.002.

[193] C. Huang , X. Qian , and R. Yang , “Thermal Conductivity of Polymers and Polymer Nanocomposites,” Materials Science and Engineering: R: Reports 132 (2018): 1–22, 10.1016/j.mser.2018.06.002.

[194] E. T. Swartz and R. O. Pohl , “Thermal Boundary Resistance,” Reviews of Modern Physics 61 (1989): 605–668, 10.1103/RevModPhys.61.605.

[195] M. Hu , P. Keblinski , and P. K. Schelling , “Kapitza Conductance of Silicon–Amorphous Polyethylene Interfaces by Molecular Dynamics Simulations,” Physical Review B 79 (2009): 104305, 10.1103/PhysRevB.79.104305.

[196] T. Lu , J. Zhou , T. Nakayama , R. Yang , and B. Li , “Interfacial Thermal Conductance Across Metal‐Insulator/Semiconductor Interfaces due to Surface States,” Physical Review B 93 (2016): 085433, 10.1103/PhysRevB.93.085433.

[197] S. Goel , X. Luo , A. Agrawal , and R. L. Reuben , “Diamond Machining of Silicon: A Review of Advances in Molecular Dynamics Simulation,” International Journal of Machine Tools and Manufacture 88 (2015): 131–164, 10.1016/j.ijmachtools.2014.09.013.

[198] L.‐Y. Li , L. Qiu , N. Cao , et al., “Revealing the Mechanism of Significant Enhancement in Interfacial Thermal Transport in Silicon‐Based Ceramic Crystalline/Amorphous Matrix Composite Phase Change Materials,” Rare Metals 44 (2025): 4107–4118, 10.1007/s12598-025-03301-2.

[199] X. Wang , X. Wang , Y. Tong , and Y. Wang , “Enhancing Interfacial Thermal Conductivity of Copper‐Carbon Nanotube Array Composite via Metallic Bonding: Molecular Dynamics Simulations,” Chemical Physics 584 (2024): 112341, 10.1016/j.chemphys.2024.112341.

[200] X.‐D. Zhang , G. Yang , and B.‐Y. Cao , “Bonding‐Enhanced Interfacial Thermal Transport: Mechanisms, Materials, and Applications,” Advanced Materials Interfaces 9 (2022): 2200078, 10.1002/admi.202200078.

[201] Z. Liu , X. Sun , J. Xie , X. Zhang , and J. Li , “Interfacial Thermal Transport Properties and Its Effect on Thermal Conductivity of Functionalized BNNS/Epoxy Composites,” International Journal of Heat and Mass Transfer 195 (2022): 123031, 10.1016/j.ijheatmasstransfer.2022.123031.

[202] Y. Jiang , X. Shi , Y. Feng , S. Li , X. Zhou , and X. Xie , “Enhanced Thermal Conductivity and Ideal Dielectric Properties of Epoxy Composites Containing Polymer Modified Hexagonal Boron Nitride,” Composites Part A: Applied Science and Manufacturing 107 (2018): 657–664, 10.1016/j.compositesa.2018.02.016.

[203] T. Feng , J. Cui , M. Ou , et al., “0D‐2D Nanohybrids Based on Binary Transitional Metal Oxide Decorated Boron Nitride Enabled Epoxy Resin Efficient Flame Retardant Coupled with Enhanced Thermal Conductivity at Ultra‐Low Additions,” Composites Communications 41 (2023): 101649, 10.1016/j.coco.2023.101649.

[204] W. Shen , W. Wu , C. Liu , Z. Wang , and Z. Huang , “Achieving a High Thermal Conductivity for Segregated BN/PLA Composites via Hydrogen Bonding Regulation Through Cellulose Network,” Polymers for Advanced Technologies 31 (2020): 1911–1920, 10.1002/pat.4916.

[205] Z. Ji , W. Liu , C. Ouyang , and Y. Li , “High Thermal Conductivity Thermoplastic Polyurethane/Boron Nitride/Liquid Metal Composites: The Role of the Liquid Bridge at the Filler/Filler Interface,” Materials Advances 2 (2021): 5977–5985, 10.1039/D1MA00637A.

[206] Q. Chi , X. Zhang , X. Wang , et al., “High Thermal Conductivity of Epoxy‐Based Composites Utilizing 3D Porous Boron Nitride Framework,” Composites Communications 33 (2022): 101195, 10.1016/j.coco.2022.101195.

[207] W. Yang , M. Zhang , K. Wang , et al., “Reducing Interfacial Thermal Resistance between Epoxy and Alumina via Interfacial Engineering,” Physica Status Solidi (a) 220 (2023): 2200800, 10.1002/pssa.202200800.

[208] S. Bakalakos , I. Kalogeris , and V. Papadopoulos , “An Extended Finite Element Method Formulation for Modeling Multi‐Phase Boundary Interactions in Steady State Heat Conduction Problems,” Composite Structures 258 (2021): 113202, 10.1016/j.compstruct.2020.113202.

[209] M. S. Dresselhaus , G. Dresselhaus , R. Saito , and A. Jorio , “Raman Spectroscopy of Carbon Nanotubes,” Physics Reports 409 (2005): 47–99, 10.1016/j.physrep.2004.10.006.

[210] J.‐H. Kim , A. R. T. Nugraha , L. G. Booshehri , et al., “Coherent Phonons in Carbon Nanotubes and Graphene,” Chemical Physics 413 (2013): 55–80, 10.1016/j.chemphys.2012.09.017. 23478856

[211] J. Chen , J. He , D. Pan , et al., “Emerging Theory and Phenomena in Thermal Conduction: A Selective Review,” Science China Physics, Mechanics & Astronomy 65 (2022): 117002, 10.1007/s11433-022-1952-3.

[212] A. Badakhsh , Y.‐M. Lee , K. Y. Rhee , C. W. Park , K.‐H. An , and B.‐J. Kim , “Improvement of Thermal, Electrical and Mechanical Properties of Composites Using a Synergistic Network of Length Controlled‐CNTs and Graphene Nanoplatelets,” Composites Part B: Engineering 175 (2019): 107075, 10.1016/j.compositesb.2019.107075.

[213] Q. Kong , L. Bodelot , B. Lebental , et al., “Novel Three‐Dimensional Carbon Nanotube Networks as High Performance Thermal Interface Materials,” Carbon 132 (2018): 359–369, 10.1016/j.carbon.2018.02.052.

[214] M. Safdari and M. S. Al‐Haik , “Synergistic Electrical and Thermal Transport Properties of Hybrid Polymeric Nanocomposites Based on Carbon Nanotubes and Graphite Nanoplatelets,” Carbon 64 (2013): 111–121, 10.1016/j.carbon.2013.07.042.

[215] L. Jing , M. K. Samani , B. Liu , et al., “Thermal Conductivity Enhancement of Coaxial Carbon@Boron Nitride Nanotube Arrays,” ACS Applied Materials & Interfaces 9 (2017): 14555–14560, 10.1021/acsami.7b02154. 28429587

[216] X. He , Y. Huang , C. Wan , et al., “Enhancing Thermal Conductivity of Polydimethylsiloxane Composites Through Spatially Confined Network of Hybrid Fillers,” Composites Science and Technology 172 (2019): 163–171, 10.1016/j.compscitech.2019.01.009.

[217] K. Kalaitzidou , H. Fukushima , and L. T. Drzal , “Multifunctional Polypropylene Composites Produced by Incorporation of Exfoliated Graphite Nanoplatelets,” Carbon 45 (2007): 1446–1452, 10.1016/j.carbon.2007.03.029.

[218] S. K. Bhattacharya and A. C. D. Chaklader , “Review on Metal‐Filled Plastics. Part 2. Thermal Properties,” Polymer‐Plastics Technology and Engineering 20 (1983): 35–59, 10.1080/03602558308067736.

[219] S. Pradhan , R. Lach , H. H. Le , W. Grellmann , H.‐J. Radusch , and R. Adhikari , “Effect of Filler Dimensionality on Mechanical Properties of Nanofiller Reinforced Polyolefin Elastomers,” International Scholarly Research Notices 2013 (2013): 284504, 10.1155/2013/284504.

[220] W. Si , J. Sun , X. He , et al., “Enhancing Thermal Conductivity via Conductive Network Conversion from High to Low Thermal Dissipation in Polydimethylsiloxane Composites,” Journal of Materials Chemistry C 8 (2020): 3463–3475, 10.1039/C9TC06968B.

[221] K. Pashayi , H. R. Fard , F. Lai , S. Iruvanti , J. Plawsky , and T. Borca‐Tasciuc , “High Thermal Conductivity Epoxy‐Silver Composites Based on Self‐Constructed Nanostructured Metallic Networks,” Journal of Applied Physics 111 (2012): 104310, 10.1063/1.4716179.

[222] B. L. Zhu , H. Zheng , J. Wang , J. Ma , J. Wu , and R. Wu , “Tailoring of Thermal and Dielectric Properties of LDPE‐Matrix Composites by the Volume Fraction, Density, and Surface Modification of Hollow Glass Microsphere Filler,” Composites Part B: Engineering 58 (2014): 91–102, 10.1016/j.compositesb.2013.10.029.

[223] Z. Qi , W. Shen , R. Li , et al., “AlN/Diamond Interface Nanoengineering for Reducing Thermal Boundary Resistance by Molecular Dynamics Simulations,” Applied Surface Science 615 (2023): 156419, 10.1016/j.apsusc.2023.156419.

[224] E. Lee , T. Zhang , T. Yoo , Z. Guo , and T. Luo , “Nanostructures Significantly Enhance Thermal Transport Across Solid Interfaces,” ACS Applied Materials & Interfaces 8 (2016): 35505–35512, 10.1021/acsami.6b12947. 27983798

[225] W. Luo , N. Wang , W. Lian , E. Yin , and Q. Li , “Enhancing Interfacial Thermal Transport by Nanostructures: Monte Carlo Simulations with Ab Initio Phonon Properties,” Fluid Dynamics arXiv (2024): 240619068, 10.48550/arXiv.2406.19068.

[226] Z. Wang , L. Wei , X. Wang , et al., “Interfacial Regulation to Improve Interface Heat Transfer of Al/Diamond Composites Based on Molecular Dynamics Simulations,” Diamond and Related Materials 153 (2025): 112029, 10.1016/j.diamond.2025.112029.

[227] W. Park , A. Sood , J. Park , M. Asheghi , R. Sinclair , and K. E. Goodson , “Enhanced Thermal Conduction through Nanostructured Interfaces,” Nanoscale and Microscale Thermophysical Engineering 21 (2017): 134–144, 10.1080/15567265.2017.1296910.

[228] Z. Cheng , T. Bai , J. Shi , et al., “Tunable Thermal Energy Transport Across Diamond Membranes and Diamond–Si Interfaces by Nanoscale Graphoepitaxy,” ACS Applied Materials & Interfaces 11 (2019): 18517–18527, 10.1021/acsami.9b02234. 31042348

[229] Y.‐C. Hua and B.‐Y. Cao , “Study of Phononic Thermal Transport Across Nanostructured Interfaces Using Phonon Monte Carlo Method,” International Journal of Heat and Mass Transfer 154 (2020): 119762, 10.1016/j.ijheatmasstransfer.2020.119762.

[230] Q. Li , F. Liu , S. Hu , et al., “Inelastic Phonon Transport Across Atomically Sharp Metal/Semiconductor Interfaces,” Nature Communications 13 (2022): 4901, 10.1038/s41467-022-32600-w. PMC9392776 35987993

[231] C. A. Polanco and L. Lindsay , “Phonon Thermal Conductance Across GaN‐AlN Interfaces from First Principles,” Physical Review B 99 (2019): 075202, 10.1103/PhysRevB.99.075202.

[232] R. Wu , X. Zhao , and Y. Liu , “Atomic Insights of Cu Nanoparticles Melting and Sintering Behavior in CuCu Direct Bonding,” Materials & Design 197 (2021): 109240, 10.1016/j.matdes.2020.109240.

[233] R. Luo , D. Hu , C. Qian , et al., “Molecular Dynamics Simulations on Mechanical Behaviors of Sintered Nanocopper in Power Electronics Packaging,” Microelectronics Reliability 152 (2024): 115284, 10.1016/j.microrel.2023.115284.

[234] D. Hu , C. Qian , X. Liu , et al., “High Temperature Viscoplastic Deformation Behavior of Sintered Nanocopper Paste Used in Power Electronics Packaging: Insights from Constitutive and Multi‐Scale Modelling,” Journal of Materials Research and Technology 26 (2023): 3183–3200, 10.1016/j.jmrt.2023.08.086.

[235] S. Liu , S. Zhao , D. Zhang , et al., “Molecular Dynamics Analysis of the Solid‐State Bonding Mechanism and High Strain Rate Response for (1 1 1)‐Oriented Nanotwinned Silver,” ACS Applied Materials & Interfaces 17, no. 15 (2025): 23308–23321, 10.1021/acsami.5c00590. 40171850

[236] Z. Zhang , G. Fu , B. Wan , Y. Su , and M. Jiang , “Research on Sintering Process and Thermal Conductivity of Hybrid Nanosilver Solder Paste Based on Molecular Dynamics Simulation,” Microelectronics Reliability 126 (2021): 114203, 10.1016/j.microrel.2021.114203.

[237] Q. Jia , G. Zou , H. Zhang , et al., “Sintering Mechanism of Ag‐Pd Nanoalloy Film for Power Electronic Packaging,” Applied Surface Science 554 (2021): 149579, 10.1016/j.apsusc.2021.149579. 32174102

[238] X. Hu , J. Huang , R. Poelma , W. Driel , and Z. G. D , “Sintering Process Simulation of Ag Nanoparticles by Phase Field Method,” in 2024 25th International Conference on Thermal, Mechanical and Multi‐Physics Simulation and Experiments in Microelectronics and Microsystems (EuroSimE) , (IEEE, 2024), 10.1109/EuroSimE60745.2024.10491517.

[239] M. Xue and M. Yi , “Phase‐Field Simulation of Sintering Process: A Review,” Computer Modeling in Engineering & Sciences 140 (2024): 1165–1204, 10.32604/cmes.2024.049367.

[240] K. Ahmed , J. Pakarinen , T. Allen , and A. El‐Azab , “Phase Field Simulation of Grain Growth in Porous Uranium Dioxide,” Journal of Nuclear Materials 446 (2014): 90–99, 10.1016/j.jnucmat.2013.11.036.

[241] K. Chockalingam , V. G. Kouznetsova , O. van der Sluis , and M. G. D. Geers , “2D Phase Field Modeling of Sintering of Silver Nanoparticles,” Computer Methods in Applied Mechanics and Engineering 312 (2016): 492–508, 10.1016/j.cma.2016.07.002.

[242] X. Chen , L. Yang , Y. Zhang , et al., “A Comprehensive Model for Spinodal Decomposition in Ag–Cu Alloys Based on Phase‐Field Theory and In Situ TEM,” ACS Applied Materials & Interfaces 17 (2025): 54263–54281, 10.1021/acsami.5c13603. 40936195

[243] C. Cao , M. Yang , C. Liang , et al., “A Phase‐Field Model of Electrochemical Migration for Silver‐Based Conductive Adhesives,” Electrochimica Acta 471 (2023): 143388, 10.1016/j.electacta.2023.143388.

[244] J. W. Cahn , “On Spinodal Decomposition,” Acta Metallurgica 9 (1961): 795–801, 10.1016/0001-6160(61)90182-1.

[245] J. W. Cahn and S. M. Allen , “A Microscopic Theory for Domain Wall Motion and Its Experimental Verification in Fe‐Al Alloy Domain Growth Kinetics,” in The Selected Works of John W. Cahn, (John Wiley & Sons, 1998), 10.1002/9781118788295.ch36.

[246] V. Kumar , Z. Z. Fang , and P. C. Fife , “Phase Field Simulations of Grain Growth During Sintering of Two Unequal‐Sized Particles,” Materials Science and Engineering: A 528 (2010): 254–259, 10.1016/j.msea.2010.08.061.

[247] J. Deng , “A Phase Field Model of Sintering with Direction‐Dependent Diffusion,” Materials Transactions 53 (2012): 385–389, 10.2320/matertrans.M2011317.

[248] S. Biswas , D. Schwen , J. Singh , and V. Tomar , “A Study of the Evolution of Microstructure and Consolidation Kinetics During Sintering Using a Phase Field Modeling Based Approach,” Extreme Mechanics Letters 7 (2016): 78–89, 10.1016/j.eml.2016.02.017.

[249] Y. U. Wang , “Computer Modeling and Simulation of Solid‐State Sintering: A Phase Field Approach,” Acta Materialia 54 (2006): 953–961, 10.1016/j.actamat.2005.10.032.

[250] S. Biswas , D. Schwen , and V. Tomar , “Implementation of a Phase Field Model for Simulating Evolution of Two Powder Particles Representing Microstructural Changes During Sintering,” Journal of Materials Science 53 (2018): 5799–5825, 10.1007/s10853-017-1846-3.

[251] S. Liang , C. Liu , H. Jiang , and Z. Zhong , “Investigation of Electrical–Thermal–Mechanical Effects in Electric‐Assisted Silver Sintering Process through Phase Field Modeling,” IEEE Transactions on Components, Packaging and Manufacturing Technology 13 (2023): 1764–1769, 10.1109/TCPMT.2023.3327375.

[252] S. Y. Hu and L. Q. Chen , “A Phase‐Field Model for Evolving Microstructures with Strong Elastic Inhomogeneity,” Acta Materialia 49 (2001): 1879–1890, 10.1016/S1359-6454(01)00118-5.

[253] J. W. Cahn , “Phase Separation by Spinodal Decomposition in Isotropic Systems,” Journal of Chemical Physics 42 (1965): 93–99, 10.1063/1.1695731.

[254] D. J. Seol , S. Y. Hu , Y. L. Li , J. Shen , K. H. Oh , and L. Q. Chen , “Computer Simulation of Spinodal Decomposition in Constrained Films,” Acta Materialia 51 (2003): 5173–5185, 10.1016/S1359-6454(03)00378-1.

[255] D. J. Seol , S. Y. Hu , K. H. Oh , and L. Q. Chen , “Effect of Substrate Constraint on Spinodal Decomposition in an Elastically Inhomogeneous Thin Film,” Metals and Materials International 10 (2004): 429–434, 10.1007/BF03027344.

[256] J. A. Stewart and R. Dingreville , “Microstructure Morphology and Concentration Modulation of Nanocomposite Thin‐Films During Simulated Physical Vapor Deposition,” Acta Materialia 188 (2020): 181–191, 10.1016/j.actamat.2020.02.011.

[257] Y. Lu , B. Derby , H. Sriram , et al., “Microstructure Development and Morphological Transition During Deposition of Immiscible Alloy Films,” Acta Materialia 220 (2021): 117313, 10.1016/j.actamat.2021.117313.

[258] J. L. Li , Z. Li , Q. Wang , C. Dong , and P. K. Liaw , “Phase‐Field Simulation of Coherent BCC/B2 Microstructures in High Entropy Alloys,” Acta Materialia 197 (2020): 10–19, 10.1016/j.actamat.2020.07.030.

[259] K. Kadirvel , H. L. Fraser , and Y. Wang , “Microstructural Design via Spinodal‐Mediated Phase Transformation Pathways in High‐Entropy Alloys (HEAs) Using Phase‐Field Modelling,” Acta Materialia 243 (2023): 118438, 10.1016/j.actamat.2022.118438.

[260] S. R. Koneru , K. Kadirvel , H. Fraser , and Y. Wang , “Microstructural Engineering by Heat Treatments of Multi‐Principal Element Alloys via Spinodal Mediated Phase Transformation Pathways,” Acta Materialia 258 (2023): 119198, 10.1016/j.actamat.2023.119198.

[261] L. Chen , H. W. Zhang , L. Y. Liang , et al., “Modulation of Dendritic Patterns During Electrodeposition: A Nonlinear Phase‐Field Model,” Journal of Power Sources 300 (2015): 376–385, 10.1016/j.jpowsour.2015.09.055.

[262] A. Jana , S. I. Woo , K. S. N. Vikrant , and R. E. García , “Electrochemomechanics of Lithium Dendrite Growth,” Energy & Environmental Science 12 (2019): 3595–3607, 10.1039/C9EE01864F.

[263] Z. Mu , Z. Guo , and Y.‐H. Lin , “Simulation of 3‐D Lithium Dendritic Evolution Under Multiple Electrochemical States: A Parallel Phase Field Approach,” Energy Storage Materials 30 (2020): 52–58, 10.1016/j.ensm.2020.04.011.

[264] C. Lin , K. Liu , H. Ruan , and B. Wang , “Mechano‐Electrochemical Phase Field Modeling for Formation and Modulation of Dendritic Pattern: Application to Uranium Recovery from Spent Nuclear Fuel,” Materials & Design 213 (2022): 110322, 10.1016/j.matdes.2021.110322.

[265] B. Illés , B. Medgyes , K. Dušek , D. Bušek , A. Skwarek , and A. Géczy , “Numerical Simulation of Electrochemical Migration of Cu Based on the Nernst‐Plank Equation,” International Journal of Heat and Mass Transfer 184 (2022): 122268, 10.1016/j.ijheatmasstransfer.2021.122268.

[266] S. Zhao , M. Yang , Y. Liu , et al., “The Anti‐Electrochemical Migration Mechanism of Ag‐Based Transient Liquid‐Phase Electrically Conductive Adhesive: Experimental and Phase‐Field Study,” Applied Surface Science 696 (2025): 162998, 10.1016/j.apsusc.2025.162998.

[267] Y. Liu , S. Irving , T. Luk , and D. Kinzer , “Trends of Power Electronic Packaging and Modeling,” in 2008 10th Electronics Packaging Technology Conference (IEEE, 2008), 10.1109/EPTC.2008.4763404.

[268] Y. Wang , H. Liu , L. Huo , et al., “Research on the Reliability of Advanced Packaging Under Multi‐Field Coupling: A Review,” Micromachines 15 (2024): 422, 10.3390/mi15040422. 38675234 PMC11051953

[269] Y. Liu , Power Electronic Packaging: Design, Assembly Process, Reliability and Modeling, (Springer Science & Business Media, 2012), 10.1007/978-1-4614-1053-9.

[270] G. Mauromicale , M. Calabretta , G. Scarcella , G. Scelba , and A. Sitta , “Multi‐Physics Models of a Low‐Voltage Power Semiconductor System‐in‐Package for Automotive Applications,” Journal of Electronic Packaging 145 (2023): 031003, 10.1115/1.4056413.

[271] J. Song , S. Hu , and Y. Liu , “A Laser Assisted Bonding Process Design with Silver‐Indium Transient Liquid Phase Method for the Infrared Detectors Hermetic Packaging,” in 2022 23rd International Conference on Electronic Packaging Technology (ICEPT) (IEEE, 2022), 10.1109/ICEPT56209.2022.9873195.

[272] S. Hu , J. Song , Y. Liu , et al., “A Laser‐Assisted Thermal Gradient Transient Liquid Phase Bonding Process Design for Thermally Sensitive Components in Hermetic Packaging,” IEEE Transactions on Components, Packaging and Manufacturing Technology 14 (2024): 328–341, 10.1109/TCPMT.2024.3355159.

[273] Y. Jia , F. Xiao , Y. Duan , Y. Luo , B. Liu , and Y. Huang , “PSpice‐COMSOL‐Based 3‐D Electrothermal–Mechanical Modeling of IGBT Power Module,” IEEE Journal of Emerging and Selected Topics in Power Electronics 8 (2020): 4173–4185, 10.1109/JESTPE.2019.2935037.

[274] X. Zhao , Y. Xu , and D. C. Hopkins , “Advanced Multi‐Physics Simulation for High Performance Power Electronic Packaging Design,” in 2016 International Symposium on 3D Power Electronics Integration and Manufacturing (3D‐PEIM) (IEEE, 2016), 10.1109/3DPEIM.2016.8048203.

[275] B. Yu and Y. Gao , “Multi‐Physics Fields Simulations and Optimization of Solder Joints in Advanced Electronic Packaging,” Chips 1 (2022): 191–209, 10.3390/chips1030013.

[276] D. Li , M. Packwood , and F. Qi , “ 2016 17th International Conference on Thermal, Mechanical and Multi‐Physics Simulation and Experiments in Microelectronics and Microsystems (EuroSimE) ,” (IEEE, 2016), 10.1109/EuroSimE.2016.7463369.

[277] Y. Liu and D. Kinzer , “Challenges of Power Electronic Packaging and Modeling,” in 2011 12th International Conference on Thermal, Mechanical & Multi‐Physics Simulation and Experiments in Microelectronics and Microsystems (IEEE, 2011), 10.1109/ESIME.2011.5765799.

[278] J. A. Elliott , “Novel Approaches to Multiscale Modelling in Materials Science,” International Materials Reviews 56 (2011): 207–225, 10.1179/1743280410Y.0000000002.

[279] K. Momeni , Y. Ji , Y. Wang , et al., “Multiscale Computational Understanding and Growth of 2D Materials: A Review,” npj Computational Materials 6 (2020): 22, 10.1038/s41524-020-0280-2.

[280] S. Kohlhoff , P. Gumbsch , and H. F. Fischmeister , “Crack Propagation in b.c.c. Crystals Studied with a Combined Finite‐Element and Atomistic Model,” Philosophical Magazine A 64 (1991): 851–878, 10.1080/01418619108213953.

[281] R. E. Rudd , “Coarse‐Grained Molecular Dynamics: Nonlinear Finite Elements and Finite Temperature,” Physical Review B 72 (2005): 144104, 10.1103/PhysRevB.72.144104.

[282] R. U. Patil , B. K. Mishra , I. V. Singh , and T. Q. Bui , “A New Multiscale Phase Field Method to Simulate Failure in Composites,” Advances in Engineering Software 126 (2018): 9–33, 10.1016/j.advengsoft.2018.08.010.

[283] D. Molnar , R. Mukherjee , A. Choudhury , et al., “Multiscale Simulations on the Coarsening of Cu‐Rich Precipitates in α‐Fe Using Kinetic Monte Carlo, Molecular Dynamics and Phase‐Field Simulations,” Acta Materialia 60 (2012): 6961–6971, 10.1016/j.actamat.2012.08.051.

[284] W. E , H. Lei , P. Xie , and L. Zhang , “Machine Learning‐Assisted Multi‐Scale Modeling,” Journal of Mathematical Physics 64 (2023): 071101, 10.1063/5.0149861.

[285] C. Na , S. Shin , D. Lee , et al., “Data‐Driven Engineering and Analysis of Polymer Composites with High Thermal Conductivity,” Composites Science and Technology 272 (2025): 111400, 10.1016/j.compscitech.2025.111400.

[286] M. Capone , M. Romanelli , D. Castaldo , et al., “A Vision for the Future of Multiscale Modeling,” ACS Physical Chemistry Au 4 (2024): 202–225, 10.1021/acsphyschemau.3c00080. 38800726 PMC11117712

[287] D. M. Kochmann and J. S. Amelang , “The Quasicontinuum Method: Theory and Applications,” in Multiscale Materials Modeling for Nanomechanics, ed. C. R. Weinberger and G. J. Tucker (Springer International Publishing, 2016), 10.1007/978-3-319-33480-6_5.

[288] O. Rokoš , R. H. J. Peerlings , and J. Zeman , “eXtended Variational Quasicontinuum Methodology for Lattice Networks with Damage and Crack Propagation,” Computer Methods in Applied Mechanics and Engineering 320 (2017): 769–792, 10.1016/j.cma.2017.03.042.

[289] A. Muixí , O. Marco , A. Rodríguez‐Ferran , and S. Fernández‐Méndez , “A Combined XFEM Phase‐Field Computational Model for Crack Growth without Remeshing,” Computational Mechanics 67 (2021): 231–249, 10.1007/s00466-020-01929-8.

[290] R. Perera and V. Agrawal , “Multiscale Graph Neural Networks with Adaptive Mesh Refinement for Accelerating Mesh‐Based Simulations,” Computer Methods in Applied Mechanics and Engineering 429 (2024): 117152, 10.1016/j.cma.2024.117152.

[291] P. C. H. Nguyen , J. B. Choi , H. S. Udaykumar , and S. Baek , “Challenges and Opportunities for Machine Learning in Multiscale Computational Modeling,” Journal of Computing and Information Science in Engineering 23 (2023): 060808, 10.1115/1.4062495.

[292] D. Bishara , Y. Xie , W. Liu , and S. Li , “A State‐of‐the‐Art Review on Machine Learning‐Based Multiscale Modeling, Simulation, Homogenization and Design of Materials,” Archives of Computational Methods in Engineering 30 (2023): 191–222, 10.1007/s11831-022-09795-8.

[293] J. Linghu , H. Dong , Y. Nie , and J. Cui , “Higher‐Order Multi‐Scale Deep Ritz Method (HOMS‐DRM) and Its Convergence Analysis for Solving Thermal Transfer Problems of Composite Materials,” Computational Mechanics 75 (2025): 71–95, 10.1007/s00466-024-02491-3.

[294] A. K. Chew , M. A. F. Afzal , Z. Kaplan , et al., “Leveraging High‐Throughput Molecular Simulations and Machine Learning for the Design of Chemical Mixtures,” npj Computational Materials 11 (2025): 72, 10.1038/s41524-025-01552-2.

[295] Y. Liu , W. Hong , and B. Cao , “Machine Learning for Predicting Thermodynamic Properties of Pure Fluids and Their Mixtures,” Energy 188 (2019): 116091, 10.1016/j.energy.2019.116091.

[296] H. Zhou and T. Feng , “Theoretical Upper Limits of the Thermal Conductivity of Si_3_N_4_ ,” Applied Physics Letters 122 (2023): 182203, 10.1063/5.0149298.

[297] S. Jasmee , G. Omar , S. S. C. Othaman , N. A. A. Masripan , and H. Hamid , “Interface Thermal Resistance and Thermal Conductivity of Polymer Composites at Different Types, Shapes, and Sizes of Fillers: A Review,” Polymer Composites (2021): 2629–2652, 10.1002/pc.26029.

[298] S. Amini Niaki , E. Haghighat , T. Campbell , A. Poursartip , and R. Vaziri , “Physics‐Informed Neural Network for Modelling the Thermochemical Curing Process of Composite‐Tool Systems During Manufacture,” Computer Methods in Applied Mechanics and Engineering 384 (2021): 113959, 10.1016/j.cma.2021.113959.

[299] A. Michaloglou , I. Papadimitriou , I. Gialampoukidis , S. Vrochidis , and I. Kompatsiaris , “Physics‐Informed Neural Networks in Materials Modeling and Design: A Review,” Archives of Computational Methods in Engineering (2025), 10.1007/s11831-025-10448-9.

[300] B. Liu , Y. Wang , T. Rabczuk , T. Olofsson , and W. Lu , “Multi‐Scale Modeling in Thermal Conductivity of Polyurethane Incorporated with Phase Change Materials Using Physics‐Informed Neural Networks,” Renewable Energy 220 (2024): 119565, 10.1016/j.renene.2023.119565.

